# Characterization and Metabolism Effect of Seed Endophytic Bacteria Associated With Peanut Grown in South China

**DOI:** 10.3389/fmicb.2019.02659

**Published:** 2019-11-13

**Authors:** Limei Li, Zhi Zhang, Shiyu Pan, Ling Li, Xiaoyun Li

**Affiliations:** Guangdong Provincial Key Laboratory of Biotechnology for Plant Development, School of Life Sciences, South China Normal University, Guangzhou, China

**Keywords:** seed endophytic bacteria, P.g.YMR3, peanut (*Arachis hypogaea*), plant growth promotion, symbiotic relationship, vertical transmission

## Abstract

Endophytes are considered to be excellent biocontrol agents and biofertilizers, and are associated with plant growth promotion and health. In particular, seed-endophytic bacteria benefit the host plant’s progeny via vertical transmission, and can play a role in plant growth and defense. However, seed-associated endophytic bacteria have not been fully explored, with very little known about how they interact with peanut (*Arachis hypogaea*), for example. Here, 10 genera of endophytic bacteria were isolated from the root tips of peanut seedlings grown either aseptically or in soil. Forty-two bacterial colonies were obtained from peanut seedlings grown in soil, mostly from the genus *Bacillus*. Eight colonies were obtained from aseptic seedling root tips, including *Bacillus* sp., *Paenibacillus* sp., and *Pantoea dispersa*. Four *Bacillus* peanut strains GL1–GL4 (B.p.GL1-GL4) produced bio-films, while B.p.GL2 and *Paenibacillus glycanilyticus* YMR3 (P.g.YMR3) showed strong amylolytic capability, enhanced peanut biomass, and increased numbers of root nodules. Conversely, *P. dispersa* YMR1 (P.d.YMR1) caused peanut plants to wilt. P.g.YMR3 was distributed mainly around or inside vacuoles and was transmitted to the next generation through gynophores and ovules. Hexanoate, succinate, and jasmonic acid (JA) accumulated in peanut root tips after incubation with P.g.YMR3, but linolenate content decreased dramatically. This suggests that strain P.g.YMR3 increases JA content (14.93-fold change) and modulates the metabolism of peanut to facilitate nodule formation and growth. These findings provide new insight into plant–seed endophytic bacterial interactions in peanut.

## Introduction

The peanut (*Arachis hypogaea* L.) is a member of the Fabaceae family, and is of great importance worldwide as a food, oil, and cash crop (Sobolev et al., 2013). Diverse microbes exist and interact with peanuts, most often associated with N_2_-fixation and biotic stress (Chen et al., 2019). Many fungal and bacterial diseases, such as leaf blight (*Alternaria tenuissima*), crown and seed rot (*Aspergillus niger*), root rot (*Fusarium moniliforme*), pod rot (*Rhizoctonia solani*), and wilt (*Ralstonia solanacearum*), occur in peanuts grown in South China because of the warm and wet weather (Rojo et al., 2007; Haggag and Timmusk, 2008; Vargas Gil et al., 2008; Wang and Liang, 2014; Jiang et al., 2017; Sobolev et al., 2018). Endophytic microbes, especially seed endophytic bacteria, are considered to be excellent biofertilizers and biocontrol agents in agriculture. They are not only associated with plant growth promotion and health, but can also transmit these benefits from one generation of plants to the next (Huang and Pang, 2017; Chen et al., 2019). Several endophytic bacteria, including *Enterobacter* sp. J49, *Methylobacterium* spp., *Sphingomonas* spp., *Bacillus* spp., *Curtobacterium* spp. and *Paenibacillus* spp., have been shown to promote peanut growth (Sundin and Jacobs, 1999; Madhaiyan et al., 2006; Haggag and Timmusk, 2008; Sadaf et al., 2016; Liu et al., 2017; Ludueña et al., 2018; Chen et al., 2019; Prestes et al., 2019). For example, *Bacillus velezensis* LDO2 promoted the growth of peanut plants and inhibited the growth of *Aspergillus flavus* mycelia (Chen et al., 2019). Endophytic microbes influence plant growth and defenses by producing various phytohormones [e.g., 3-indoleacetic acid (IAA) and cytokinins], volatile organic compounds (VOCs, e.g., 3-hydroxy-2-butanone, 2,3-butendiol, and acetoin), or other secondary metabolites (e.g., spermidine, phytic acid, soluble phosphorus, and trehalose) (Dardanelli et al., 2000; Arguelles-Arias et al., 2009; Fincheira and Quiroz, 2018; Ludueña et al., 2018; Zhou et al., 2018). However, the biodiversity and behavior of seed-associated bacteria have not been thoroughly investigated. To reside in a seed and adapt to severe environmental conditions during seed maturation and then to resume growth after long-term survival, endophytic bacteria utilize several special properties of the seed (Johnston-Monje and Raizada, 2011; Truyens et al., 2014), such as formation of endospores, cell motility, tolerance to high osmotic pressure, and amylase activity, and can also transmit such benefits to the host plant’s progeny (Mano et al., 2006; Compant et al., 2011; Shahzad et al., 2018). However, very little is known about endophyte behavior and vertical transmission in peanut.

In this study, 10 genera of endophytic bacteria were isolated from aseptically grown or soil-grown seedling root meristems in peanut under low pH (3.5–5.0) conditions. Their characteristics and plant growth-promoting properties were investigated.

## Materials and Methods

### Isolation and Identification of Endophytic Bacteria

Endophytic bacteria were isolated according to the method described and partly modified by Huang and Pang (2017). For isolation of common endophytic bacteria, seeds of the peanut cultivar Yueyou7 originating in South China were grown in field soil. Then, 1 g soil-grown seedling root tips (3–4 cm, non-nodulating, Supplementary Figure S1) were picked and surface-sterilized by immersing in 10% sodium hypochlorite for 10 min and rinsed six times with sterile distilled water. Thereafter, the root tips were placed in 75% ethanol for 1 min and rinsed three times with distilled water; this was performed twice. The effect of surface sterilization was checked by spreading the final rinse water (200 μL) onto nutrient agar plates and incubating at ±35°C for 48 h. Sterilized root tips were then mashed aseptically with 10 mL phosphate-buffered saline (PBS; NaCl 8 g, KCl 0.2 g, Na_2_HPO_4_ 1.42 g, KH_2_PO_4_ 0.27 g dissolved in 1 L sterile ddH_2_O, pH 7.4) and allowed to stand for 1 min. The resultant supernatant was diluted and plated on NA plates of different pH (pH 3.5, 4.0, 4.5, and 5.0) and grown at ±35°C for 48–72 h. Single colonies were isolated and PCR was performed using primers against the variable region of 16S rDNA: 27-F: AGAGTTTGATCCTGGCTCAG; 1492-R: GGTTACCTTGTTACGACTT (DeLong, 1992). The PCR products were sequenced and analyzed by BLAST for 16S ribosomal RNA sequences (Bacteria and Archaea) in the NCBI database, as described previously (Sobolev et al., 2013).

To isolate seed-associated endophytic bacteria, Yueyou7 seeds were surface-sterilized by 10% sodium hypochlorite and 75% ethanol as described above. Thereafter, the seeds were rinsed three time with distilled water and cultivated on MS medium in a bottle for 2 weeks. Then, 1 g aseptic seedling root tips were picked and mashed aseptically with 10 mL PBS, and allowed to stand for 1 min. The resultant supernatant was diluted and plated on NA plates of different pH (pH 3.5, 4.0, 4.5, and 5.0) and incubated at ±35°C for 48–72 h. Single monoclonal was isolated and PCR was performed as described above. The clonal, characterized endophytes were stored at −80°C prior to further analysis.

The morphological characteristics of endophytic bacterial colonies were noted and photographed using a stereoscopic microscope. For starch hydrolysis, 2 μL endophytic bacteria were added to the starch plate and incubated at ±35°C for 48 h, after which drops of 1% KI were also added to the endophytic colonies; the stained plates were then photographed.

### Plant Growth-Promoting Assay

To investigate peanut growth promotion, soil was sterilized for 20 min at 120°C, while the soil in the control group (CT^+^) was not sterilized. Then, peanut seedlings grown aseptically for 2 weeks were transplanted into the soil. After 1 week, frozen stocks of endophytic bacteria were revived and propagated (220 rpm at 28°C) for 8–16 h, then centrifuged at 6000 rpm for 10 min, and resuspended in sterile distilled water, adjusting the OD_600_ to 0.1. Thereafter, 30 mL bacterial suspension was inoculated into soil around the peanut seedling along the 1 mL pipette. Distilled water without bacteria was used as a control group (CT^–^). All plants were grown in the greenhouse for 1.5–2 months. Then the plants were harvested, and the plant biomass, number of nodules, length and perimeter of stem, and leaf area were measured.

### Cellular Distribution of Endophytic Bacteria Indicated by eGFP Marker

To observe endophytic bacteria inside peanut tissues, the eGFP coding sequence was amplified from the pCanG vector using specific primers:

GFP-F *Bam*HI: AGGCTGGTTCCGCGTGGATCCATGGT GAGCAAGGGCGAGGAG;
GFP-R *Hin*dIII: GTTAGCAGCCGGATCAAGCTTTTACTTG TACAGCTCGTCCATGCCG.

Thereafter, the PCR products were cloned into pRHisMBP, and recombinant plasmids were transformed into endophytic bacteria by the CaCl_2_ and liquid nitrogen freeze-thawing methods. Expression of eGFP in bacteria was induced by 0.5 mM IPTG at 37°C for 6 h, while expression of enhanced green fluorescent protein (eGFP) in peanut roots was induced by 0.5 mM isopropylthio-β-galactoside (IPTG) at 27°C in 1/10 liquid MS for 48 h. The expression of eGFP in endophytic bacteria, as either individual cells or intergrowth in the root tips, was followed by confocal microscopy (Zeiss LSM 800) and detected by RT-PCR as a previous publication (Li Q. et al., 2016; Li X. et al., 2016) using the primers described above.

### Transmission Electron Microscopy

Peanut root tips or gynophore tips (see the schematic in Supplementary Figure S1) were fixed in a solution containing 2.5% glutaraldehyde, 0.05 M sodium cacodylate buffer (pH 7.2), and 2% paraformaldehyde by incubation overnight at 4°C. After thorough rinsing in water, root tips were dehydrated and infiltrated in acetone:Epon 812 (1:1) overnight. Thereafter, the root tips were infiltrated with 100% Epon 812 resin for 1 h and embedded in the resin to polymerize. After polymerization, 60–80 nm thin sections of root tips were cut on a Reichert ultramicrotome and stained for 5 min in lead citrate. Sections were rinsed and post-stained for 30 min in uranyl acetate and then rinsed again and dried. Electron microscopy was performed at 60 kV using a Philips Morgagne Transmission Electron Microscopy (TEM) equipped with a CCD. Images were collected at magnifications of 1 000×–37,000× to determine the distribution of endophytic bacteria.

### GC–MS Analysis of Targeted Metabolites and Jasmonate

All root tips were collected in liquid nitrogen then sent to SHANGHAI BIOPROFILE Company, Shanghai, China, for determination of metabolites and JA content by gas chromatography–mass spectrometry (GC–MS). Samples were prepared as follows: 10 g root tips was placed in 1% sulfuric acid–methanol solution (2 mL), blended for 1 min, then methyl esterification was performed for 0.5 h at 80°C in a water bath. Then, 1 mL *n*-hexane was added to the samples for extraction. The samples were washed with 5 mL pure water and 500 μL supernatant was aspirated, to which 100 mg anhydrous sodium sulfate was added to remove excess water. Internal standards (25 μL) were added and mixed well for further analysis. Dihydrojasmonate (DHJA, Sigma) was used as internal standard for determination of JA, while the internal standards for targeted metabolites included 37 fatty acid-mixed methyl esters.

For GC–MS, the extract was injected into an Agilent HP-INNOWAX capillary column (30 m × 0.25 mm × 0.25 μm). Split injections were performed using a 1 μL injection volume and a 10:1 split ratio. The helium flow rate was 1.1 mL/min. The temperature program was as follows: initial column temperature was set at 50°C for 3 min, increased to 220°C at a rate of 10°C/min, and then held for 3 min, and finally increased to 250°C at a rate of 15°C/min and then held until the end of the analysis. The injection and transmission line temperature were set at 250°C. Quantification was performed in the selected-ion monitoring (SIM) mode after electron ionization (70 eV) with a dwell time set at 0.3 s. Source temperature and quadrupole temperature were set at 230 and 150°C, respectively.

### Statistical Analysis

Data were collected and refinement statistics using GraphPad Prism 5. Statistics quantitative data were expressed as mean ± SD of determinations on at least three individual samples. Means were compared using the one-way ANOVA analysis of variance or Student’s *t*-test. Significance was assigned at *P* < 0.01.

## Results

### Isolation of Endophytic Bacteria From Peanut Root Tips

**FIGURE 1:**
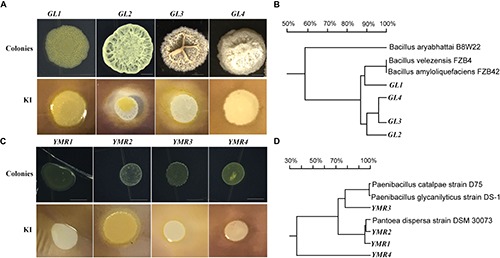
Isolation of endophytic bacteria from root tips. **(A,C)** Colonies and amylolytic capability of B.p.GL1-GL4, P.d.YMR1-YMR2, and P.g.YMR3-YMR4. The bacteria were cultured in LB medium containing 0.15% soluble starch for 24 h. Then drops of 1% KI solution were added and photographs were taken. **(B,D)** 16S DNA variable region sequence relationships of B.p.GL1-GL4, P.d.YMR1-YMR2, and P.g.YMR3-YMR4 determined using DNAMAN software.

Our previous work has shown that similar endophytes are distributed in or around the vacuoles in plant root tissues, to further sustain this, low pH (3.5–5.0) was used to isolate bacteria from the root tips of peanut seedlings grown aseptically or in soil. Forty-two individual colonies were isolated at pH 5.0, while no colonies were obtained at pH 3.5–4.5 (Table 1 and Supplementary Data Sheet S1). The endophytic bacteria were named using the prefix GL to represent those that were isolated from soil-grown peanut seedling root tips, and using the prefix YMR to represent those isolated from aseptic peanut seedling root tips. Most of the endophytic bacteria isolated belong to the genus *Bacillus*. Four *Bacillus* isolates, named *Bacillus* peanut GL1–GL4 (B.p.GL1–GL4), can form a bio-film and have amylase activity (Figure 1).

**TABLE 1:** Summary of endophytes isolated from different peanut root tips.

**Genus**	**Root tip from field soil**	**Root tip from aseptic seedling**
*Bacillus*	13	2
*Kaosakonia*	2	0
*Enterobacter*	2	0
*Klebsiella*	4	0
*Paenibacillus*	3	4
*Staphylococcus*	1	0
*Lactococcus*	1	0
*Burkholderia*	4	0
*Rhizobium*	1	0
*Pantoea*	1	2

Only two *Bacillus* isolates were obtained from the aseptically grown root tips. They shared 99.68 and 96.31% identity in their 16S rDNA to the *B. ginsengihumi* strain Gsoil 114 (*AB245378*), but did not form a bio-film. Two *Pantoea dispersa* isolates, named *P. dispersa* YMR1 (P.d.YMR1) and *P. dispersa* YMR2 (P.d.YMR2), were obtained from aseptically grown root tips. P.d.YMR1 colonies were always touching and very wet, while P.d.YMR2 colonies were round and waxy, with some white spots. The variable regions of the 16S rDNA sequences of P.d.YMR1 and P.d.YMR2 were 91.75% identical (Figure 1). Four *Paenibacillus* sp. isolates were obtained from aseptically grown root tips. Three of these had the same 16S rDNA variable region sequence. The same *Paenibacillus* sp. was also isolated from the root tips grown in soil (Supplementary Data Sheet 1). A third *Paenibacillus* sp. isolate was identified as *Paenibacillus glycanolyticus* and the strain was named YMR3 (P.g.YMR3), while a fourth was named *P. glycanilyticus* YMR4 (P.g.YMR4). However, P.g.YMR3 and P.g.YMR4 only shared 35.32% identity in their 16S rDNA sequences (Figure 1). P.g.YMR3 produced primrose-yellow, translucent, moist, round, and wrinkled colonies, while the colonies of P.g.YMR4 were deep-yellow, round, and smooth. Both types of colony showed strong starch hydrolysis activity (Figure 1).

### Peanut Growth Promotion by Endophytic Bacteria

**FIGURE 2:**
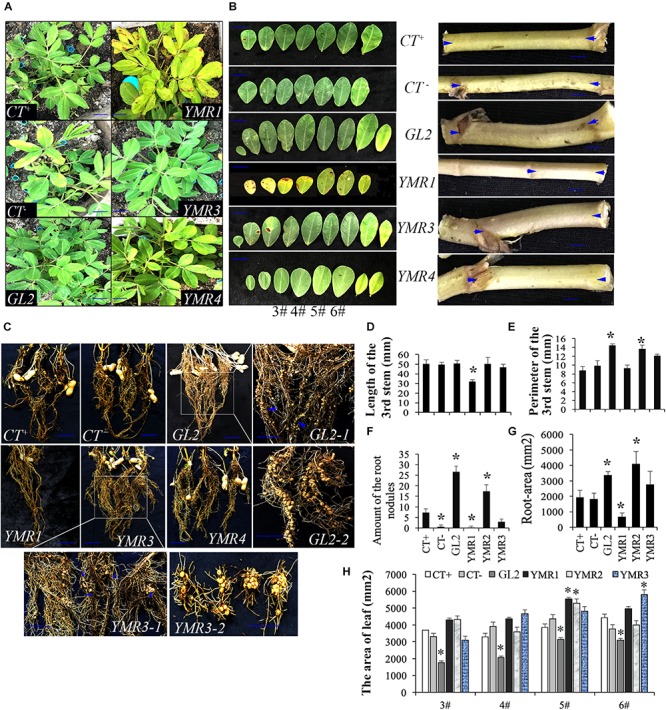
Peanut growth-promoting activity of endophytic bacteria. **(A)** Peanut plants were photographed 20 days after inoculation with B.p.GL2, P.d.YMR1, P.g.YMR3, and P.g.YMR4. **(B)** Representative leaves and shoots of plants in panel **(A)**. **(C)** Root nodules were collected 60 days after inoculation with B.p.GL2, P.g.YMR3, and P.g.YMR4. GL2-1 and GL2-2, and YMR3-1 and YMR3-2 represent magnified images of GL2 and YMR3, respectively. The roots of P.d.YMR1-treated peanuts were measured at days 30–40 due to wilting. **(D–G)** Statistics of stem length **(D)** and perimeter **(E)**, nodules **(F)**, and root area **(G)**. Stem length was measured as the distance between the two arrows shown in panel **(B)** (representative examples). **(H)** Statistics of 3#, 4#, 5#, and 6# leaves, which were new grow up after treatment with endophytic bacteria. All the bars indicate standard deviation in these statistics. “^∗^” indicates significant differences compared with the control (CT^+^) (*P* < 0.01). CT^+^ and CT^–^ represent 2-week old aseptically grown peanut seedling cultivated in soil, which have been sterilized (CT^–^) or not sterilized (CT^+^) for 20 min at 120°C. GL2, YMR1, YMR3, and YMR3 represent B.p.GL2, P.d.YMR1, P.g.YMR3, and P.g.YMR4 bacterial strains, respectively.

The role of endophytes in peanut growth promotion was investigated by inoculating the soil around the roots of peanut seedlings with B.p.GL2, P.d.YMR1, P.g.YMR3, or P.g.YMR4. Both B.p.GL2 and P.g.YMR3 promoted peanut growth and increased plant biomass (Figure 2 and Supplementary Figure S2), and neither strain gave rise to any form of leaf disease or growth retardation. In contrast, inoculation with P.g.YMR4 initially turned peanut leaves yellow, although the leaves then recovered, while P.d.YMR1 caused leaf wilting and induced root rot (Figure 2A). The number of root nodules, root area, leaf area, and the perimeter of the stem were significantly increased in both the B.p.GL2- and P.g.YMR3-inoculated groups, when compared with controls. There were numerous clustered nodules in B.p.GL2-inoculated peanut roots, while large and separate granulated nodules were found in P.g.YMR3-treated peanut roots (Figure 2C). In the P.g.YMR4 or P.d.YMR1 treatment groups, however, no significant increases in nodule numbers were observed (Figures 2D–H). Therefore, we chose to examine the cellular distribution of B.p.GL2 and P.g.YMR3.

### Cellular Distribution of P.g.YMR3

**FIGURE 3:**
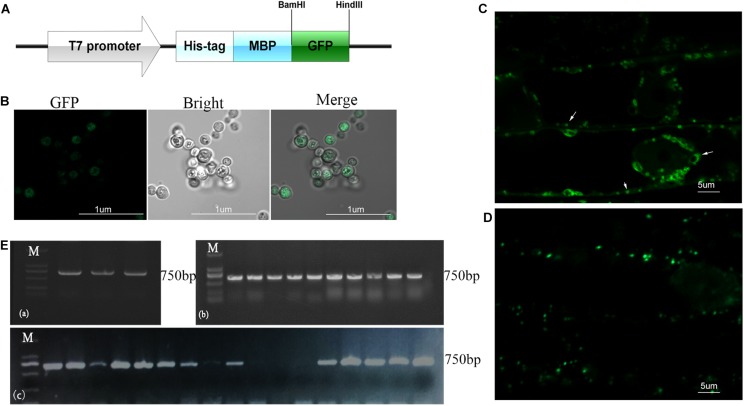
Transmission and distribution of P.g.YMR3. **(A)** Schematic diagram of the green fluorescent protein (GFP) sequence cloned into pRHisMBP. **(B)** The recombinant plasmid was transformed into P.g.YMR3 bacteria by the CaCl_2_ method, induced by 0.5 mM IPTG at 37°C for 6 h, and imaged by confocal microscopy. Green fluorescence represents GFP expression. **(C,D)** The distribution of P.g.YMR3 in peanut roots observed by confocal microscopy. Peanut seedlings treated with P.g.YMR3 and containing GFP plasmid were designated the T0 generation **(C)**, while their seeds were harvested and cultivated as the T1 generation **(D)**. **(E)** GFP transcripts were detected in P.g.YMR3 bacteria after transformation **(a)** and in the T0 **(b)** or T1 **(c)** generations of peanut seedling root tips by RT-PCR.

To investigate the cellular distribution of B.p.GL2 and P.g.YMR3, we attempted to transform recombinant plasmids containing eGFP (Figure 3A) into B.p.GL2 and P.g.YMR3 by the CaCl_2_ or liquid nitrogen freeze-thawing methods. P.g.YMR3 could only be transformed by the CaCl_2_ method, while no positive transformants were observed in B.p.GL2 using either method. After induction by 0.5 mM IPTG at 37°C for 6 h, the green fluorescence of GFP was observed in P.g.YMR3 by confocal microscopy (Figure 3B). The same bacteria were then used to inoculate the roots of peanut seedlings, and subsequently GFP fluorescence was observed around the vacuoles inside root cortical cells (Figure 3C). Interestingly, we also found GFP fluorescence around the vacuoles of root cortical cells in the progeny of P.g.YMR3*-*inoculated plants (Figure 3D). The expression of GFP was confirmed in approximately 93 and 73% root tips of the T1 and T2 generations, respectively, of P.g.YMR3*-*inoculated plants by RT-PCR (Figure 3E).

### P.g.YMR3 Transmission Through Gynophores and Ovules to Peanut Progeny

**FIGURE 4:**
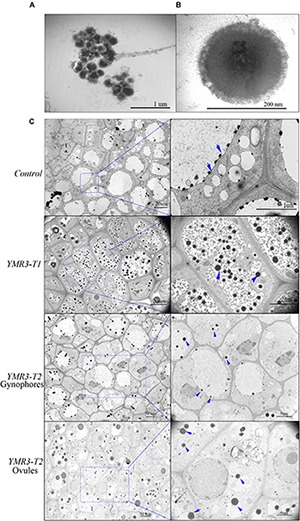
Distribution of endophytic bacteria during peanut development. **(A)** Colonies or **(B)** individual cells of P.g.YMR3 observed by transmission electron microscopy (TEM). **(C)** Endophytic bacteria in peanut root tips. “Control” indicates seedlings grown normally in the greenhouse without treatment with P.g.YMR3 bacteria, while “YMR3-T” represents peanut progeny seedlings had been treated with P.g.YMR3 bacteria. After cultivation for about 45 days, the root tips, gynophores, and ovules were harvested and imaged by TEM. The blue arrows indicate possible individual bacterial cells.

Peanut produces flowers aerially but the fertilized ovules are transported to the roots through the gynophore. However, very little is known about how seed-endophytic bacteria transmit from one generation to the next through the gynophore in peanut. Thus, TEM was used to investigate the distribution of endophytic bacteria in root tips and gynophores of the progeny of P.g.YMR3-inoculated plants. P.g.YMR3 bacteria were detected as dark areas of the images obtained by TEM after staining with osmic acid, and showed them to have thick cell walls (Figures 4A,B). Dark endophytic bacteria were also observed inside the root cortex both in the control and progeny seedlings of YMR3-inoculated peanuts (Figure 4C). Surprisingly, similar endophytic bacteria were also observed in both gynophores and ovules. These endophytic bacteria were distributed mainly around or inside vacuoles (Figure 4C). Furthermore, GFP genes were also detected in the gynophores of the progeny of YMR3-inoculated peanuts by RT-PCR (Supplementary Figure S3). This suggests that the seed endophytic bacterium P.g.YMR3 could be transmitted to the next generation through gynophores and ovules.

### P.g.YMR3 Modulates Peanut Metabolism

**FIGURE 5:**
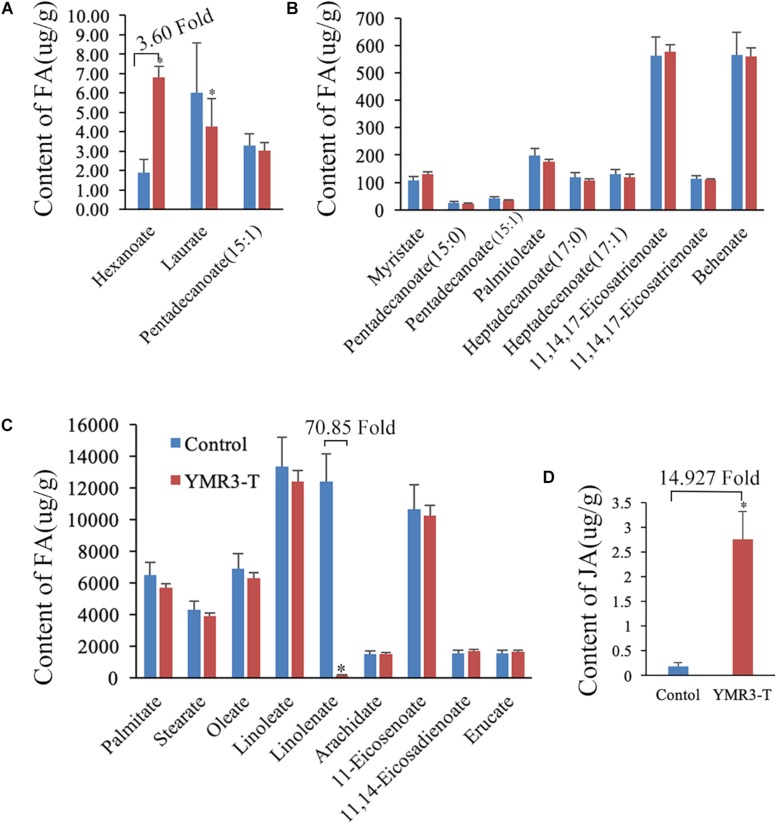
Metabolites in YMR3-infected peanut. **(A–C)** Fatty acid metabolomics was performed using gas chromatography–mass spectrometry (GC–MS). **(D)** Content of jasmonic acid (JA) after inoculation with P.g.YMR3 determined by GC–MS. YMR3-T represents peanut root tips inoculated with P.g.YMR3 bacteria. Three experiments were conducted in each group. All the bars indicate standard deviation in these statistics. “^∗^” indicates significant differences compared with the control (CT^+^) (*P* < 0.01).

Our data show that strain P.g.YMR3 occupies the area in or around vacuoles and is resistant to low pH (5.0); it also promotes peanut growth and is transmitted vertically to the next generation. However, the mechanisms by which it achieves this are unknown. Therefore, the fatty acid and energy metabolomics of peanut were investigated. After inoculation with P.g.YMR3, the contents of hexanoate (3.60-fold change) and succinate (4.25-fold change) were increased in peanut root tips, while the content of linolenate decreased dramatically (70.85-fold change) (Figures 5A–C and Supplementary Table S2). Linolenate is a precursor of JA, which is involved in defense-related processes in various plants (Xie et al., 1998). Accordingly, when we assessed the JA content in peanut root tips after inoculation with P.g.YMR3, we found a marked increase (14.93-fold change) (Figure 5D).

## Discussion

The peanut plant belongs to the legume family, which is associated with nitrogen-fixing bacteria. Most endophytic bacteria isolated from the peanut promote peanut growth. How these endophytes are selected and controlled by peanut, and how the bacteria compete with others and survive inside the peanut tissue remain uninvestigated. Seed-endophytic bacteria can be transmitted from one generation to the next, and thus have huge potential as biocontrol agents and biofertilizers. In this study, 10 genera of endophytic bacteria were isolated at low pH (5.0) from the root tips of peanut seedlings grown aseptically or in soil. Most bacterial isolates belonged to the genus *Bacillus*. Four *Bacillus* peanut strains (B.p.GL1–GL4) are able to form a bio-film. The colonies of the P.d.YMR and P.g.YMR series were smaller than those of B.p.GL1–GL4. Both B.p.GL2 and P.g.YMR3 promoted peanut growth by increasing the number of nodules and plant biomass.

*Bacillus* spp. are known to promote plant growth and are associated with N_2_-fixation in peanut and other plants (Chen et al., 2019). Several *Paenibacillus* spp. were isolated from *Lupinus albus* nodules, indicating that they play a role in nodulation (Carro et al., 2014). P.d.YMR1 and P.g.YMR4 are strains of *P. dispersa*, but little is known about the role of this bacterium in plant growth promotion. Here, we found that neither P.d.YMR1 nor P.g.YMR4 promoted peanut growth, which was surprising given that they were isolated from the same host. One possible explanation for this observation might be that too high a concentration of these bacteria was used for inoculation of peanut roots, and that this exceeded the peanut’s resistance to them. In contrast, both P.g.YMR3 and B.p.GL2 promoted plant growth and increased the number of nodules. To investigate the distribution of these bacteria inside plant tissue and/or cells, we attempted to introduce a GFP marker into B.p.GL2 and P.g.YMR3 prior to inoculation of peanut roots. Because transformation methods have not been established for these bacteria, both CaCl_2_ and liquid nitrogen freeze-thawing methods were performed. Unfortunately, neither method was successful with B.p.GL2, but we were able to transform P.g.YMR3 using the CaCl_2_ method. By following the fluorescence of GFP, we observed that P.g.YMR3 was distributed inside and around vacuoles, which are the central organelle responsible for cellular degradation and nutrient storage in plants. Vacuoles contain hydrolytic enzymes and acts as a “stomach,” digesting and recycling substances including proteins, lipids, organelles, and membrane structures (Suzuki and Emr, 2018; Seaman, 2019). Other authors have shown that endophytic bacteria can be found adhering to the nuclear envelope or vacuoles, as well as the intra-vacuolar space, in banana (Thomas and Sekhar, 2014). In contrast, *Shigella flexneri* enters HeLa cells by internalization into a vacuole, which allows bacterial escape into the cytosol for replication and cell-to-cell transmission (Mellouk et al., 2014). Generally, endophytic microorganisms inhabit the intercellular space, while most P.g.YMR3 bacteria were found around or inside the vacuole according to the GFP marker. Whether they use the same invading pathway as *Shigella* or whether they only obtain some nutrients from vacuoles and exchange signals with peanut cells is unclear. Most endophytic bacteria are only found in root cortical cells, which have clearly developed vacuoles. This is probably why many P.g.YMR3 bacteria were isolated and survived at low pH (5.0). Interestingly, some endophytic bacteria also accumulated in the gynophores and ovules, except at the root tips. GFP expression, indicating the presence of P.g.YMR3, was also detected in second-generation peanut plants derived from P.g.YMR3-inoculated plants. This is strong evidence that the seed-endophytic bacterial strain P.g.YMR3 can be transmitted through the peanut gynophores or ovules to the next generation.

However, how P.g.YMR3 interacts with and is transmitted by peanut still unclear. Strain P.g.YMR3 shows 81.51% identity with *P. glycanilyticus* strain DS-1, which can degrade heteropolysaccharides. Its colonies are flat, smooth, circular, and pinkish yellow (Dasman et al., 2002). P.g.YMR3 can be distinguished from *P. glycanilyticus* strain DS-1 by its colonies, which are primrose yellow, translucent, moist, round, and wrinkle-edged. They have a strong starch hydrolysis capacity and form thick walls, which provide protection and enhance survival and transmission in peanut. Peanut seeds act as reservoirs and vehicles for P.g.YMR3 vertical transmission. Strain P.g.YMR3 also plays an important role in peanut growth and development by modulating peanut metabolism.

Succinate has been widely investigated for its role in the tricarboxylic acid (TCA) cycle as an intermediate metabolite, where it is oxidized to fumarate by succinate dehydrogenase (Bardella et al., 2011; Zhao et al., 2017). After inoculation with P.g.YMR3, succinate content increased by about sixfold in peanut root tips compared with controls. Many studies have focused on the role of succinate beyond metabolism, especially as an inflammatory signal or a carcinogenic initiator in mammals (Zhao et al., 2017). Accumulation of succinate is commonly associated with a number of hereditary and sporadic disorders, such as thyroid cancer, ovarian cancer, gastric cancer, and renal carcinoma (Ricketts et al., 2008; Bardella et al., 2011; Zhao et al., 2017). We propose that succinate accumulation potentially contributes to the formation of nodules in P.g.YMR3-inoculated peanuts. In addition, accumulation of succinate could be a result of the increasing JA content, which is high in P.g.YMR3-inoculated peanuts. The biosynthesis of JA starts with linolenic acid and involves lipoxygenase (LOX), allene oxide synthase, allene oxide cyclase, and β-oxidation (Vick and Zimmerman, 1983; Bell et al., 1995; He et al., 2002; Pauwels et al., 2009; Kausch et al., 2012). LOX is a key enzyme in the oxylipin pathway responsible for generating JA and its derivatives (Pauwels et al., 2009). LOX-silenced tomato fruits have reduced JA and succinate contents (Kausch et al., 2012). Moreover, JA induces succinate production in *Arabidopsis*, *Agastache rugosa*, and *Medicago truncatula* (Broeckling et al., 2005; Gómez et al., 2010; Kim et al., 2013; Bömer et al., 2018). These results suggest that P.g.YMR3-modulated metabolism in peanut may stimulate JA production, which plays different roles in the regulation of plant development and abiotic and biotic stress. In *Arabidopsis*, JA signaling, perceived by the CORONATINE INSENSITIVE 1 (COI1) receptor, mediates pathogen resistance by reducing cellulose biosynthesis, inducing lignin biosynthesis, and promoting the formation of nodules (Caño-Delgado et al., 2003; Mabood et al., 2006; Thines et al., 2007; Denness et al., 2011). Taken together, these results suggest which P.g.YMR3 stimulates peanut to produce JA and to accumulate succinate. Both JA and succinate may contribute to root nodule formation and peanut growth promotion.

## Conclusion

Ten genera of endophytic bacteria were isolated from peanut seedling root tips under low pH (5.0) conditions. Strains B.p.GL2 and P.g.YMR3 promoted peanut growth by increasing plant biomass and the number of root nodules. By labeling with a GFP marker, we showed that strain P.g.YMR3 can be transmitted vertically from one generation to the next via gynophores and ovules. The bacteria were distributed around or inside vacuoles, and stimulated JA production and modulated peanut metabolism.

## Data Availability Statement

The raw data supporting the conclusions of this manuscript will be made available by the authors, without undue reservation, to any qualified researcher.

## Author Contributions

XL and LNL designed this research. LML, ZZ, and SP conducted most of the experimental and data analysis. XL wrote the manuscript. ZZ isolated the endophytic bacteria, whereas LNL performed the promoting, distribution, and transmission of endophytic bacteria. SP detected the metabolism.

## Conflict of Interest

The authors declare that the research was conducted in the absence of any commercial or financial relationships that could be construed as a potential conflict of interest.

## References

[B1] Arguelles-Arias A. Ongena M. Halimi B. Lara Y. Brans A. Joris B. (2009). *Bacillus amyloliquefaciens* GA1 as a source of potent antibiotics and other secondary metabolites for biocontrol of plant pathogens. *Microb. Cell Fac.* 26:63. 10.1186/1475-2859-8-63 19941639 PMC2787494

[B2] Bardella C. Pollard P. J. Tomlinson I. (2011). SDH mutations in cancer. *Biochim. Biophys. Acta.* 1807 1432–1443. 10.1016/j.bbabio.2011.07.003 21771581

[B3] Bell E. Creelman R. A. Mullet J. E. (1995). A chloroplast lipoxygenase is required for wound-induced jasmonic acid accumulation in *Arabidopsis*. *Proc. Natl. Acad. Sci. U.S.A.* 92 8675–8679. 10.1073/pnas.92.19.8675 7567995 PMC41029

[B4] Bömer M. O’Brien J. A. Pérez-Salamó I. Krasauskas J. Finch P. Briones A. (2018). COI1-dependent jasmonate signalling affects growth, metabolite production and cell wall protein composition in *Arabidopsis*. *Ann. Bot.* 122 1117–1129. 10.1093/aob/mcy109 29924303 PMC6324744

[B5] Broeckling C. D. Huhman D. V. Farag M. A. Smith J. T. May G. D. Mendes P. (2005). Metabolic profiling of *Medicago truncatula* cell cultures reveals the effects of biotic and abiotic elicitors on metabolism. *J. Exp. Bot.* 56 323–336. 10.1093/jxb/eri058 15596476

[B6] Caño-Delgado A. Penfield S. Smith C. Catley M. Bevan M. (2003). Reduced cellulose synthesis invokes lignification and defense responses in *Arabidopsis thaliana*. *Plant J.* 34 351–362. 10.1046/j.1365-313x.2003.01729.x 12713541

[B7] Carro L. Flores-Félix J. D. Ramírez-Bahena M. H. García-Fraile P. Martínez-Hidalgo P. Igual J. M. (2014). *Paenibacillus* lupini sp. nov., isolated from nodules of *Lupinus albus*. *Int. J. Syst. Evol. Microbiol.* 64 3028–3033. 10.1099/ijs.0.060830 24928428

[B8] Chen L. Shi H. Heng J. Wang D. Bian K. (2019). Antimicrobial, plant growth-promoting and genomic properties of the peanut endophyte *Bacillus velezensis* LDO2. *Microbiol. Res.* 218 41–48. 10.1016/j.micres.2018.10.002 30454657

[B9] Compant S. Mitter B. Colli-Mull J. G. Gangl H. Sessitsch A. (2011). Endophytes of grapevine flowers, berries, and seeds: identification of cultivable bacteria, comparison with other plant parts, and visualization of niches of colonization. *Microb. Ecol.* 62 188–197. 10.1007/s00248-011-9883-y 21625971

[B10] Dardanelli M. S. González P. S. Bueno M. A. Ghittoni N. E. (2000). Synthesis, accumulation and hydrolysis of trehalose during growth of peanut rhizobia in hyperosmotic media. *J. Basic Microbiol.* 40 149–156. 10957956 10.1002/1521-4028(200007)40:3<149::AID-JOBM149>3.0.CO;2-Y

[B11] Dasman P. A. Kajiyama S. Kawasaki H. Yagi M. Seki T. Fukusaki E. (2002). *Paenibacillus glycanilyticus* sp. nov., a novel species that degrades heteropolysaccharide produced by the cyanobacterium *Nostoc commune*. *Int. J. Syst. Evol. Microbiol.* 52 1669–1674. 10.1099/00207713-52-5-1669 12361272

[B12] DeLong E. F. (1992). Archaea in coastal marine environments. *Proc. Natl. Acad. Sci. U.S.A.* 89 5685–5689. 10.1073/pnas.89.12.5685 1608980 PMC49357

[B13] Denness L. McKenna J. F. Segonzac C. Wormit A. Madhou P. Bennett M. (2011). Cell wall damage-induced lignin biosynthesis is regulated by a reactive oxygen species- and jasmonic acid-dependent process in *Arabidopsis*. *Plant Physiol.* 156 1364–1374. 10.1104/pp.111.175737 21546454 PMC3135913

[B14] Fincheira P. Quiroz A. (2018). Microbial volatiles as plant growth inducers. *Microbiol Res.* 208 63–75. 10.1016/j.micres.2018.01.002 29551213

[B15] Gómez S. Ferrieri R. A. Schueller M. Orians C. M. (2010). Methyl jasmonate elicits rapid changes in carbon and nitrogen dynamics in tomato. *New Phytol.* 188 835–844. 10.1111/j.1469-8137.2010.03414.x 20723074

[B16] Haggag W. M. Timmusk S. (2008). Colonization of peanut roots by biofilm-forming *Paenibacillus polymyxa* initiates biocontrol against crown rot disease. *J. Appl. Microbiol.* 104 961–969. 10.1111/j.1365-2672.2007.03611.x 18005030

[B17] He Y. Fukushige H. Hildebrand D. F. Gan S. (2002). Evidence supporting a role of jasmonic acid in *Arabidopsis* leaf senescence. *Plant Physiol.* 128 876–884. 10.1104/pp.010843 11891244 PMC152201

[B18] Huang S. Pang F. (2017). Biocontrol agents for controlling wheat rust. *Methods Mol. Biol.* 1659 277–288. 10.1007/978-1-4939-7249-4-24 28856659

[B19] Jiang G. Wei Z. Xu J. Chen H. Zhang Y. She X. (2017). Bacterial wilt in China: history, current status, and future perspectives. *Front. Plant Sci.* 8:1549. 10.3389/fpls.2017.01549 28955350 PMC5601990

[B20] Johnston-Monje D. Raizada M. N. (2011). Conservation and diversity of seed associated endophytes in *Zea* across boundaries of evolution, ethnography and ecology. *PLoS One* 6:e20396. 10.1371/journal.pone.0020396 21673982 PMC3108599

[B21] Kausch K. D. Sobolev A. P. Goyal R. K. Fatima T. Laila-Beevi R. Saftner R. A. (2012). Methyl jasmonate deficiency alters cellular metabolome, including the aminome of tomato (*Solanum lycopersicum* L.) fruit. *Amino Acids* 42 843–856. 10.1007/s00726-011-1000-5 21814797

[B22] Kim Y. B. Kim J. K. Uddin M. R. Xu H. Park W. T. Tuan P. A. (2013). Metabolomics analysis and biosynthesis of rosmarinic acid in *Agastache rugosa* Kuntze treated with methyl jasmonate. *PLoS One* 8:e64199. 10.1371/journal.pone.0064199 23724034 PMC3665811

[B23] Li Q. Lei S. Du K. Li L. Pang X. Wang Z. (2016). RNA-seq based transcriptomic analysis uncovers α-linolenic acid and jasmonic acid biosynthesis pathways respond to cold acclimation in *Camellia japonica*. *Sci. Rep.* 6:36463. 10.1038/srep36463 27819341 PMC5098223

[B24] Li X. Li X. Li M. Yan Y. Liu X. Li L. (2016). Dual function of NAC072 in ABF3-mediated ABA-responsive gene regulation in *Arabidopsis*. *Front. Plant Sci.* 2016:1075. 10.3389/fpls.2016.01075 27486475 PMC4949229

[B25] Liu D. Yang Q. Ge K. Hu X. Qi G. Du B. (2017). Promotion of iron nutrition and growth on peanut by *Paenibacillus illinoisensis* and *Bacillus* sp. strains in calcareous soil. Braz. *J. Microbiol.* 48 656–670. 10.1016/j.bjm.2017.02.006 28645648 PMC5628301

[B26] Ludueña L. M. Anzuay M. S. Angelini J. G. McIntosh M. Becker A. Rupp O. (2018). Genome sequence of the endophytic strain *Enterobacter* sp. J49, a potential biofertilizer for peanut and maize. *Genomics* 111 913–920. 10.1016/j.ygeno.2018.05.021 29857118

[B27] Mabood F. Souleimanov A. Khan W. Smith D. L. (2006). Jasmonates induce nod factor production by *Bradyrhizobium japonicum*. *Plant. Physiol. Biochem.* 44 759–765. 10.1016/j.plaphy.2006.10.025 17107814

[B28] Madhaiyan M. Suresh Reddy B. V. Anandham R. Senthilkumar M. Poonguzhali S. Sundaram S. P. (2006). Plant growth-promoting *Methylobacterium* induces defense responses in groundnut (*Arachis hypogaea* L.) compared with rot pathogens. *Curr. Microbiol.* 53 270–276. 10.1007/s00284-005-0452-9 16941245

[B29] Mano H. Tanaka F. Watanabe A. Kaga H. Okunishi S. Morisaki H. (2006). Culturable surface and endophytic bacterial flora of the maturing seeds of rice plants (*Oryza sativa*) cultivated in a paddy field. *Microbes Environ.* 21 86–100. 10.1264/jsme2.21.86

[B30] Mellouk N. Weiner A. Aulner N. Schmitt C. Elbaum M. Shorte S. L. (2014). *Shigella* subverts the host recycling compartment to rupture its vacuole. *Cell Host Microbe* 16 517–530. 10.1016/j.chom.2014.09.005 25299335

[B31] Pauwels L. Inzé D. Goossens A. (2009). Jasmonate-inducible gene: what does it mean? *Trends Plant Sci.* 14 87–91. 10.1016/j.tplants.2008.11.005 19162528

[B32] Prestes F. S. Pereira A. A. M. Silva A. C. M. Pena P. O. Nascimento M. S. (2019). Effects of peanut drying and blanching on *Salmonella* spp. *Food Res. Int.* 119 411–416. 10.1016/j.foodres.2019.02.017 30884671

[B33] Ricketts C. Woodward E. R. Killick P. Morris M. R. Astuti D. Latif F. (2008). Germline SDHB mutations and familial renal cell carcinoma. *J. Natl. Canc. Inst.* 100 1260–1262. 10.1093/jnci/djn254 18728283

[B34] Rojo F. G. Reynoso M. M. Ferez M. Chulze S. N. Torres A. M. (2007). Biological control by *Trichoderma* species of *Fusarium solani* causing peanut brown root rot under field conditions. *Crop Protect.* 26 549–555. 10.1016/j.cropro.2006.05.006

[B35] Sadaf K. Tushar L. Nirosha P. Podile A. R. Sasikala Ch Ramana C. h. V. (2016). *Paenibacillus arachidis* sp. nov., isolated from groundnut seeds. *Int. J. Syst. Evol. Microbiol.* 66 2923–2928. 10.1099/ijsem.0.001124 27129367

[B36] Seaman M. (2019). Back from the brink: retrieval of membrane proteins from terminal compartments: unexpected pathways for membrane protein retrieval from vacuoles and endolysosomes. *Bioessays* 41:e1800146. 10.1002/bies.201800146 30706963

[B37] Shahzad R. Khan A. L. Bilal S. Asaf S. Lee I. J. (2018). What is there in seeds? vertically transmitted endophytic resources for sustainable improvement in plant growth. *Front. Plant Sci.* 9:24. 10.3389/fpls.2018.00024 29410675 PMC5787091

[B38] Sobolev V. Arias R. Goodman K. Walk T. Orner V. Faustinelli P. (2018). Suppression of aflatoxin production in *Aspergillus* species by selected peanut (*Arachis hypogaea*) stilbenoids. *J. Agric. Food. Chem.* 66 118–126. 10.1021/acs.jafc.7b04542 29207242

[B39] Sobolev V. S. Orner V. A. Arias R. S. (2013). Distribution of bacterial endophytes in peanut seeds obtained from axenic and control plant material under field conditions. *Plant Soil.* 371 367–376. 10.1007/s11104-013-1692

[B40] Sundin G. W. Jacobs J. L. (1999). Ultraviolet radiation (UVR) sensitivity analysis and UVR survival strategies of a bacterial community from the Phyllosphere of field-grown Peanut (*Arachis hypogeae* L.). *Microb. Ecol.* 38 27–38. 10.1007/s002489900152 10384007

[B41] Suzuki S. W. Emr S. D. (2018). Membrane protein recycling from the vacuole/lysosome membrane. *J. Cell Biol.* 217 1623–1632. 10.1083/jcb.201709162 29511122 PMC5940307

[B42] Thines B. Katsir L. Melotto M. Niu Y. Mandaokar A. Liu G. (2007). JAZ repressor proteins are targets of the SCFCOI1 complex during jasmonate signalling. *Nature* 448 661–665. 10.1038/nature05960 17637677

[B43] Thomas P. Sekhar A. C. (2014). Live cell imaging reveals extensive intracellular cytoplasmic colonization of banana by normally non-cultivable endophytic bacteria. *AoB Plants* 6:lu002. 10.1093/aobpla/plu002 24790123 PMC4038436

[B44] Truyens S. Weyens N. Cuypers A. Vangronsveld J. (2014). Bacterial seed endophytes: genera, vertical transmission and interaction with plants. *Environ. Microbiol. Rep.* 7 40–50.

[B45] Vargas Gil S. Meriles J. M. Haro R. Casini C. March G. J. (2008). Crop rotation and tillage systems as a proactive strategy in the control of peanut fungal soilborne diseases. *BioControl* 53 685–698. 10.1007/s10526-007-9105-9101

[B46] Vick B. A. Zimmerman D. C. (1983). The biosynthesis of jasmonic acid: a physiological role for plant lipoxygenase. *Biochem. Biophys. Res. Commun.* 111 470–477. 10.1016/0006-291X(83)90330-3 6404266

[B47] Wang X. Liang G. (2014). Control efficacy of an endophytic *Bacillus amyloliquefaciens* strain BZ6-1 against peanut bacterial wilt, *Ralstonia solanacearum*. *Biomed. Res. Int.* 2014:465435. 10.1155/2014/465435 24527448 PMC3912762

[B48] Xie D. X. Feys B. F. James S. Nieto-Rostro M. Turner J. G. (1998). COI1: an *Arabidopsis* gene required for jasmonate-regulated defense and fertility. *Science* 280 1091–1094. 9582125 10.1126/science.280.5366.1091

[B49] Zhao T. Mu X. You Q. (2017). Succinate: an initiator in tumorigenesis and progression. *Oncotarget* 8 53819–53828. 10.18632/oncotarget.17734 28881853 PMC5581152

[B50] Zhou X. Zhang N. Xia L. Li Q. Shao J. Shen Q. (2018). ResDE two-component regulatory system mediates oxygen limitation-induced biofilm formation by *Bacillus amyloliquefaciens* SQR9. *Appl. Environ. Microbiol.* 84:e2744-17. 10.1128/AEM.02744-17 29427424 PMC5881065

