# Supplementary material for: Characterization and Metabolism Effect of Seed Endophytic Bacteria Associated With Peanut Grown in South China
Source: Front Microbiol. 2019 Nov 13;10:2659. doi: 10.3389/fmicb.2019.02659 (PMC6865467; doi:10.3389/fmicb.2019.02659)
Supplement: Supplementary file 1 [file Table_1.DOCX]

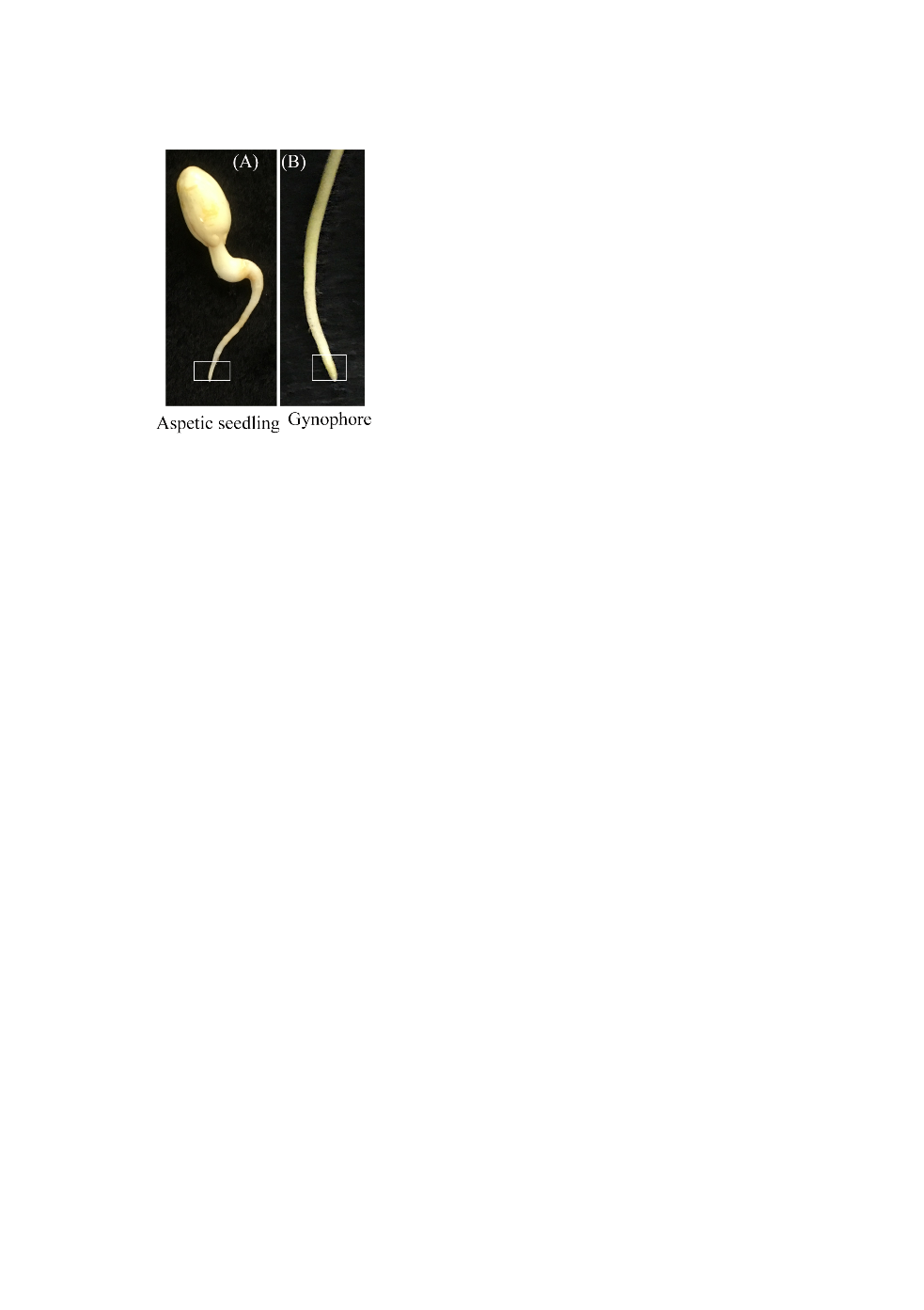


**Figure S1 schematic of root tip or gynophore tip**

(A), the rectangle is shown the soli or aseptic root tip, which were mashed and to isolated endopytic bacterium, or observed the endophytic bacterium distribution under confocal microscopy or TEM in this study. (B), the rectangle is shown the gynophore tip, which used to observed the endophytic bacterium distribution under TEM and detected the GFP expressed.


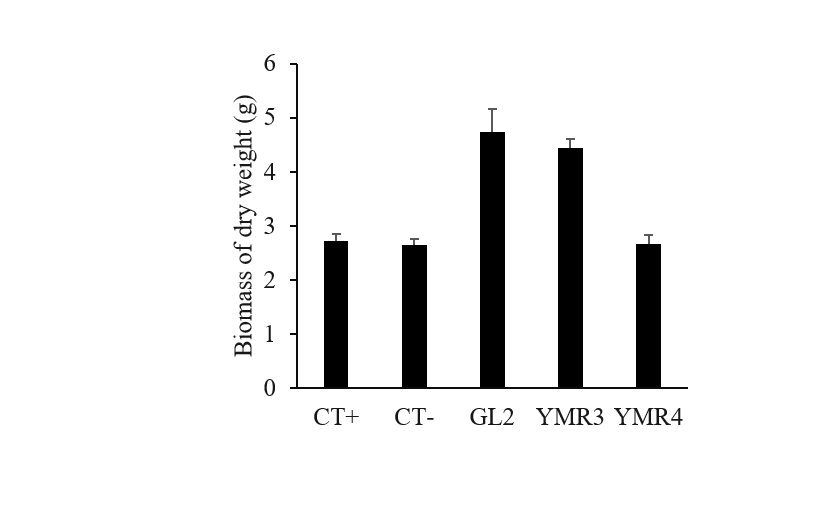


**Figure S2 the biomass of peanut after treatment**

Peanut were inoculated with *B.p.GL2, P.d.YMR1, P.g.YMR3* or *P.g.YMR4*for 60 days, respectively. Then the dry biomass were measured. The *P.d.YMR1* bacterium treatment caused peanut wilt and the biomass could not measure after 60 days.


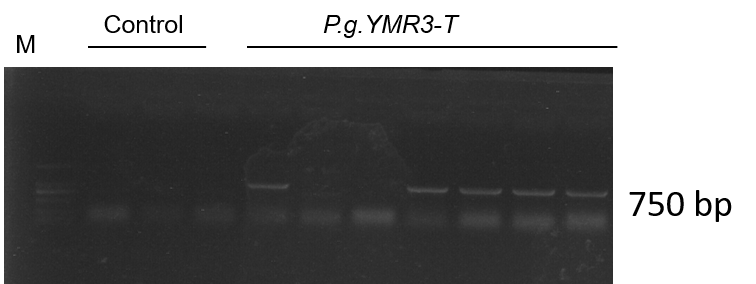


**Figure S3 detected the GFP fragment in *P.g.YMR3*-incobulated peanut offspring**

The DNA were extracted in the gynophores from offspring of *P.g.YMR3*-incobulated peanut or control. Then the GFP fragment were conducted to detect by RT-PCR.
