# Supplementary material for: Characterization and Metabolism Effect of Seed Endophytic Bacteria Associated With Peanut Grown in South China
Source: Front Microbiol. 2019 Nov 13;10:2659. doi: 10.3389/fmicb.2019.02659 (PMC6865467; doi:10.3389/fmicb.2019.02659)
Supplement: Supplementary file 3 [file Data_Sheet_1.PDF]

Sequences

Pantoea dispersa YMR1

GCAAGCGGCAGACTACACATGCAGTCGAACGGCAGCACAGAAGAGCTTGCTCTTTGGGTGGCGAGTG
GCGGACGGGTGAGTAATGTCTGGGAAACTGCCCGATGGAGGGGGATAACTACTGGAAACGGTAGCTA
ATACCGCATAACGTCGCAAGACCAAAGTGGGGGACCTTCGGGCCTCACACCATCGGATGTGCCCAGAT
GGGATTAGCTAGTAGGTGGGGTAATGGCTCACCTAGGCGACGATCCCTAGCTGGTCTGAGAGGATGAC
CAGCCACACTGGAACTGAGACACGGTCCAGACTCCTACGGGAGGCAGCAGTGGGGAATATTGCACAA
TGGGCGCAAGCCTGATGCAGCCATGCCGCGTGTATGAAGAAGGCCTTCGGGTTGTAAAGTACTTTCAG
CGGGGAGGAAGGCGGTGAGGTTAATAACCTTGCCGATTGACGTTACCCGCAGAAGAAGCACCGGCTA
ACTCCGTGCCAGCAGCCGCGGTAATACGGAGGGTGCAAGCGTTAATCGGAATTACTGGGCGTAAAGC
GCACGCAGGCGGTCTGTTAAGTCAGATGTGAAATCCCCGGGCTTAACCTGGGAACTGCATTTGAAACT
GGCAGGCTTGAGTCTCGTAGAGGGGGGTAGAATTCCAGGTGTAGCGGTGAAATGCGTAGAGATCTGG
AGGAATACCGGTGGCGAAGGCGGCCCCCTGGACGAAGACTGACGCTCAGGTGCGAAAGCGTGGGGA
GCAAACAGGATTAGATACCCTGGTAGTCCACGCCGTACACGATGTCGACTTGGAGGTTGTGCCCTTGA
GGCGTGGCTTCCGGAGCTAACGCGTTAAGTCGACCGCCTGGGGAGTACGGCCGCAAGGTTAAAACTC
AAATGAATTGACGGGGGCCCGCACAAGCGGTGGAGCATGTGGTTTAATTCGATGCAACGCGAGAACC
TTACCTGGCCCTTGACATCCAGAGAACTTAGCAGAGATGCTCTGGCTGCCTTCGGGAACTCTGAGAAG
TGGCCTTGCCTGGGAATGGTCGTCAGCCTCGTGTTTGTGAATGTTTGGGTAGTCCCGCACGAGCGCAC
CTATCTTGTGCAGCGCTCGGCGGGACTCAAGGAGACTGCGTGAATAACGGAAGAGTTGGGATGACGT
CCAGGTCATTCATGGCCTTACGGCAGTCTTAACAACGTGCTACAAATGGGCGCCCTATATCAAGAGAG
AAC

Pantoea dispersa strain DSM 30073 16S ribosomal RNA, partial sequence	1792	1792	97%	0.0	94.22%	NR_116797.1
Pantoea dispersa strain LMG 2603 16S ribosomal RNA, partial sequence	1719	1719	92%	0.0	94.67%	NR_116755.1
Pantoea dispersa strain LMG 2603 16S ribosomal RNA, partial sequence	1718	1718	92%	0.0	94.58%	NR_043883.1
Kluyvera cryocrescens strain 12993 16S ribosomal RNA, partial sequence	1712	1712	97%	0.0	92.90%	NR_028803.1
Kluyvera cryocrescens strain NBRC 102467 16S ribosomal RNA, partial sequence	1709	1709	97%	0.0	92.82%	NR_114108.1
Pantoea stewartii strain LMG 2715 16S ribosomal RNA, partial sequence	1705	1705	97%	0.0	92.66%	NR_119361.1
Kluyvera intermedia strain 256 16S ribosomal RNA, partial sequence	1702	1702	96%	0.0	93.00%	NR_028802.1
Pantoea stewartii subsp. indologenes strain CIP 104006 16S ribosomal RNA, partial sequence	1701	1701	97%	0.0	92.66%	NR_104928.1
Kluyvera intermedia strain JCM1238 16S ribosomal RNA, partial sequence	1699	1699	97%	0.0	92.66%	NR_112007.1
Pantoea stewartii strain ATCC 8199 16S ribosomal RNA, partial sequence	1697	1697	96%	0.0	92.92%	NR_044800.1

Pantoea dispersa YMR2

GCTTGGCGGCAGACTACACATGCAGTCGAACGGCAGCACAGAAGAGCTTGCTCTTTGGGTGGCGAGT
GGCGGACGGGTGAGTAATGTCTGGGAAACTGCCCGATGGAGGGGGATAACTACTGGAAACGGTAGCT
AATACCGCATAACGTCGCAAGACCAAAGTGGGGGACCTTCGGGCCTCACACCATCGGATGTGCCCAG
ATGGGATTAGCTAGTAGGTGGGGTAATGGCTCACCTAGGCGACGATCCCTAGCTGGTCTGAGAGGATG
ACCAGCCACACTGGAACTGAGACACGGTCCAGACTCCTACGGGAGGCAGCAGTGGGGAATATTGCAC
AATGGGCGCAAGCCTGATGCAGCCATGCCGCGTGTATGAAGAAGGCCTTCGGGTTGTAAAGTACTTTC
AGCGGGGAGGAAGGCGGTGAGGTTAATAACCTTGCCGATTGACGTTACCCGCAGAAGAAGCACCGGC
TAACTCCGTGCCAGCAGCCGCGGTAATACGGAGGGTGCAAGCGTTAATCGGAATTACTGGGCGTAAAG
CGCACGCAGGCGGTCTGTTAAGTCAGATGTGAAATCCCCGGGCTTAACCTGGGAACTGCATTTGAAAC
TGGCAGGCTTGAGTCTCGTAGAGGGGGGTAGAATTCCAGGTGTAGCGGTGAAATGCGTAGAGATCTG
GAGGAATACCGGTGGCGAAGGCGGCCCCCTGGACGAAGACTGACGCTCAGGTGCGAAAGCGTGGGG
AGCAAACAGGATTAGATACCCTGGTAGTCCACGCCGTAAACGATGTCGACTTGGAGGTTGTGCCCTTG
AGGCGTGGCTTCCGGAGCTAACGCGTTAAGTCGACCGCCTGGGGAGTACGGCCGCAAGGTTAAAACT
CAAATGAATTGACGGGGGCCCGCACAAGCGGTGGAGCATGTGGTTAATTCGATGCAACGCGAAGAAC
CTTACCTGGCCTTGACATCCATAGAACTTAGCAGAGATGCTTTGGTGCCTTCGGCACTCTGAGACAGA
GGCTGCATCGTACTGTCGTCAGCTCGTGTGTGAAATGTTGGGTAAGTCCCGCACGAGCGCACCCTATC

TTGTGCAGCGCTCGGCGGACTCAAAGGAACTGCGTGATAACGAGGAGGTGGGATGACGTCAGTCATC
ATGCTTACGGCCAGGCTAACACATGTCCAATGCCACAGGACGACTCCAGAACAGC
GAACCTCATAAGTGCGCTC

Pantoea dispersa strain DSM 30073 16S ribosomal RNA gene, partial sequence	1919	1919	96%	0.0	95.86%	NR_116797.1
Pantoea dispersa strain LMG 2603 16S ribosomal RNA gene, partial sequence	1842	1842	90%	0.0	96.31%	NR_116755.1
Pantoea dispersa strain LMG 2603 16S ribosomal RNA gene, partial sequence	1838	1838	90%	0.0	96.22%	NR_043883.1
Kluyvera cryocrescens strain 12993 16S ribosomal RNA gene, partial sequence	1814	1814	93%	0.0	95.05%	NR_028803.1
Kluyvera cryocrescens strain NBRC 102467 16S ribosomal RNA gene, partial sequence	1812	1812	93%	0.0	94.97%	NR_114108.1
Pantoea cypripedii strain DSM 3873 16S ribosomal RNA gene, partial sequence	1808	1808	96%	0.0	94.21%	NR_041973.1
Erwinia endophytica strain BSTT30 16S ribosomal RNA, partial sequence	1801	1801	96%	0.0	94.12%	NR_148650.1
Pantoea wallisii strain LMG 26277 16S ribosomal RNA, partial sequence	1801	1801	90%	0.0	95.69%	NR_118122.1
Pantoea cypripedii strain ATCC 29267 16S ribosomal RNA gene, partial sequence	1799	1799	94%	0.0	94.59%	NR_118857.1
Pantoea cypripedii strain LMG 2657 16S ribosomal RNA gene, partial sequence	1797	1797	96%	0.0	93.79%	NR_118394.1

Peaenibacillus glycanliyticus YMR3

(Three monoclonal are the same as this one from the aseptic or soil seedling root tip)
GCGAATGGGGGGACTACACATGCAGTCGAGCGGATCTTTTCCTTCCGTGAGAGGTTAGCGGCGGACG
GGTGGGTAAGACAGGAATGACCGGGACACTACACGGGGGTAACATTCGGAAACGGATGCCGGTCCCG
GATACCCCAATTGGTCGCATGACCAAATCGGGAAACGATGAGCCCTCTGCCCCTTATGGGTGGCCCCG
CGGCGCATTATCTTGTGGGGGAGGTAGGGGCTCATCAGGGACACTATGCGTATGCGACCTGAGGAGGT
GATCGGCCACTGTGGGTCTGACACACGGCCCACACTCCTACGGGGGGCAACTGTAGGAAATCTTCCG
CTGTGGACGAGCGTGTGACGGACCTGCCCCGTGTGTGTGATGAGGGTTTTCGGATCATAGAGCTCTGT
TGCCGGGGAAGAGCGCTTACGAGAGTACCTGCTCTTAATGAGACGGCACCTGAAAAAAAAGCCCCGG
CTAACTACGTGCCAACACCCGCGGTTATACATAGGGGGCAAGCTTTGTCCAGAATTAGTGGGTATAGAG
CGCGCACGGGCTGCCTTGTAACTCTGTCGTTTCACTCGGATCTCACCTTCAACTCGCGATGGAAACTG
CAAACCTTGATTGCAGAAAAGGAAAGTGAAATTCCGCGTGTAGCGGGAAAATGCTTAAAGATGTGGA
GGAACACCGGTGGCGAAAGGCGACTTTCTGGGCTGTACTGACGCTGAGGAGCGAAAGCGTGGGGGA
GCAAACAGGATTAGATACCCTGGTAGTCCACGCCGTAAACTATGTAATGCTGGGATGTTGTGCGCTTCG
AGACCCGTTGTTGCCGAAAGTTAACGCATTAAATCGATCCCGCCGGGGGGAGTACGGTCGCAAGTACT
GACACTCATAAGAATTTCACGGGGACCGCGCACAAGCAGTGGAGTATGTCGTTTAAATTCGTAACAAG
CGTAAAAAGCCTATACCGGGCTCTTAGAACTCCCTTCAGATCTGTCCCTAGATGACAGTGCTTCCCTTC
GGGAACAGAGGACACAGCGGTGCCATGCCTTGCCGTCACTCCGTGTCTGAAATGTCGCTAAGTCCGC
AACGAGCGTACCCTATGCTCTTATTGCAAGCCCTTAGCGTGAACCTCAAGCATATGCCAGGTAACAATC
GGAGACGGTGGATGACTCGAATCCTAGCCTTCGAACTTGTACCCGTTCCTCAATTGGCCGAATGCAAA
CAG

Paenibacillus glycanilyticus strain DS-1 16S ribosomal RNA gene, partial sequence	922	922	94%	0.0	81.51%	NR_024759.1
Paenibacillus xinjiangensis strain B538 16S ribosomal RNA gene, partial sequence	841	841	92%	0.0	80.54%	NR_043221.1
Paenibacillus castaneae strain Ch-32 16S ribosomal RNA, partial sequence	832	832	91%	0.0	80.56%	NR_044403.1
Paenibacillus kobensis strain DSM 10249 16S ribosomal RNA gene, partial sequence	780	780	87%	0.0	80.28%	NR_040894.1
Paenibacillus kobensis strain IFO15729 16S ribosomal RNA, partial sequence	778	778	87%	0.0	80.26%	NR_115598.1
Paenibacillus agaridevorans strain DSM 1355 16S ribosomal RNA gene, partial sequence	763	763	86%	0.0	80.22%	NR_025490.1
Paenibacillus mendelii strain C/2 16S ribosomal RNA gene, partial sequence	760	760	86%	0.0	80.07%	NR_041929.1
Paenibacillus harenae strain B519 16S ribosomal RNA gene, partial sequence	756	756	94%	0.0	79.01%	NR_043220.1
Paenibacillus phyllosphaerae strain PALXIL04 16S ribosomal RNA, partial sequence	754	754	90%	0.0	79.57%	NR_043008.1
Paenibacillus agarexedens strain DSM 1327 16S ribosomal RNA gene, partial sequence	752	752	89%	0.0	79.57%	NR_025489.1

Peaenibacillus glycanliyticus YMR4

CCTATTTTACCACCTTCGAAAGCTGGCTCCTTGCGTTGATACCCCACCGGTTTCGGGTGTCCAAAACTC
TGGTGGTGCGGCGGGCGGTGTGTACGACACCCGGGAACTTATTCACCCCGGCATGCTGATCCGCGATT

ACTAGCAATTCCGACTTCATGCAGGAGAGTTGCAGCCTGCAATCCAAACTGAGACCGACTTTGATAGG
ATTGGCTCCTCCTCGGGGTTTCTCTTCCCGTTGTATCCATTGTTGTCGTGGTGTGCCCCCCAGGTCATGG
GGGGGATGATGATTTGACGTCCCCCCCTTCTTCCTCCGGTTTGTCGGCGGTCGTCTTCGTATTCCGCCC
CCCCAAGGTGGTGGCAAATGAGAAGGGGGGTTGCGCTCGTGGCGGTACTTAACCCATCTTCTCACAA
CACGAGATGACGACAACGATGCACCTGTTGTCTCCTCTGTCAAGGAAGAAACCCCTATCTCAAGGATT
CTCAGAATGATGTCAAGACGTGAGGAGGTTCTCCTTGTTTCTTATAATTAAACCTCATCCTCCGCTGGT
GGGGGGCCCCCCCATTTATTTTTTTATTTTCCTTCTTGCCACCCTCCCCCCGAGGCGGAATGCATAATG
TATTTACTTCAGCACCGAGGGTATCGCACCCCCTCACACCTAACATTCTTCGTTGACGGCGTGAACTAC
GAAGGTATCTAATCTTGTTCGCTCCCCTTTCTTTCGCGCCTCAGCGTCAGCTACAGCCCAGAAAGTCTC
CCTTCGCCACTGGTGTTCCTATATATCTCTATTTATTTCATCGCTACGCAAGTAAATCCCACTTTCTCCTC
ATCTGCACTCAAGCTATGATGATACGATCGTGACTCGAAGATTCACCGTGGATTTTTAATCTAACATTAC
TTAAAACGGCCTGCCTGCGCGCTTTTTTACCACCAATAACTTACTAGAACAATTGCTCGCCCTCCTTAC
TTATTACCGGCTGGCTGGCTAGGCAAGTTAAGTTCAGGCGCGTGCTTTCTTTCCTGAAAGCACCGACG
ACCCTTACGATCATTATTACTTCTCGATCATGTCGCTTCTCTCGCCTGGAAAGATAGTTTCATTTAACCG
AATACGGAACAGTTCTTTCATACACTTCAGGCAGGGTGCATCAGAGCAGTAGCTTCGATCGATGGCAG
ATGTATCTCCACCTGACTGCGTGCCTTCCAGAGGCAGGGCTACGGGGCGTGTGTCACTACCACTTCGA
AGTTGCTGACTGAATTCATCTCTGAACAAGAGAACTAAAGCGA

Select: All None Selected:0

Alignments Download GenBank Graphics Distance tree of results

	Description	Max Score	Total Score	Query Cover	E value	Per. Ident	Accession
	Paenibacillus glycanilyticus strain DS-1 16S ribosomal RNA, partial sequence	835	835	76%	0.0	80.08%	NR_024759.1
	Paenibacillus catalpae strain D75 16S ribosomal RNA, partial sequence	831	831	76%	0.0	80.08%	NR_118012.1
	Paenibacillus glycanilyticus strain NBRC 16618 16S ribosomal RNA, partial sequence	829	829	76%	0.0	79.98%	NR_113853.2
	Paenibacillus gorillae strain G1 16S ribosomal RNA, partial sequence	822	822	76%	0.0	79.88%	NR_136879.1
	Paenibacillus radicis strain 694 16S ribosomal RNA, partial sequence	815	815	75%	0.0	79.80%	NR_148658.1
	Paenibacillus xinjiangensis strain B538 16S ribosomal RNA, partial sequence	809	809	76%	0.0	79.57%	NR_043221.1
	Paenibacillus castaneae strain Ch-32 16S ribosomal RNA, partial sequence	801	801	76%	0.0	79.49%	NR_044403.1
	Paenibacillus lupini strain RLAHU15 16S ribosomal RNA, partial sequence	786	786	76%	0.0	79.53%	NR_134115.1
	Paenibacillus endophyticus strain PECAE04 16S ribosomal RNA, partial sequence	783	783	76%	0.0	79.31%	NR_135705.1
	Paenibacillus prosopidis strain PW21 16S ribosomal RNA, partial sequence	779	779	76%	0.0	78.98%	NR_116828.1
	Paenibacillus tarimensis strain SA-7-6 16S ribosomal RNA, partial sequence	774	774	76%	0.0	78.56%	NR_044102.1

Bacillus ginsengihumi YMR6

GGGCGTGCGGGTGCTATACATGCAGTCGAGCGAACTGATGAAGAGCTTGCTTTTGATCAGTTAGCGGC
GGACGGGTGAGTAACACGTGGGTAACCTGCCTGTAAGACTAGGATAACTCCGGGAAACCGGGGCTAA
TACTGGATAACTTTTCTCTCCGCATGGAGGGAGATTGAAAGATGGCTTCGGCTATCACTTACAGATGGA
CCCGCGGCGCATTAGCTAGTTGGTGAGGTAACGGCTCACCAAGGCAACGATGCGTAGCCGACCTGAG
AGGGTGATCGGCCACATTGGGACTGAGACACGGCCCAAACTCCTACGGGAGGCAGCAGTAGGGAATC
TTCCGCAATGGACGAAAGTCTGACGGAGCAACGCCGCGTGAGTGAAGAAGGTCTTCGGATCGTAAAA
CTCTGTTGTTAGGGAAGAACAAGTATCGTTCGAATAGGGCGGTACCTTGACGGTACCTAACCAGAAAG
CCACGGCTAACTACGTGCCAGCAGCCGCGGTAATACGTAGGTGGCAAGCGTTGTCCGGAATTATTGGG
CGTAAAGCGCGCGCAGGCGGTCTTTTAAGTCTGATGTGAAAGCCCACGGCTCAACCGTGGAGGGTCA
TTGGAAACTGGAAGACTTGAGTGCAGAAGAGGAGAGTGGAATTCCACGTGTAGCGGTGAAATGCGTA
GAGATGTGGAGGAACACCAGTGGCGAAGGCGACTCTCTGGTCTGTAACTGACGCTGAGGCGCGAAA
GCGTGGGGAGCAAACAGGATTAGATACCCTGGTAGTCCACGCCGTAAACGATGAGTGCTAAGTGTTAG
AGGGTTTCCGCCCTTTAGTGCTGCAGCTAACGCATTAAGCACTCCGCCTGGGGAGTACGACCGCAAGG
TTGAAACTCAAAGGAATTGACGGGGGCCCGCACAAGCGGTGGAGCATGTGGTTTAATTCGAAGCAAC
GCGAAGAACCTTACCAGGTCTTGACATCCTCTGACCTCCCTAGAGATAGGGCCTTCCCCTTTCGGGGG
ACAGAGTGACAGGTGGTGCATGGTGTCGTCAGCTCGTGTCGTGAGATGTTGGTAGTCCCGCACGAGC
GCACCCTTGACCTTAGTGCCAGCATTCAGTTGGGCACCTCCTAAGGTGACTGCCGGGT

Bacillus ginsengihumi strain Gsoil 114 16S ribosomal RNA gene, partial sequence	2010	2010	99%	0.0	98.68%	NR_041378.1
Bacillus shackletonii strain LMG 18435 16S ribosomal RNA gene, partial sequence	1840	1840	99%	0.0	95.97%	NR_025373.1
Bacillus acidicola strain 105-2 16S ribosomal RNA, partial sequence	1818	1818	99%	0.0	95.62%	NR_041942.1
Bacillus sporothermodurans strain M215 16S ribosomal RNA gene, partial sequence	1810	1810	99%	0.0	95.54%	NR_118832.1
Bacillus vini strain LAM0415 16S ribosomal RNA, partial sequence	1790	1790	99%	0.0	95.20%	NR_146819.1
Bacillus camelliae strain 7578-1 16S ribosomal RNA, partial sequence	1784	1784	99%	0.0	95.11%	NR_159341.1
Bacillus oleronius strain ATCC 700005 16S ribosomal RNA, partial sequence	1784	1784	99%	0.0	95.11%	NR_043325.1
Bacillus sporothermodurans strain M215 16S ribosomal RNA gene, partial sequence	1783	1783	99%	0.0	95.11%	NR_026010.1
Bacillus sporothermodurans strain M215 16S ribosomal RNA gene, partial sequence	1777	1777	99%	0.0	95.02%	NR_118833.1
Bacillus oleronius strain DSM 9356 16S ribosomal RNA, partial sequence	1773	1773	99%	0.0	94.94%	NR_119157.1

Bacillus ginsengihumi YMR7

CCATATCTGTCACCTTCGGCGGCTGGCTCCAAAAGGTTACCTCACCGACTTCGGGTGTTACAAACTCTC
GTGGTGTGACGGGCGGTGTGTACAAGGCCCGGGAACGTATTCACCGCGGCATGCTGATCCGCGATTAC
TAGCGATTCCAGCTTCATGCAGGCGAGTTGCAGCCTGCAATCCGAACTGAGAATGGTTTTATGGGATTG
GCTTAACCTCGCGGTCTCGCAGCCCTTTGTACCATCCATTGTAGCACGTGTGTAGCCCAGGTCATAAGG
GGCATGATGATTTGACGTCATCCCCACCTTCCTCCGGTTTGTCACCGGCAGTCACCTTAGAGTGCCCAA
CTGAATGCTGGCAACTAAGGTCAAGGGTTGCGCTCGTTGCGGGACTTAACCCAACATCTCACGACACG
AGCTGACGACAACCATGCACCACCTGTCACTCTGTCCCCCGAAGGGGAAGGCCCTATCTCTAGGGAG
GTCAGAGGATGTCAAGACCTGGTAAGGTTCTTCGCGTTGCTTCGAATTAAACCACATGCTCCACCGCT
TGTGCGGGCCCCCGTCAATTCCTTTGAGTTTCAACCTTGCGGTCGTACTCCCCAGGCGGAGTGCTTAAT
GCGTTAGCTGCAGCACTAAAGGGCGGAAACCCTCTAACACTTAGCACTCATCGTTTACGGCGTGGACT
ACCAGGGTATCTAATCCTGTTTGCTCCCCACGCTTTCGCGCCTCAGCGTCAGTTACAGACCAGAGAGT
CGCCTTCGCCACTGGTGTTCCTCCACATCTCTACGCATTTCACCGCTACACGTGGAATTCCACTCTCCT
CTTCTGCACTCAAGTCTTCCAGTTTCCAATGACCCTCCACGGTTGAGCCGTGGGCTTTCACATCAGACT
TAAAAGACCGCCTGCGCGCGCTTTACGCCCAATAATTCCGGACAACGCTTGCCACCTACGTATTACCGC
GGCTGCTGGCACGTAGTTAGCCGTGGCTTTCTGGTTAGGTACCGTCAAGGTACCGCCCTATTCGAACG
ATACTTGTTCTTCCCTAACACAGAGTTTTACGATCGAAGACTTCTTCACTCACGCGGCGTTGCTCGTCA
GACTTTCGTCATTGCGGAAGAATTCCTACTGC

Bacillus ginsengihumi strain Gsoil 114 16S ribosomal RNA, partial sequence	1979	1979	99%	0.0	99.20%	NR_041378.1
Bacillus shackletonii strain LMG 18435 16S ribosomal RNA, partial sequence	1922	1922	99%	0.0	98.05%	NR_025373.1
Bacillus acidicola strain 105-2 16S ribosomal RNA, partial sequence	1893	1893	99%	0.0	97.52%	NR_041942.1
Bacillus camelliae strain 7578-1 16S ribosomal RNA, partial sequence	1876	1876	99%	0.0	97.26%	NR_159341.1
Bacillus sporothermodurans strain M215 16S ribosomal RNA, partial sequence	1861	1861	99%	0.0	97.00%	NR_118833.1
Bacillus sporothermodurans strain M215 16S ribosomal RNA, partial sequence	1861	1861	99%	0.0	97.00%	NR_118832.1
Bacillus sporothermodurans strain M215 16S ribosomal RNA, partial sequence	1861	1861	99%	0.0	97.00%	NR_026010.1
Bacillus isabeliae strain CVS-8 16S ribosomal RNA, partial sequence	1855	1855	99%	0.0	96.91%	NR_042619.1
Bacillus dakarensis strain Marseille-P3515 16S ribosomal RNA, partial sequence	1847	1847	99%	0.0	96.65%	NR_147382.1
Bacillus gottheilii strain WCC 4585 16S ribosomal RNA, partial sequence	1847	1847	99%	0.0	96.56%	NR_108491.1
Bacillus vini strain LAM0415 16S ribosomal RNA, partial sequence	1841	1841	99%	0.0	96.89%	NR_146819.1
Bacillus oleronius strain ATCC 700005 16S ribosomal RNA, partial sequence	1838	1838	99%	0.0	96.65%	NR_043325.1

Pantoea dispersa GL1

GGGCTGGGCGGCGGCTACACATGCAGTCGAACGGCAGCACAGAAGAGCTTGCTCTTTGGGTGGCGAG
TGGCGGACGGGTGAGTAATGTCTGGGAAACTGCCCGATGGAGGGGGATAACTACTGGAAACGGTAGC
TAATACCGCATAACGTCGCAAGACCAAAGTGGGGGACCTTCGGGCCTCACACCATCGGATGTGCCCAG
ATGGGATTAGCTAGTAGGTGGGGTAATGGCTCACCTAGGCGACGATCCCTAGCTGGTCTGAGAGGATG
ACCAGCCACACTGGAACTGAGACACGGTCCAGACTCCTACGGGAGGCAGCAGTGGGGAATATTGCAC
AATGGGCGCAAGCCTGATGCAGCCATGCCGCGTGTATGAAGAAGGCCTTCGGGTTGTAAAGTACTTTC
AGCGGGGAGGAAGGCGGTGAGGTTAATAACCTCGCCGATTGACGTTACCCGCAGAAGAAGCACCGGC
TAACTCCGTGCCAGCAGCCGCGGTAATACGGAGGGTGCAAGCGTTAATCGGAATTACTGGGCGTAAAG
CGCACGCAGGCGGTCTGTTAAGTCAGATGTGAAATCCCCGGGCTTAACCTGGGAACTGCATTTGAAAC
TGGCAGGCTTGAGTCTCGTAGAGGGGGGTAGAATTCCAGGTGTAGCGGTGAAATGCGTAGAGATCTG

GAGGAATACCGGTGGCGAAGGCGGCCCCCTGGACGAAGACTGACGCTCAGGTGCGAAAGCGTGGGG
AGCAAACAGGATTAGATACCCTGGTAGTCCACGCCGTAAACGATGTCGACTTGGAGGTTGTGCCCTTG
AGGCGTGGCTTCCGGAGCTAACGCGTTAAGTCGACCGCCTGGGGAGTACGGCCGCAAGGTTAAAACT
CAAATGAATTGACGGGGGCCCGCACAAGCGGTGGAGCATGTGGTTTAATTCGATGCAACGCGAAGAA
CCTTACCTGGCCTTGACATCCAGAGAACTTAGCAGAGATGCTTTGTGCCTTCGGGAACTCTGAGACAG
GTGCTGCATCGTACTGTCGTCAGCTCGTGCTGTGAAATGGTGGGTAAGTTCCGCACGAGCGCACCTAA
TCGTTGTGCAGCGGCTCGGCGGGAACTCAAGGAGACTGCGGTGATTATTGAAGAAGGTGGGATGACG
TCAGTCATCATGGCTTACGCAAGGGCCTAACAGCACGT

Pantoea dispersa strain DSM 30073 16S ribosomal RNA gene, partial sequence	1857	1857	98%	0.0	94.68%	NR_116797.1
Pantoea dispersa strain LMG 2603 16S ribosomal RNA gene, partial sequence	1783	1783	92%	0.0	95.06%	NR_116755.1
Pantoea dispersa strain LMG 2603 16S ribosomal RNA gene, partial sequence	1779	1779	92%	0.0	94.97%	NR_043883.1
Kluyvera cryocrescens strain 12993 16S ribosomal RNA gene, partial sequence	1766	1766	97%	0.0	93.43%	NR_028803.1
Kluyvera cryocrescens strain NBRC 102467 16S ribosomal RNA gene, partial sequence	1764	1764	97%	0.0	93.34%	NR_114108.1
Kluyvera intermedia strain 256 16S ribosomal RNA gene, partial sequence	1753	1753	97%	0.0	93.26%	NR_028802.1
Pantoea stewartii subsp. indologenes strain CIP 104006 16S ribosomal RNA, partial sequence	1751	1751	98%	0.0	93.13%	NR_104928.1
Pantoea stewartii strain LMG 2715 16S ribosomal RNA gene, partial sequence	1749	1749	98%	0.0	93.04%	NR_119361.1
Kluyvera intermedia strain JCM1238 16S ribosomal RNA gene, partial sequence	1749	1749	97%	0.0	93.18%	NR_112007.1
Klebsiella aerogenes strain KCTC 2190 16S ribosomal RNA, complete sequence	1744	1744	97%	0.0	93.10%	NR_102493.2

Bacillus peanut GL1

TAGGCGGTGGGGGGGGTGCTATACATGCAAGTCGAGCGAACTGATTAGAAGCTTGCTTCTATGACGTT
AGCGGCGGACGGGTGAGTAACACGTGGGCAACCTGCCTGTAAGACTGGGATAACTTCGGGAAACCGA
AGCTAATACCGGATAGGATCTTCTCCTTCATGGGAGATGATTGAAAGATGGTTTCGGCTATCACTTACA
GATGGGCCCGCGGTGCATTAGCTAGTTGGTGAGGTAACGGCTCACCAAGGCAACGATGCATAGCCGAC
CTGAGAGGGTGATCGGCCACACTGGGACTGAGACACGGCCCAGACTCCTACGGGAGGCAGCAGTAG
GGAATCTTCCGCAATGGACGAAAGTCTGACGGAGCAACGCCGCGTGAGTGATGAAGGCTTTCGGGTC
GTAAAACTCTGTTGTTAGGGAAGAACAAGTACAAGAGTAACTGCTTGTACCTTGACGGTACCTAACCA
GAAAGCCACGGCTAACTACGTGCCAGCAGCCGCGGTAATACGTAGGTGGCAAGCGTTATCCGGAATTA
TTGGGCGTAAAGCGCGCGCAGGCGGTTTCTTAAGTCTGATGTGAAAGCCCACGGCTCAACCGTGGAG
GGTCATTGGAAACTGGGGAACTTGAGTGCAGAAGAGAAAAGCGGAATTCCACGTGTAGCGGTGAAAT
GCGTAGAGATGTGGAGGAACACCAGTGGCGAAGGCGGCTTTTTGGTCTGTAACTGACGCTGAGGCGC
GAAAGCGTGGGGAGCAAACAGGATTAGATACCCTGGTAGTCCACGCCGTAAACGATGAGTGCTAAGT
GTTAGAGGGTTTCCGCCCTTTAGTGCTGCAGCTAACGCATTAAGCACTCCGCCTGGGGAGTACGGTCG
CAAGACTGAAACTCAAAGGAATTGACGGGGGCCCGCACAAGCGGTGGAGCATGTGTTTTAATTCGAA
GCAACGCGAAGAACCTTACCAGGTCTTGACATCCTCTGAACAACTCTAGAGATAGAGCGTTCCCCTTT
CGGGGGACAGAGTGACAGGTGGGTGCATGGTTGTCGTCAGCTCGTGTCGTGAGATGTTGGGTTAGTC
CCGCACGAGCGCACCTTGATCTAGTTGCCAGCATTAGTGGCACCTCTAGGTGACTGCCGGTGACCAAT
CGAGATGGTGGGGATGACGTTCATCATCATGCCCCGTTATGACCTGGCTATACACCACCGCGTTGTCCT
CATCAAA

Bacillus megaterium strain ATCC 14581 16S ribosomal RNA, partial sequence	1987	1987	96%	0.0	97.82%	NR_116873.1
Bacillus megaterium strain ATCC 14581 16S ribosomal RNA, partial sequence	1987	1987	96%	0.0	97.82%	NR_117473.1
Bacillus megaterium NBRC 15308 = ATCC 14581 16S ribosomal RNA, partial sequence	1987	1987	96%	0.0	97.82%	NR_112636.1
Bacillus megaterium strain IAM 13418 16S ribosomal RNA, partial sequence	1978	1978	96%	0.0	97.66%	NR_043401.1
Bacillus aryabhattai B8W22 16S ribosomal RNA, partial sequence	1978	1978	96%	0.0	97.66%	NR_115953.1
Bacillus flexus strain NBRC 15715 16S ribosomal RNA, partial sequence	1938	1938	96%	0.0	96.90%	NR_113800.1
Bacillus flexus strain IFO15715 16S ribosomal RNA, partial sequence	1938	1938	96%	0.0	96.90%	NR_024691.1
Bacillus flexus strain SBMP3 16S ribosomal RNA, partial sequence	1929	1929	96%	0.0	96.91%	NR_118382.1
Bacillus simplex NBRC 15720 = DSM 1321 16S ribosomal RNA, partial sequence	1914	1914	96%	0.0	96.57%	NR_115603.1
Bacillus qingshengii strain G19 16S ribosomal RNA, partial sequence	1900	1900	96%	0.0	96.08%	NR_133978.1
Bacillus aryabhattai B8W22 16S ribosomal RNA, partial sequence	1861	1861	91%	0.0	97.52%	NR_118442.1
Bacillus megaterium strain DSM 32 16S ribosomal RNA, partial sequence	1805	1805	96%	0.0	93.81%	NR_118962.1
Bacillus iocasae strain S36 16S ribosomal RNA, partial sequence	1787	1787	96%	0.0	94.32%	NR_158045.1
Bacillus paraflexus strain RC2 16S ribosomal RNA, partial sequence	1777	1777	95%	0.0	95.03%	NR_135732.1
Bacillus abyssalis strain SCSIO 15042 16S ribosomal RNA, partial sequence	1774	1774	95%	0.0	94.27%	NR_109671.1

Bacillus peanut GL2

TAGGCCATTGGCGGGCGTGCTTATACATGCAAGTCGAGCGAACTGATTAGAAGCTTGCTTCTATGACGT
TAGCGGCGGACGGGTGAGTAACACGTGGGCAACCTGCCTGTAAGACTGGGATAACTTCGGGAAACCG
AAGCTAATACCGGATAGGATCTTCTCCTTCATGGGAGATGATTGAAAGATGGTTTCGGCTATCACTTAC
AGATGGGCCCGCGGTGCATTAGCTAGTTGGTGAGGTAACGGCTCACCAAGGCAACGATGCATAGCCGA
CCTGAGAGGGTGATCGGCCACACTGGGACTGAGACACGGCCCAGACTCCTACGGGAGGCAGCAGTA
GGGAATCTTCCGCAATGGACGAAAGTCTGACGGAGCAACGCCGCGTGAGTGATGAAGGCTTTCGGGT
CGTAAAACTCTGTTGTTAGGGAAGAACAAGTACGAGAGTAACTGCTCGTACCTTGACGGTACCTAACC
AGAAAGCCACGGCTAACTACGTGCCAGCAGCCGCGGTAATACGTAGGTGGCAAGCGTTATCCGGAATT
ATTGGGCGTAAAGCGCGCGCAGGCGGTTTCTTAAGTCTGATGTGAAAGCCCACGGCTCAACCGTGGA
GGGTCATTGGAAACTGGGGAACTTGAGTGCAGAAGAGAAAAGCGGAATTCCACGTGTAGCGGTGAA
ATGCGTAGAGATGTGGAGGAACACCAGTGGCGAAGGCGGCTTTTTGGTCTGTAACTGACGCTGAGGC
GCGAAAGCGTGGGGAGCAAACAGGATTAGATACCCTGGTAGTCCACGCCGTAAACGATGAGTGCTAA
GTGTTAGAGGGTTTCCGCCCTTTAGTGCTGCAGCTAACGCATTAAGCACTCCGCCTGGGGAGTACGGT
CGCAAGACTGAAACTCAAAGGAATTGACGGGGGCCCGCACAAGCGGTGGAGCATGTGGTTTAATTCG
AAGCAACGCGAAGAACCTTACCAGGTCTTGACATCCTCTGACAACTCTAGAGATAGAGCGTTTCCCCT
TCGGGGGAAAAGAGTGACAGGTGGTGCATGGATTGTCGTCAGCTCGTGTCGTGAGATGTTGGGTTA
AGTTCCCGCAACGGAGCGCATCCTTGATCTTAGTTGCCAGCATTTAGTGGCACTCTTAGTGACTGCCGG
TGACATCGGAGAGGGTGGGATGACGTCAATTCATCATGCCCTTATGACTGGCATACACACACCGGTGT
CTCTACTACAAAATG

Bacillus aryabhattai strain B8W22 16S ribosomal RNA gene, partial sequence	2007	2007	97%	0.0	97.69%	NR_115953.1
Bacillus megaterium strain ATCC 14581 16S ribosomal RNA gene, partial sequence	1998	1998	97%	0.0	97.52%	NR_116873.1
Bacillus megaterium strain NBRC 15308 16S ribosomal RNA gene, partial sequence	1998	1998	97%	0.0	97.52%	NR_112636.1
Bacillus megaterium strain ATCC 14581 16S ribosomal RNA gene, partial sequence	1995	1995	97%	0.0	97.44%	NR_117473.1
Bacillus megaterium strain IAM 13418 16S ribosomal RNA gene, partial sequence	1989	1989	97%	0.0	97.36%	NR_043401.1
Bacillus flexus strain NBRC 15715 16S ribosomal RNA gene, partial sequence	1948	1948	97%	0.0	96.61%	NR_113800.1
Bacillus flexus strain IFO15715 16S ribosomal RNA gene, partial sequence	1948	1948	97%	0.0	96.61%	NR_024691.1
Bacillus flexus strain SBMP3 16S ribosomal RNA gene, partial sequence	1943	1943	96%	0.0	96.92%	NR_118382.1
Bacillus simplex strain DSM 1321 16S ribosomal RNA gene, partial sequence	1925	1925	97%	0.0	96.28%	NR_115603.1
Bacillus qingshengii strain G19 16S ribosomal RNA, partial sequence	1922	1922	95%	0.0	96.64%	NR_133978.1
Bacillus aryabhattai strain B8W22 16S ribosomal RNA gene, partial sequence	1884	1884	91%	0.0	97.79%	NR_118442.1
Bacillus megaterium strain DSM 32 16S ribosomal RNA gene, partial sequence	1817	1817	97%	0.0	93.56%	NR_118962.1
Bacillus iocasae strain S36 16S ribosomal RNA, partial sequence	1811	1811	97%	0.0	94.31%	NR_158045.1
Bacillus cohnii strain NBRC 15565 16S ribosomal RNA gene, partial sequence	1793	1793	97%	0.0	93.72%	NR_113776.1
Bacillus cohnii strain DSM 6307 16S ribosomal RNA gene, partial sequence	1787	1787	97%	0.0	93.64%	NR_026138.1

Bacillus peanut GL3

TAGGGAAGTTGGCGGGCGTGCTATACATGCAGTCGAGCGAACTGATTAGAAGCTTGCTTCTATGACGT
TAGCGGCGGACGGGTGAGTAACACGTGGGCAACCTGCCTGTAAGACTGGGATAACTTCGGGAAACCG
AAGCTAATACCGGATAGGATCTTCTCCTTCATGGGAGATGATTGAAAGATGGTTTCGGCTATCACTTAC
AGATGGGCCCGCGGTGCATTAGCTAGTTGGTGAGGTAACGGCTCACCAAGGCAACGATGCATAGCCGA
CCTGAGAGGGTGATCGGCCACACTGGGACTGAGACACGGCCCAGACTCCTACGGGAGGCAGCAGTA
GGGAATCTTCCGCAATGGACGAAAGTCTGACGGAGCAACGCCGCGTGAGTGATGAAGGCTTTCGGGT
CGTAAAACTCTGTTGTTAGGGAAGAACAAGTACGAGAGTAACTGCTCGTACCTTGACGGTACCTAACC
AGAAAGCCACGGCTAACTACGTGCCAGCAGCCGCGGTAATACGTAGGTGGCAAGCGTTATCCGGAATT
ATTGGGCGTAAAGCGCGCGCAGGCGGTTTCTTAAGTCTGATGTGAAAGCCCACGGCTCAACCGTGGA
GGGTCATTGGAAACTGGGGAACTTGAGTGCAGAAGAGAAAAGCGGAATTCCACGTGTAGCGGTGAA
ATGCGTAGAGATGTGGAGGAACACCAGTGGCGAAGGCGGCTTTTTGGTCTGTAACTGACGCTGAGGC
GCGAAAGCGTGGGGAGCAAACAGGATTAGATACCCTGGTAGTCCACGCCGTAAACGATGAGTGCTAA

GTGTTAGAGGGTTTCCGCCCTTTAGTGCTGCAGCTAACGCATTAAGCACTCCGCCTGGGGAGTACGGT
CGCAAGACTGAAACTCAAAGGAATTGACGGGGGCCCGCACAAGCGGTGGAGCATGTGGTTTAATTTC
GAAGCAACGCGAAGAACCTTACCAGGTCTTGACATCCTCTGACACTCTAGAGATAGAGCGTTCCCCTT
CGGGGACAGAGTGACAGGTGTGCATGGTTGTCGTCAGCTCGTGTCGTGAGAATGTTGGCTAAGGTCC
CGGCACCGAGCGCACCATGATCTAGCTGCAGCAATTTAGTGGCACTCTAGTGACTGCCGTGACCGTCG
AAGAGGTGGATGACGTCACTCATCATGGCCGTATTGACGTGGCCTATAACAACA
GCGGTGC

	score	score	cover	value		
Bacillus aryabhattai strain B8W22 16S ribosomal RNA gene, partial sequence	1907	1907	98%	0.0	96.18%	NR_115953.1
Bacillus megaterium strain ATCC 14581 16S ribosomal RNA gene, partial sequence	1898	1898	98%	0.0	96.01%	NR_116873.1
Bacillus megaterium strain NBRC 15308 16S ribosomal RNA gene, partial sequence	1898	1898	98%	0.0	96.01%	NR_112636.1
Bacillus megaterium strain ATCC 14581 16S ribosomal RNA gene, partial sequence	1895	1895	98%	0.0	95.93%	NR_117473.1
Bacillus megaterium strain IAM 13418 16S ribosomal RNA gene, partial sequence	1889	1889	98%	0.0	95.85%	NR_043401.1
Bacillus flexus strain SBMP3 16S ribosomal RNA gene, partial sequence	1850	1850	96%	0.0	95.55%	NR_118382.1
Bacillus flexus strain NBRC 15715 16S ribosomal RNA gene, partial sequence	1848	1848	98%	0.0	95.10%	NR_113800.1
Bacillus flexus strain IFO15715 16S ribosomal RNA gene, partial sequence	1848	1848	98%	0.0	95.10%	NR_024691.1
Bacillus qingshengii strain G19 16S ribosomal RNA, partial sequence	1838	1838	95%	0.0	95.57%	NR_133978.1
Bacillus simplex strain DSM 1321 16S ribosomal RNA gene, partial sequence	1825	1825	98%	0.0	94.77%	NR_115603.1
Bacillus aryabhattai strain B8W22 16S ribosomal RNA gene, partial sequence	1795	1795	91%	0.0	96.35%	NR_118442.1
Bacillus megaterium strain DSM 32 16S ribosomal RNA gene, partial sequence	1720	1720	98%	0.0	92.19%	NR_118962.1
Bacillus iocasae strain S36 16S ribosomal RNA, partial sequence	1711	1711	98%	0.0	92.79%	NR_158045.1
Bacillus abyssalis strain SCSIO 15042 16S ribosomal RNA gene, partial sequence	1700	1700	96%	0.0	92.90%	NR_109671.1
Bacillus cohnii strain NBRC 15565 16S ribosomal RNA gene, partial sequence	1698	1698	98%	0.0	92.28%	NR_113776.1

Bacillus peanut GL4

AGCGTGCGGGGAGCTATACATGCAGTCGAGCGGACAGATGGGAGCTTGCTCCCTGATGTTAGCGGCG
GACGGGTGAGTAACACGTGGGTAACCTGCCTGTAAGACTGGGATAACTCCGGGAAACCGGGGCTAAT
ACCGGATGGTTGTCTGAACCGCATGGTTCAGACATAAAAGGTGGCTTCGGCTACCACTTACAGATGGA
CCCGCGGCGCATTAGCTAGTTGGTGAGGTAACGGCTCACCAAGGCGACGATGCGTAGCCGACCTGAG
AGGGTGATCGGCCACACTGGGACTGAGACACGGCCCAGACTCCTACGGGAGGCAGCAGTAGGGAATC
TTCCGCAATGGACGAAAGTCTGACGGAGCAACGCCGCGTGAGTGATGAAGGTTTTCGGATCGTAAAG
CTCTGTTGTTAGGGAAGAACAAGTGCCGTTCAAATAGGGCGGCACCTTGACGGTACCTAACCAGAAA
GCCACGGCTAACTACGTGCCAGCAGCCGCGGTAATACGTAGGTGGCAAGCGTTGTCCGGAATTATTGG
GCGTAAAGGGCTCGCAGGCGGTTTCTTAAGTCTGATGTGAAAGCCCCCGGCTCAACCGGGGAGGGTC
ATTGGAAACTGGGGAACTTGAGTGCAGAAGAGGAGAGTGGAATTCCACGTGTAGCGGTGAAATGCGT
AGAGATGTGGAGGAACACCAGTGGCGAAAGGCGACTCTCTGGTCTGTAACTGACGCTGAGGAGCGA
AAGCGTGGGGAGCGAACAGGATTAGATACCCTGGTAGTCCACGCCGTAAACGATGAGTGCTAAGTGTT
AGGGGGTTTCCGCCCCTTAGTGCTGCAGCTAACGCATTAAGCACTCCGCCTGGGGGAGTACGGTCGCA
GACTGAAACTCAAAGGAATTGACGGGGGCCCGCACAGCGTGAGCATGTGTTTAATTCGAGCACGCGA
GACTTACAGTCTGACATCTCTGACATCTAGAGATAGACGTCCCTCCGGGGCAGAGTGACAGTGTGCAT
GCTGTCGTCAGCTCAGTCCTGAATGGTGGGTAGGTCGCACGAGCCAATCATTGATCTAGTGCAGCATC
AGTCGCACTTAGTACTGACGGACATGAGAGTGGCATGACGTCAAAACTCATGGCCGCTAG

Bacillus velezensis strain FZB42 16S ribosomal RNA, complete sequence	1663	1663	92%	0.0	95.74%	NR_075005.2
Bacillus amyloliquefaciens strain MPA 1034 16S ribosomal RNA gene, partial sequence	1659	1659	92%	0.0	95.65%	NR_117946.1
Bacillus amyloliquefaciens strain NBRC 15535 16S ribosomal RNA gene, partial sequence	1659	1659	92%	0.0	95.65%	NR_041455.1
Bacillus amyloliquefaciens strain NBRC 15535 16S ribosomal RNA gene, partial sequence	1655	1655	92%	0.0	95.56%	NR_112685.1
Bacillus methylotrophicus strain CBMB205 16S ribosomal RNA gene, partial sequence	1654	1654	92%	0.0	95.58%	NR_116240.1
Bacillus amyloliquefaciens strain BCRC 11601 16S ribosomal RNA gene, partial sequence	1654	1654	92%	0.0	95.56%	NR_116022.1
Bacillus subtilis subsp. subtilis strain 168 16S ribosomal RNA, complete sequence	1650	1650	92%	0.0	95.47%	NR_102783.2
Bacillus siamensis strain PD-A10 16S ribosomal RNA gene, partial sequence	1650	1650	92%	0.0	95.47%	NR_117274.1
Bacillus vallismortis strain DSM 11031 16S ribosomal RNA gene, partial sequence	1645	1645	92%	0.0	95.37%	NR_024696.1
Bacillus tequilensis strain 10b 16S ribosomal RNA gene, partial sequence	1643	1643	92%	0.0	95.28%	NR_104919.1
Bacillus vallismortis strain NBRC 101236 16S ribosomal RNA gene, partial sequence	1642	1642	92%	0.0	95.28%	NR_113994.1
Bacillus atrophaeus strain NBRC 15539 16S ribosomal RNA gene, partial sequence	1642	1642	92%	0.0	95.28%	NR_112723.1
Bacillus nakamurai strain NRRL B-41091 16S ribosomal RNA, partial sequence	1641	1641	92%	0.0	95.28%	NR_151897.1
Bacillus subtilis strain JCM 1465 16S ribosomal RNA gene, partial sequence	1641	1641	92%	0.0	95.28%	NR_113265.1
Bacillus subtilis strain NBRC 13719 16S ribosomal RNA gene, partial sequence	1641	1641	92%	0.0	95.28%	NR_112629.1
Bacillus subtilis strain BCRC 10255 16S ribosomal RNA gene, partial sequence	1641	1641	92%	0.0	95.28%	NR_116017.1

Bacillus peanut GL5

AACATTGCGGCGTGCTATACATGCAAGTCGAGCGAATGGATTAAGAGCTTGCTCTTATGAAGTTAGCGG

CGGACGGGTGAGTAACACGTGGGTAACCTGCCCATAAGACTGGGATAACTCCGGGAAACCGGGGCTA
ATACCGGATAACATTTTGCACCGCATGGTGCGAAATTGAAAGGCGGCTTCGGCTGTCACTTATGGATGG
ACCCGCGTCGCATTAGCTAGTTGGTGAGGTAACGGCTCACCAAGGCAACGATGCGTAGCCGACCTGA
GAGGGTGATCGGCCACACTGGGACTGAGACACGGCCCAGACTCCTACGGGAGGCAGCAGTAGGGAA
TCTTCCGCAATGGACGAAAGTCTGACGGAGCAACGCCGCGTGAGTGATGAAGGCTTTCGGGTCGTAA
AACTCTGTTGTTAGGGAAGAACAAGTGCTAGTTGAATAAGCTGGCACCTTGACGGTACCTAACCAGAA
AGCCACGGCTAACTACGTGCCAGCAGCCGCGGTAATACGTAGGTGGCAAGCGTTATCCGGAATTATTG
GGCGTAAAGCGCGCGCAGGTGGTTTCTTAAGTCTGATGTGAAAGCCCACGGCTCAACCGTGGAGGGT
CATTGGAAACTGGGAGACTTGAGTGCAGAAGAGGAAAGTGGAATTCCATGTGTAGCGGTGAAATGCG
TAGAGATATGGAGGAACACCAGTGGCGAAGGCGACTTTCTGGTCTGTAACTGACACTGAGGCGCGAA
AGCGTGGGGAGCAAACAGGATTAGATACCCTGGTAGTCCACGCCGTAAACGATGAGTGCTAAGTGTTA
GAGGGTTTCCGCCCTTTAGTGCTGAAGTTAACGCATTAAGCACTCCGCCTGGGGAGTACGGCCGCAA
GGCTGAAACTCAAAGGAATTGACGGGGGCCCGCACAAGCGGTGGAGCATGTGGTTTAATTCGAAGCA
ACGCGAAGAACCTTACCAGTCTTGACATCCTCTGAAAACCCTAGAGATAGGCTTCTCCTTCGGGAGCA
GAGTGACAGGTGGTGCATGGTGTCGTCAGCTCGTGTCGTGAGATGTTGGGTTAAGTCCCGCAACGA
GCGCAACCCTTGATCTTAGTTGCCATCATTAAGTGGCACTCTAAGGGTGACTGCCGGTGACGAGCGAG
GACGTGGGATGACGTTCAATCATCATGCCCTTATGGACTGGCTTACACACGTGCTACATGGACGTACAA
GCTGCAGACGCAGTGAGCTATCCTCATAAACGCGATCTCA

Bacillus cereus ATCC 14579 16S ribosomal RNA (rrnA), partial sequence	2039	2039	99%	0.0	96.54%	NR_074540.1
Bacillus cereus strain JCM 2152 16S ribosomal RNA, partial sequence	2039	2039	99%	0.0	96.54%	NR_113266.1
Bacillus cereus strain CCM 2010 16S ribosomal RNA, partial sequence	2039	2039	99%	0.0	96.54%	NR_115714.1
Bacillus cereus strain NBRC 15305 16S ribosomal RNA, partial sequence	2039	2039	99%	0.0	96.54%	NR_112630.1
Bacillus cereus ATCC 14579 16S ribosomal RNA, partial sequence	2039	2039	99%	0.0	96.54%	NR_114582.1
Bacillus cereus strain IAM 12605 16S ribosomal RNA, partial sequence	2039	2039	99%	0.0	96.54%	NR_115526.1
Bacillus wiedmannii strain FSL W8-0169 16S ribosomal RNA, partial sequence	2035	2035	99%	0.0	96.47%	NR_152692.1
Bacillus proteolyticus strain MCCC 1A00365 16S ribosomal RNA, partial sequence	2035	2035	99%	0.0	96.47%	NR_157735.1
Bacillus albus strain MCCC 1A02146 16S ribosomal RNA, partial sequence	2035	2035	99%	0.0	96.47%	NR_157729.1
Bacillus paramycoides strain MCCC 1A04098 16S ribosomal RNA, partial sequence	2030	2030	99%	0.0	96.39%	NR_157734.1
Bacillus pseudomycoides strain NBRC 101232 16S ribosomal RNA, partial sequence	2030	2030	99%	0.0	96.39%	NR_113991.1
Bacillus pacificus strain MCCC 1A06182 16S ribosomal RNA, partial sequence	2026	2026	99%	0.0	96.31%	NR_157733.1
Bacillus paranthracis strain MCCC 1A00395 16S ribosomal RNA, partial sequence	2026	2026	99%	0.0	96.31%	NR_157728.1
Bacillus toyonensis strain BCT-7112 16S ribosomal RNA, partial sequence	2021	2021	99%	0.0	96.23%	NR_121761.1
Bacillus thuringiensis strain ATCC 10792 16S ribosomal RNA, partial sequence	2021	2021	99%	0.0	96.23%	NR_114581.1
Bacillus thuringiensis strain IAM 12077 16S ribosomal RNA, partial sequence	2021	2021	99%	0.0	96.23%	NR_043403.1
Bacillus thuringiensis strain NBRC 101235 16S ribosomal RNA, partial sequence	2018	2018	99%	0.0	96.15%	NR_112780.1
Bacillus mobilis strain MCCC 1A05942 16S ribosomal RNA, partial sequence	2017	2017	99%	0.0	96.15%	NR_157731.1

Bacillus peanut GL6

CCATTCTGTCACCTTCGGCGGCTGGCTCCAAAAGGTTACCTCACCGACTTCCGGTGTTACAAACTCTC
GTGGTGTGACGGGCGGTGTGTACAAGGGCCGGGAACGTATTCACCGCGGCATGCTGATCCCCGATTAC
TAGCGATTCCAGCTTCATGGAGGCGAGTTGCAGCCTGCAATCCGAACTGAGAATGGTTTTATGGGATT
GGCTTAACCTCGCGGTCTCGCAGCCCTTTGTACCATCCATTGGAGCACGTGTGTAGCCCAGGGCATAA
GGGGCATGATGATTTGACGTCATCCCCACCTTCCTCCGGTTTGGCACCGGCAGTCACCTTAAAGTGGC
CAACTGAATGGTGGCAACTAAGGTCAAGGGTTGCGCTCGTTGCGGGACTTAACCCAACATCTCACGA
CACGAGCTGACGACCACCATGCACCACCTGTCACTCTGGCCCCCGAAGGGGAAGGCCCTATCTCTAG
GGAGGTCAAAAGATGTCAAGACCTGGAAAGGTTCTTCGCGTTGCTTCAAATTAAACCACATGCTCCAC
CGCTTGGGGGGGCCCCCGTCAATTCCTTTGAGTTTCAACCTTGGGGTCGTACTCCCCAGGGGGAGTGT
TTAATGCGTTAGCTGCAGCACTAAAGGGGGGAAACCCTCTAACACTTAGCACTCATCGTTTACGGCGG
GGACTACCAGGGTATCTAATCCTGTTTGCTCCCCACGCTTTCGCGCCTCAGCGTCAGTTACAGACCAGA
GAGTCCCCTTCCCCACTGGGTGTTTCCTCCACATCTCTACGCATTTCACCGCTACACGTGGAATTCCAC
TTCTCCTCTTCTGCACTCAAGTCCTCCCAGTTCCAATAGACCCTTCCACGGGGTGAACCCGGGGGGTC
TTTCAACTACAGAACTTAAAAAGACCCGCCTGGCGCGCGCTTTACGCCAAATAATTCCGGGACAAGCG
CTTGGCCCACCCTACGATATACCGCGGCTGTCTGGCACGTAAGTTAGCCGGTGGCTCTTCGGGATAGGT

ACCCGTCAAGGTACACGCCTAATCGATACTGATACTTGGTCTTCCCTAACCAACAGAGTTTACGATCCG
AAGACCCTTCTTCACTCACGCGGGGTTGCTCGTCGAACTTCGCTCATTGCGAGAGGATTCCTACGTGC
TGCCTCCGTAAGGACTAGGCCGGGTCCTAAGTCCCAATGTGGCGACATCACT

Bacillus ginsengihumi strain Gsoil 114 16S ribosomal RNA, partial sequence	1614	1614	98%	0.0	91.95%	NR_041378.1
Bacillus shackletonii strain LMG 18435 16S ribosomal RNA, partial sequence	1570	1570	98%	0.0	91.13%	NR_025373.1
Bacillus acidicola strain 105-2 16S ribosomal RNA, partial sequence	1545	1545	98%	0.0	90.71%	NR_041942.1
Bacillus sporothermodurans strain M215 16S ribosomal RNA, partial sequence	1526	1526	98%	0.0	90.47%	NR_118833.1
Bacillus sporothermodurans strain M215 16S ribosomal RNA, partial sequence	1526	1526	98%	0.0	90.47%	NR_118832.1
Bacillus sporothermodurans strain M215 16S ribosomal RNA, partial sequence	1526	1526	98%	0.0	90.47%	NR_026010.1
Bacillus camelliae strain 7578-1 16S ribosomal RNA, partial sequence	1524	1524	98%	0.0	90.40%	NR_159341.1
Bacillus dakarensis strain Marseille-P3515 16S ribosomal RNA, partial sequence	1522	1522	98%	0.0	90.15%	NR_147382.1
Bacillus oleronius strain ATCC 700005 16S ribosomal RNA, partial sequence	1522	1522	98%	0.0	90.22%	NR_043325.1
Bacillus gottheilii strain WCC 4585 16S ribosomal RNA, partial sequence	1517	1517	98%	0.0	89.98%	NR_108491.1
Bacillus acidiproducens strain SL213 16S ribosomal RNA, partial sequence	1510	1510	97%	0.0	90.43%	NR_116000.1
Bacillus vini strain LAM0415 16S ribosomal RNA, partial sequence	1508	1508	98%	0.0	90.33%	NR_146819.1

Bacillus peanut GL7

GCCATGGCGGGTGCTATACATGCAGTCGAGCGAACTGGATGAAGAGCTTGCTTTTAGTATCAGTTAGC
GGCGGACGGGTGAGTAACACGTGGGTAACCTGCCCAGTAAGACTAGGATAACTCCGGGAAACCGGGG
CTAATACTGGATAACTTTTCTCTCCGCATGGAGGGAGATTGAAAGATGGCTTCGGCTGTCACTTACGGA
TGGACCCGCGGCGCATTAGCTAGTTGGTGAGGTAACGGCTCACCAAGGCAACGATGCGTAGCCGACC
TGAGAGGGTGATCGGCCACATTGGGACTGAGACACGGCCCAAACTCCTACGGGAGGCAGCAGTAGGG
AATCTTCCGCAATGGACGAAAGTCTGACGGAGCAACGCCGCGTGAGTGAAGAAGGTCTTCGGATCGT
AAAACTCTGTTGTTAGGGAAGAACAAGTATCGTTCGAATAGGGCGGTACCTTGACGGTACCTAACCAG
AAAGCCACGGCTAACTACGTGCCAGCAGCCGCGGTAATACGTAGGTGGCAAGCGTTGTCCGGAATTAT
TGGGCGTAAAGCGCGCGCAGGCGGTCTTTTAAGTCTGATGTGAAAGCCCACGGCTCAACCGTGGAGG
GTCATTGGAAACTGGAAGACTTGAGTGCAGAAGAGGAGAGTGGAATTCCACGTGTAGCGGTGAAATG
CGTAGAGATGTGGAGGAACACCAGTGGCGAAGGCGACTCTCTGGTCTGTAACTGACGCTGAGGCGCG
AAAGCGTGGGGAGCAAACAGGATTAGATACCCTGGTAGTCCACGCCGTAAACGATGAGTGCTAAGTG
TTAGAGGGTTTCCGCCCTTTAGTGCTGCAGCTAACGCATTAAGCACTCCGCCTGGGGAGTACGACCGC
AAGGTTGAAACTCAAGGAATTGACGGGGGCCCGCACAAGCGGTGGAGCATGTGGTTTAATTCGAAGC
AACGCGAGAACCTTACCAGTCTTGACATCCTCTGACCTCCCTAGAGATAGGGCTTCCCCTTCGGAGGA
CAGAGTGACAGGGTGGTGCATGGATGTCGTCAGTCTCGTGTCGTGAAGATGTGGTTAGTCCCGAACGA
GCGCCACCCTTTGAACTTTAGTGCCCAGCATTCAGTTGGGCACTTCTAAGTAACTGCGTGACAATCGA
GAAGGGTGGATGACCTCAATCATCATGCCTA

Bacillus ginsengihumi strain Gsoil 114 16S ribosomal RNA gene, partial sequence	1932	1932	99%	0.0	96.31%	NR_041378.1
Bacillus shackletonii strain LMG 18435 16S ribosomal RNA gene, partial sequence	1773	1773	99%	0.0	93.89%	NR_025373.1
Bacillus acidicola strain 105-2 16S ribosomal RNA, partial sequence	1757	1757	99%	0.0	93.63%	NR_041942.1
Bacillus sporothermodurans strain M215 16S ribosomal RNA gene, partial sequence	1755	1755	99%	0.0	93.63%	NR_118832.1
Bacillus camelliae strain 7578-1 16S ribosomal RNA, partial sequence	1735	1735	99%	0.0	93.32%	NR_159341.1
Bacillus vini strain LAM0415 16S ribosomal RNA, partial sequence	1733	1733	99%	0.0	93.36%	NR_146819.1
Bacillus sporothermodurans strain M215 16S ribosomal RNA gene, partial sequence	1727	1727	99%	0.0	93.23%	NR_026010.1
Bacillus oleronius strain ATCC 700005 16S ribosomal RNA, partial sequence	1724	1724	99%	0.0	93.15%	NR_043325.1
Bacillus sporothermodurans strain M215 16S ribosomal RNA gene, partial sequence	1722	1722	99%	0.0	93.15%	NR_118833.1
Bacillus oleronius strain DSM 9356 16S ribosomal RNA, partial sequence	1714	1714	99%	0.0	93.06%	NR_119157.1

Bacillus peanut GL8

GAGCCTGCGGCGTGCTATACATGCAAGTCGAGCGAACTGATGAAGAGCTTGCTTTTGATCAGTTAGCG
GCGGACGGGTGAGTAACACGTGGGTAACCTGCCTGTAAGACTAGGATAACTCCGGGAAACCGGGGCT
AATACTGGATAACTTTTCTCTCCGCATGGAGAGAGATTGAAAGATGGCTTCGGCTATCACTTACAGATG
GACCCGCGGCGCATTAGCTAGTTGGTGAGGTAACGGCTCACCAAGGCAACGATGCGTAGCCGACCTG
AGAGGGTGATCGGCCACATTGGGACTGAGACACGGCCCAAACTCCTACGGGAGGCAGCAGTAGGGA
ATCTTCCGCAATGGACGAAAGTCTGACGGAGCAACGCCGCGTGAGTGAAGAAGGTCTTCGGATCGTA
AAACTCTGTTGTTAGGGAAGAACAAGTATCGTTCGAATAGGGCGGTACCTTGACGGTACCTAACCAGA
AAGCCACGGCTAACTACGTGCCAGCAGCCGCGGTAATACGTAGGTGGCAAGCGTTGTCCGGAATTATT

GGGCGTAAAGCGCGCGCAGGCGGTCTTTTAAGTCTGATGTGAAAGCCCACGGCTCAACCGTGGAGGG
TCATTGGAAACTGGAAGACTTGAGTGCAGAAGAGGAGAGTGGAATTCCACGTGTAGCGGTGAAATGC
GTAGAGATGTGGAGGAACACCAGTGGCGAAGGCGACTCTCTGGTCTGTAACTGACGCTGAGGCGCGA
AAGCGTGGGGAGCAAACAGGATTAGATACCCTGGTAGTCCACGCCGTAAACGATGAGTGCTAAGTGTT
AGAGGGTTTCCGCCCTTTAGTGCTGCAGCTAACGCATTAAGCACTCCGCCTGGGGAGTACGACCGCAA
GGTTGAAACTCAAAGGAATTGACGGGGGCCCGCACAAGCGGTGGAGCATGTGGTTTAATTCGAAGCA
ACGCGAAGAACCTTACCAGGTCTTGACATCCTCTGACCTCCCTAGAGATAGGCCCTTCCCCTTCGGGG
GACAGAGTGACAGTGGTGCATGGTGTCGTCAGCTCGTGTCGTGAGATGTGGGTTAGTCCCGCAACGA
GCGCAACCCTTGACCTAGTTGCAGGCATCAGTTGGGCACTCTAAGGGTGAACT

Bacillus ginsengihumi strain Gsoil 114 16S ribosomal RNA gene, partial sequence	2013	2013	99%	0.0	98.94%	NR_041378.1
Bacillus shackletonii strain LMG 18435 16S ribosomal RNA gene, partial sequence	1838	1838	99%	0.0	96.11%	NR_025373.1
Bacillus acidicola strain 105-2 16S ribosomal RNA, partial sequence	1816	1816	99%	0.0	95.76%	NR_041942.1
Bacillus sporothermodurans strain M215 16S ribosomal RNA gene, partial sequence	1803	1803	99%	0.0	95.59%	NR_118832.1
Bacillus camelliae strain 7578-1 16S ribosomal RNA, partial sequence	1783	1783	99%	0.0	95.25%	NR_159341.1
Bacillus sporothermodurans strain M215 16S ribosomal RNA gene, partial sequence	1783	1783	99%	0.0	95.25%	NR_026010.1
Bacillus oleronius strain ATCC 700005 16S ribosomal RNA, partial sequence	1777	1777	99%	0.0	95.16%	NR_043325.1
Bacillus sporothermodurans strain M215 16S ribosomal RNA gene, partial sequence	1777	1777	99%	0.0	95.16%	NR_118833.1
Bacillus vini strain LAM0415 16S ribosomal RNA, partial sequence	1775	1775	99%	0.0	95.16%	NR_146819.1
Bacillus oleronius strain DSM 9356 16S ribosomal RNA, partial sequence	1768	1768	99%	0.0	94.99%	NR_119157.1

Bacillus peanut GL9

CCATCTCTGTCACCTTCGGCGGCTGGCTCCAAAAGGTTACCTCACCGACTTCGGGTGTTACAAACTCT
CGTGGTGTGACGGGCGGTGTGTACAAGGCCCGGGAACGTATTCACCGCGGCATGCTGATCCGCGATTA
CTAGCGATTCCAGCTTCATGCAGGCGAGTTGCAGCCTGCAATCCGAACTGAGAATGGTTTTATGGGATT
GGCTTAACCTCGCGGTCTCGCAGCCCTTTGTACCATCCATTGTAGCACGTGTGTAGCCCAGGTCATAAG
GGGCATGATGATTTGACGTCATCCCCACCTTCCTCCGGTTTGTCACCGGCAGTCACCTTAGAGTGCCCA
ACTGAATGCTGGCAACTAAGGTCAAGGGTTGCGCTCGTTGCGGGACTTAACCCAACATCTCACGACAC
GAGCTGACGACAACCATGCACCACCTGTCACTCTGTCCCCCGAAGGGGAAGGCCCTATCTCTAGGGA
GGTCAGAGGATGTCAAGACCTGGTAAGGTTCTTCGCGTTGCTTCGAATTAAACCACATGCTCCACCGC
TTGTGCGGGCCCCCGTCAATTCCTTTGAGTTTCAACCTTGCGGTCGTACTCCCCAGGCGGAGTGCTTA
ATGCGTTAGCTGCAGCACTAAAGGGCGGAAACCCTCTAACACTTAGCACTCATCGTTTACGGCGTGGA
CTACCAGGGTATCTAATCCTGTTTGCTCCCCACGCTTTCGCGCCTCAGCGTCAGTTACAGACCAGAGAG
TCGCCTTCGCCACTGGTGTTCCTCCACATCTCTACGCATTTCACCGCTACACGTGGAATTCCACTCTCC
TCTTCTGCACTCAAGTCTTCCAGTTTCCAATGACCCTCCACGGTTGAGCCGTGGGCTTTCACATCAGAC
TTAAAAGACCGCCTGCGCGCGCTTTACGCCCAATAATTCCAGGACAACGCTTGCCACCTACGTATTACC
GCGGCTGCTGGCACGTAGTTAGCCGTGCTTTCTGGTTAGGTACCGTCAAGGTACCGCCCTATTCGAAC
GATACTTGCTCTCCTAACAACAGAGTTTACGATCAGAGATCTTTCTCACTCACGCGGCGTTGCTCGTCA
GACTTTCGTCATGCGATGATCCTACTGCTGCCTCCGTAGAGTTAGGCCGTGTCTTCAG

Bacillus ginsengihumi strain Gsoil 114 16S ribosomal RNA, partial sequence	1939	1939	99%	0.0	97.76%	NR_041378.1
Bacillus shackletonii strain LMG 18435 16S ribosomal RNA, partial sequence	1891	1891	99%	0.0	96.82%	NR_025373.1
Bacillus acidicola strain 105-2 16S ribosomal RNA, partial sequence	1862	1862	99%	0.0	96.30%	NR_041942.1
Bacillus camelliae strain 7578-1 16S ribosomal RNA, partial sequence	1840	1840	99%	0.0	95.96%	NR_159341.1
Bacillus sporothermodurans strain M215 16S ribosomal RNA, partial sequence	1829	1829	99%	0.0	95.79%	NR_118833.1
Bacillus sporothermodurans strain M215 16S ribosomal RNA, partial sequence	1829	1829	99%	0.0	95.79%	NR_118832.1
Bacillus sporothermodurans strain M215 16S ribosomal RNA, partial sequence	1829	1829	99%	0.0	95.79%	NR_026010.1
Bacillus isabeliae strain CVS-8 16S ribosomal RNA, partial sequence	1828	1828	99%	0.0	95.78%	NR_042619.1
Bacillus dakarensis strain Marseille-P3515 16S ribosomal RNA, partial sequence	1816	1816	99%	0.0	95.45%	NR_147382.1
Bacillus gottheilii strain WCC 4585 16S ribosomal RNA, partial sequence	1815	1815	99%	0.0	95.36%	NR_108491.1
Bacillus vini strain LAM0415 16S ribosomal RNA, partial sequence	1810	1810	98%	0.0	95.75%	NR_146819.1
Bacillus oleronius strain ATCC 700005 16S ribosomal RNA, partial sequence	1806	1806	99%	0.0	95.45%	NR_043325.1
Bacillus haikouensis strain C-89 16S ribosomal RNA, partial sequence	1799	1799	99%	0.0	95.09%	NR_148273.1
Bacillus purgationiresistens strain DS22 16S ribosomal RNA, partial sequence	1798	1798	99%	0.0	95.10%	NR_108492.1

Bacillus peanut GL10

GCTCCCGTGCGGCGTGCTATACATGCAAGTCGAGCGAATTGGATTAAGAGCTTGCTCTTAGTAGCAGTT
AGCGGCGGACGGGTGAGTAACACGTGGGTAACCTGCCCAGTAAGACTGGGATAACTCCGGGAAACCG
GGGCTAATACCGGATAACTTTTTGAACCGCATGGTTCGAAATTGAAAGGTGGCTTCGGCTGTCACTTAT
GGATGGACCCGCGTCGCATTAGCTAGTTGGTGAGGTAACGGCTCACCAAGGCAACGATGCGTAGCCG
ACCTGAGAGGGTGATCGGCCACATTGGGACTGAGACACGGCCCAAACTCCTACGGGAGGCAGCAGTA
GGGAATCTTCCGCAATGGACGAAAGTCTGACGGAGCAACGCCGCGTGAGTGATGAAGGCTTTCGGAT
CGTAAAACTCTGTTGTTAGGGAAGAACAAGTGCTAGTTGAATAAGGTGGCACCTTGACGGTACCTAAC
CAGAAAGCCACGGCTAACTACGTGCCAGCAGCCGCGGTAATACGTAGGTGGCAAGCGTTATCCGGAAT
TATTGGGCGTAAAGCGCGCGCAGGTGGTTTCTTAAGTCTGATGTGAAAGCCCACGGCTCAACCGTGGA
GGGTCATTGGAAACTGGAAGACTTGAGTGCAGAAGAGGAGAGTGGAATTCCATGTGTAGCGGTGAAA
TGCGTAGAGATGTGGAGGAACACCAGTGGCGAAGGCGACTTTCTGGTCTGTAACTGACACTGAGGCG
CGAAAGCGTGGGGAGCAAACAGGATTAGATACCCTGGTAGTCCACGCCGTAAACGATGAGTGCTAAG
TGTTAGAGGGTTTCCGCCCTTTAGTGCTGAAGCTAACGCATTAAGCACTCCGCCTGGGGAGTACGGCC
GCAAGGCTGAAACTCAAAGGAATTGACGGGGGCCCGCACAAGCGGTGGAGCATGTGGTTTATTCGAA
GCAACGCGAGACTTACCAGTCTTGACATCTCTGACATCCTAGAGATAGGCTCTCCTCGGAGCACAGTA
TCACGTGGTGCTCAGTGTGTCGTCACCTCTTGTGCTCGAGAATATTTGCTTAATTCCGCACACGAGCAA
CCTTGATACTTAGTTTGCCATCAGCTACATGGAACTCTAAAGTGACTGTCGCGTTACCAACCCGAGAAG
GTGTGAATGTAGCTCTCACT

Bacillus paramycoides strain MCCC 1A04098 16S ribosomal RNA, partial sequence	1755	1755	97%	0.0	94.25%	NR_157734.1
Bacillus tropicus strain MCCC 1A01406 16S ribosomal RNA, partial sequence	1749	1749	97%	0.0	94.17%	NR_157736.1
Bacillus nitratireducens strain MCCC 1A00732 16S ribosomal RNA, partial sequence	1749	1749	97%	0.0	94.17%	NR_157732.1
Bacillus luti strain MCCC 1A00359 16S ribosomal RNA, partial sequence	1749	1749	97%	0.0	94.17%	NR_157730.1
Bacillus albus strain MCCC 1A02146 16S ribosomal RNA, partial sequence	1749	1749	97%	0.0	94.17%	NR_157729.1
Bacillus cereus strain ATCC 14579 16S ribosomal RNA (rrnA), partial sequence	1744	1744	97%	0.0	94.09%	NR_074540.1
Bacillus cereus strain JCM 2152 16S ribosomal RNA gene, partial sequence	1744	1744	97%	0.0	94.09%	NR_113266.1
Bacillus cereus strain CCM 2010 16S ribosomal RNA, partial sequence	1744	1744	97%	0.0	94.09%	NR_115714.1
Bacillus cereus strain NBRC 15305 16S ribosomal RNA gene, partial sequence	1744	1744	97%	0.0	94.09%	NR_112630.1
Bacillus cereus strain ATCC 14579 16S ribosomal RNA gene, partial sequence	1744	1744	97%	0.0	94.09%	NR_114582.1

Bacillus peanut GL11

ACGTATCTGTCACCTTAGGCGGCTGGCTCCAAAAGGTTCCTCCCCCAACTTCGGGGGTTCCAAACTCT
CGGGGGGGGACGGGGGGGGTGTCCAAGGCCCGGGAACGTTTTCCCCGCGGCTGGTTGATCCGCGATT
CCTAGCGATTCCGGTTTCTGGTAGGCAAGTTGCACCCTACAATCCGAACTGAAAAGGGTTTTTGGAGA
TTGCTTCCCCCTCGCGGTCTTGCACCTCTTTGTACCTCCCTTTGTAGCACGGGGGTAGCCCAGGTCATA
AGGGGATGGATGATTTGACGTCTTCCCCCCCTTCCTCCGGTTGGTCCCCGGCAGTCCCCTTAAAGGGC
CCAATTAATGGAGGGCAACTAAGATCAAGGGTGGCGCTCGTGGCGGGATTTAACCCACCTTCTCACAA
CACAAGTTGACAACAACCTTGCACCACCGGTCATTCTGCCCCCGAAGGGGAAGGCCTTTTTCTTGGGT
TTTCAAAGGATGTCAAAACCTGGGAAGGGTCTTTCCGTTGCTTTCAATTTAACCCCATGGTCCACCCCT
TGTGCGGGCCCCCCTCAATTTCTTTTAATTTCAGACTTGGGGGCGTACTCCCCAGGGGGGGTGCTTAA
AGCGTTTACTTCAGAACTTAAGGGGGGAAACCCCCTAAAACTTAGCACTCATCGTTTTCGGCGTGGAC
TACCAGGGGATCTAATCCTGTTTGCTCCCCACGGTTTCGGGCCTCGAGTTAGTTACAGACCCAAAAGT
CGCCCTTTGCCACTGGGGTTCCCCCCTATCTTCTACCCATTTCACCGCTCACATGGAAATTCACTTTCCT
CTTTTGGACTCAAGCTCTCAGTTCCAAAGACCCTCCACAGGTTGAGCCGGGGGCTTTCACATCAGAAA
TTAAAAAGACCCCCTGCGCGCCGCCTTACGCCAATAATTTCCGATAAACGCTTGGCCACCCTACGTATT
TACCGCGCGCTGCCTGGCACGTAGTTAAGCGGTGGCCTTTCTAGTAGTTACGGTCAGGTAACCAGCCT
TATTCGCCACAGGTACTTGATTCTCCTTAACACACGAGAGTTTACGATCCGGAAGACTTCACTCACTCA
GCGGCGTGCTCGTCGACTCGTCATTGCGAGAATTCATCGCTGCTCGTAGACTGCCGTTCAGTTCAATGT
GACGATC

Bacillus bingmayongensis strain FJAT-13831 16S ribosomal RNA, partial sequence	1122	1122	95%	0.0	84.32%	NR_148248.1
Bacillus pseudomycoides strain NBRC 101232 16S ribosomal RNA, partial sequence	1118	1118	95%	0.0	84.32%	NR_113991.1
Bacillus wiedmannii strain FSL W8-0169 16S ribosomal RNA, partial sequence	1113	1113	95%	0.0	84.23%	NR_152692.1
Bacillus pacificus strain MCCC 1A06182 16S ribosomal RNA, partial sequence	1113	1113	95%	0.0	84.23%	NR_157733.1
Bacillus mobilis strain MCCC 1A05942 16S ribosomal RNA, partial sequence	1113	1113	95%	0.0	84.23%	NR_157731.1
Bacillus paranthracis strain MCCC 1A00395 16S ribosomal RNA, partial sequence	1113	1113	95%	0.0	84.23%	NR_157728.1
Bacillus proteolyticus strain MCCC 1A00365 16S ribosomal RNA, partial sequence	1113	1113	95%	0.0	84.23%	NR_157735.1
Bacillus toyonensis strain BCT-7112 16S ribosomal RNA, partial sequence	1113	1113	95%	0.0	84.23%	NR_121761.1
Bacillus cereus ATCC 14579 16S ribosomal RNA (rrnA), partial sequence	1113	1113	95%	0.0	84.23%	NR_074540.1
Bacillus cereus strain JCM 2152 16S ribosomal RNA, partial sequence	1113	1113	95%	0.0	84.23%	NR_113266.1
Bacillus thuringiensis strain NBRC 101235 16S ribosomal RNA, partial sequence	1113	1113	95%	0.0	84.23%	NR_112780.1

Bacillus peanut GL12

ACGCCGGCGCGTGCTATACATGCAAGTCGAGCGAATGGATTAAGAGCTTGCTCTTATGAAGTTAGCGG
CGGACGGGTGAGTAACACGTGGGTAACCTGCCCATAAGACTGGGATAACTCCGGGAAACCGGGGCTA
ATACCGGATAACATTTTGAACTGCATGGTTCGAAATTGAAAGGCGGCTTCGGCTGTCACTTATGGATGG
ACCCGCGTCGCATTAGCTAGTTGGTGAGGTAACGGCTCACCAAGGCAACGATGCGTAGCCGACCTGA
GAGGGTGATCGGCCACACTGGGACTGAGACACGGCCCAGACTCCTACGGGAGGCAGCAGTAGGGAA
TCTTCCGCAATGGACGAAAGTCTGACGGAGCAACGCCGCGTGAGTGATGAAGGCTTTCGGGTCGTAA
AACTCTGTTGTTAGGGAAGAACAAGTGCTAGTTGAATAAGCTGGCACCTTGACGGTACCTAACCAGAA
AGCCACGGCTAACTACGTGCCAGCAGCCGCGGTAATACGTAGGTGGCAAGCGTTATCCGGAATTATTG
GGCGTAAAGCGCGCGCAGGTGGTTTCTTAAGTCTGATGTGAAAGCCCACGGCTCAACCGTGGAGGGT
CATTGGAAACTGGGAGACTTGAGTGCAGAAGAGGAAAGTGGAATTCCATGTGTAGCGGTGAAATGCG
TAGAGATATGGAGGAACACCAGTGGCGAAGGCGACTTTCTGGTCTGTAACTGACACTGAGGCGCGAA
AGCGTGGGGAGCAAACAGGATTAGATACCCTGGTAGTCCACGCCGTAAACGATGAGTGCTAAGTGTTA
GAGGGTTTCCGCCCTTTAGTGCTGAAGTTAACGCATTAAGCACTCCGCCTGGGGAGTACGGCCGCAAG
GCTGAAACTCAAAGGAATTGACGGGGGCCCGCACAGCGGTGGAGCATGTGGTTTAATTCGAAGCAAC
GCGAAGACCTTACCATGTCTTGACATCGCTCTGAAACCCTAGAGATAGGGCTTCTCCTTCGGGAGCAG
AGTGACAGGTGTTGCATGGTGTCGTCAGCTCTGTCTGAGAATGTTGGGTTAAGTCCCCACCACCCCAA
CCCTTGGATCTTAGTGTGCCATCATTA

Bacillus proteolyticus strain MCCC 1A00365 16S ribosomal RNA, partial sequence	1934	1934	100%	0.0	98.12%	NR_157735.1
Bacillus wiedmannii strain FSL W8-0169 16S ribosomal RNA, partial sequence	1934	1934	100%	0.0	98.12%	NR_152692.1
Bacillus tropicus strain MCCC 1A01406 16S ribosomal RNA, partial sequence	1929	1929	100%	0.0	98.03%	NR_157736.1
Bacillus nitratireducens strain MCCC 1A00732 16S ribosomal RNA, partial sequence	1929	1929	100%	0.0	98.03%	NR_157732.1
Bacillus luti strain MCCC 1A00359 16S ribosomal RNA, partial sequence	1929	1929	100%	0.0	98.03%	NR_157730.1
Bacillus albus strain MCCC 1A02146 16S ribosomal RNA, partial sequence	1929	1929	100%	0.0	98.03%	NR_157729.1
Bacillus cereus strain ATCC 14579 16S ribosomal RNA (rrnA), partial sequence	1929	1929	100%	0.0	98.03%	NR_074540.1
Bacillus cereus strain JCM 2152 16S ribosomal RNA gene, partial sequence	1929	1929	100%	0.0	98.03%	NR_113266.1
Bacillus cereus strain CCM 2010 16S ribosomal RNA, partial sequence	1929	1929	100%	0.0	98.03%	NR_115714.1
Bacillus cereus strain NBRC 15305 16S ribosomal RNA gene, partial sequence	1929	1929	100%	0.0	98.03%	NR_112630.1
Bacillus cereus strain ATCC 14579 16S ribosomal RNA gene, partial sequence	1929	1929	100%	0.0	98.03%	NR_114582.1

Bacillus peanut GL13

TCGCGGGGGGGTGCTATACATGCAGTCGAGCGAATGGATTAAGAGCTTGCTCTTATGAAGTTAGCGGC
GGACGGGTGAGTAACACGTGGGTAACCTGCCCATAAGACTGGGATAACTCCGGGAAACCGGGGCTAA
TACCGGATAACATTTTGAACTGCATGGTTCGAAATTGAAAGGCGGCTTCGGCTGTCACTTATGGATGGA
CCCGCGTCGCATTAGCTAGTTGGTGAGGTAACGGCTCACCAAGGCAACGATGCGTAGCCGACCTGAG
AGGGTGATCGGCCACACTGGGACTGAGACACGGCCCAGACTCCTACGGGAGGCAGCAGTAGGGAATC
TTCCGCAATGGACGAAAGTCTGACGGAGCAACGCCGCGTGAGTGATGAAGGCTTTCGGGTCGTAAAA
CTCTGTTGTTAGGGAAGAACAAGTGCTAGTTGAATAAGCTGGCACCTTGACGGTACCTAACCAGAAAG
CCACGGCTAACTACGTGCCAGCAGCCGCGGTAATACGTAGGTGGCAAGCGTTATCCGGAATTATTGGG
CGTAAAGCGCGCGCAGGTGGTTTCTTAAGTCTGATGTGAAAGCCCACGGCTCAACCGTGGAGGGTCA
TTGGAAACTGGGAGACTTGAGTGCAGAAGAGGAAAGTGGAATTCCATGTGTAGCGGTGAAATGCGTA
GAGATATGGAGGAACACCAGTGGCGAAGGCGACTTTCTGGTCTGTAACTGACACTGAGGCGCGAAAG

CGTGGGGAGCAAACAGGATTAGATACCCTGGTAGTCCACGCCGTAAACGATGAGTGCTAAGTGTTAGA
GGGTTTCCGCCCTTTAGTGCTGAAGTTAACGCATTAAGCACTCCGCCTGGGGAGTACGGCCGCAAGGC
TGAAACTTCAAAGGAATTGACGGGGGCCCGCACAAGCGGTGGAGCATGTGGTTTAATTCGAAGCAA
CGCGGAGAACCTTACCAGGTCTTGACATCGCTCTGAAACCCTAGAGATAGGGCTTTCTTCCTTCGGGA
GCAAATTGACAGGTGATTGCATGGTTTGTCGTCAGCTCTTGTCTTGAGATGTTGGGTTAATGTCCCGCA
ACCAGCGCAACCTCTTTGATCCTTAGTTTGTCCTTCA

Bacillus proteolyticus strain MCCC 1A00365 16S ribosomal RNA, partial sequence	1905	1905	99%	0.0	97.76%	NR_157735.1
Bacillus wiedmannii strain FSL W8-0169 16S ribosomal RNA, partial sequence	1905	1905	99%	0.0	97.76%	NR_152692.1
Bacillus tropicus strain MCCC 1A01406 16S ribosomal RNA, partial sequence	1899	1899	99%	0.0	97.67%	NR_157736.1
Bacillus paramycoides strain MCCC 1A04098 16S ribosomal RNA, partial sequence	1899	1899	99%	0.0	97.67%	NR_157734.1
Bacillus nitratireducens strain MCCC 1A00732 16S ribosomal RNA, partial sequence	1899	1899	99%	0.0	97.67%	NR_157732.1
Bacillus luti strain MCCC 1A00359 16S ribosomal RNA, partial sequence	1899	1899	99%	0.0	97.67%	NR_157730.1
Bacillus albus strain MCCC 1A02146 16S ribosomal RNA, partial sequence	1899	1899	99%	0.0	97.67%	NR_157729.1
Bacillus cereus strain ATCC 14579 16S ribosomal RNA (rrnA), partial sequence	1899	1899	99%	0.0	97.67%	NR_074540.1
Bacillus cereus strain JCM 2152 16S ribosomal RNA gene, partial sequence	1899	1899	99%	0.0	97.67%	NR_113266.1
Bacillus cereus strain CCM 2010 16S ribosomal RNA, partial sequence	1899	1899	99%	0.0	97.67%	NR_115714.1
Bacillus cereus strain NBRC 15305 16S ribosomal RNA gene, partial sequence	1899	1899	99%	0.0	97.67%	NR_112630.1

Bacillus peanut GL14

CTCGCATGCGCTACTACACATGCAGTCGAGCGGTAACTTGTAGTTCCTTGCTGGTTAGCGGGGGACAC
GTGGGTAACACAGGAAAGTCCGGGACACCGCACGGTGGTAGGGGATAAAAACGGAGCCAGTCATAAT
ACCCCATTTGGTCGCATGACCAAATCGGGGGACCATCAGCCCTCTTGCCCTCAGATGTGGCCCCATGG
CATTATCTCTAGGGGGGGGTGACGGGTCTCCCAGGCGACAATACGCAGCTGGGATGAGAGGAGGAGC
TCCCACACTGTGGCTGAGACACGCTCCCCACTCCCCCAGGGGGGGGAAGTGGGGAATATTGTCCAAT
GGGGACAAGCCTGAGGCAGCCCTGCCGCGTGTGTGAGAAAAGCCTTCTGGTAGTATAGTACTTTCTTG
CCGGGGGAAAGCGCTTACGATAATACCTGCGCTCAATGAGATTACCCCTGAAAAAAAAGCCCCGTAAC
TCCACGCGCCCACCCCCGCTAATACACATGGGGCAAACGTTTTTCCGGATTACTGTGCGTATAGCGCGC
GCACGCGGTTTGTTAAGTCTCACGCGAAATCCCTCGGATCTACCTGTCAACTGCCTCTGAAACTGGCA
AGCTTTAATTGCGGAAAAGGAGGTAGAATTTCCTGTGGTACGGTGAAATTGCTAAAAATCTAGAAGAA
TCACCGTTGCCAAAGGCGACTCTCTGGACCTGTACTGGACGCTCGAGGGCGAAAAGCGCGGGGGAGC
AACACGGATTAGATACTCGTGGTAGTTCCACGCCGTAAACTATGTAGTGCTTGCATGTTGTGCGCTTTG
AGACGTGTTGTTCCCCGAACTATAGCGCATTAGTCGACTCGCCCTGGGGGATATAGGCTGCGAAGTAC
AGAACACTCATAGAGAATCAGACGGGAGACCGCGCAATACGCAGTGAACATATGTGTTTAAATTTCAT
AAGCACGCGGCAGAAACCGTTTACCGGCTCTTAGCATTCCACGTCAGATCTTCCTAGATGACAATGCT
GTCCCTTTCGGAAACAGAAGAACACGTGGCGTGCTGTGCTTGCCTTCAGTTCCGTTTCGAAATAGTTT
TGTTAAATCCCCTAACCGCCGAACACTTATGCCTATGATTGCGAGCATTCTGGCGTAACCTAGAGGATG
ACTGCAGTATACAACTCGCGAAGAAGGTGGGATTAGACCTGGATTTCTATGCTAAGACCGGTCACACG
TACTCCATTGGTCGATACCAAGGGA

Description	Max score	Total score	Query cover	E value	Ident	Accession
Bacillus paramycoides strain MCCC 1A04098 16S ribosomal RNA, partial sequence	1704	1704	98%	0.0	93.69%	NR_157734.1
Bacillus tropicus strain MCCC 1A01406 16S ribosomal RNA, partial sequence	1700	1700	98%	0.0	93.60%	NR_157736.1
Bacillus nitratireducens strain MCCC 1A00732 16S ribosomal RNA, partial sequence	1700	1700	98%	0.0	93.60%	NR_157732.1
Bacillus luti strain MCCC 1A00359 16S ribosomal RNA, partial sequence	1700	1700	98%	0.0	93.60%	NR_157730.1
Bacillus albus strain MCCC 1A02146 16S ribosomal RNA, partial sequence	1700	1700	98%	0.0	93.60%	NR_157729.1
Bacillus cereus strain ATCC 14579 16S ribosomal RNA (rrnA), partial sequence	1695	1695	98%	0.0	93.52%	NR_074540.1
Bacillus cereus strain JCM 2152 16S ribosomal RNA gene, partial sequence	1695	1695	98%	0.0	93.52%	NR_113266.1
Bacillus cereus strain CCM 2010 16S ribosomal RNA, partial sequence	1695	1695	98%	0.0	93.52%	NR_115714.1
Bacillus cereus strain NBRC 15305 16S ribosomal RNA gene, partial sequence	1695	1695	98%	0.0	93.52%	NR_112630.1

paenibacillus GL1-3

CAATGAATCCACCTTGGAAGCTGCCTCCTAGGTTAATATACCTACTTTTTTTGTGTCCAATCCTCTGGTG
GGGCGAGGGGCGGGGTGAACGACACCCGAACAAATTATTCACCGCGTTCTGCTGATCCATTATTACTA
GCAATTCCGACTTCATGCAGGCGATTTGCAGCCTGCAATCCAAACTGAGACCGACTTTGATAGGATTGT

CTCCTCCTCAGGGTTTCTCTTCCCGTTGTATCCATTGTTGTCGTGGTGTGCCCTGGAGGTAAGGGGGAT
GATGACTTTTTGTCATCCCCCCCTTCTTCCTCCTGTTTGTCGGCGGTCTCCTTCGAGTTCCCCCCCCGAA
AGGTGGTGACAAATGATAACGGGGGCTCTCCTCGTTGGACTACTTACCCCATCTTCTCACAACACCAG
ATGACAACCATGATGCACCTGTTGTATCCTTCCCCAAGGAACAAACCCCTCTCTGAAAAATTCTCAGA
ATGACAACACCAGGTAAGGTTCTTCTCCTTGCATCTAATTAAACCACATGCTCCACCGCTTGTTGGGGC
CCCCCCCAATTCTTTTTATTTTTATCCTTGCGGCCATACTCCTCCCGCGGCCGAATTATTACGGTATCTCC
TTCAGCAACACCTCTATGGCACACCCTCACACTTAACATCCTTTATTGACGGGATGAACTAGGAAGCTA
TTCATGCTTGTTCCCCCCCCTTTCTCACCCGCCCCACCGTCATCTACACCGCGGAAAGTCTCCCCCCCC
CACTGGTGTCCCTATATATCTCTATTTATCTGCCCCCCTCCGCAAGTAATTCCACCTTCTCCTCATACTGC
ACTCAAGCTATGCACATACGCATGTTGACACGAAGATCACCGTCCGATGTTATCATCTGACAGTACTTA
AAACGCCCTGCCTTGCCGCGTTCATTACGACCCAATTACTTACTGGACGACTTGCTAGCCTCCGTACGT
ACTACGCTGCTGCTAGCGGAGTAGTTCAGCGCTGCCTTCTTGTCCGCAGCAACGATCGACCTTACGAT
CATGATCATCACGTCAGTCTCCTCGCCTGAAGTACGATTCATACGATACGACATCTTCCATACCTCAGCA
TGCTGCATCAGGCTAGCTTCGATCGTGCCAAATGACTACTGCTGCTGCGTCGAGCTGTACGACCTGATA
CATCTGAAGGTG

Paenibacillus gorillae strain G1 16S ribosomal RNA, partial sequence	270	270	78%	1e-71	72.80%	NR_136879.1
Paenibacillus catalpae strain D75 16S ribosomal RNA gene, partial sequence	270	270	78%	1e-71	72.86%	NR_118012.1
Paenibacillus glycanilyticus strain DS-1 16S ribosomal RNA gene, partial sequence	270	270	78%	1e-71	72.86%	NR_024759.1
Paenibacillus glycanilyticus strain NBRC 16618 16S ribosomal RNA gene, partial sequence	268	268	78%	4e-71	72.76%	NR_113853.1
Paenibacillus radicis strain 694 16S ribosomal RNA, partial sequence	265	265	78%	5e-70	72.70%	NR_148658.1
Paenibacillus lupini strain RLAHU15 16S ribosomal RNA, partial sequence	265	265	78%	5e-70	72.75%	NR_134115.1
Paenibacillus prosopidis strain PW21 16S ribosomal RNA gene, partial sequence	263	263	44%	2e-69	76.15%	NR_116828.1
Paenibacillus castaneae strain Ch-32 16S ribosomal RNA, partial sequence	263	263	44%	2e-69	76.19%	NR_044403.1
Paenibacillus alkaliterrae strain KSL-134 16S ribosomal RNA gene, partial sequence	259	259	44%	2e-68	75.92%	NR_043293.1

Kosakonia GL1

GGCTTGCGCAGCTACACATGCAGTCGAACGGTAGCACAGAGAGCTTGCTCTCTGGTGACGAGTGGGG
GACGGGTGAGTAATGTCTGGGAAACTGCCTGATGGAGGGGGATAACTACTGGGAACGGTAGCTAATAC
CGCATAACGTCTCAAGAGCAAAGAGGGGGACCTTCGGGCCTCTTGCCATCTCATGTGCCCAGATGGGA
TTAGCTAGTAGGCGGGGTAATGGCCCACCTAGGCGACAATCCCTAGCTGGGCTGAGAGGATGACCAGC
CACACTGGAACTGAGACACGGTCCAGACTCCTACGGGAGGCAGCAGTGGGGAATATTGCACAATGGG
CGCAAGCCTGATGCACCCATGCCGCGTGTGTGAAAAAAGCCTTCTGGTTGTAAAGAACTTTCAGCGG
GGAGGAAGGGGGTGCGGTTAATAACCCCGCCGATTGACGTTACCCCCACAAAAAGCACCGGCTAACT
CCCTGCCAGCAGCCGCGGTAATACGGAGGGGGCAAGCGTTAATCGGAATTACTGGGCGTAAAGCGCA
CGCACGCGGTCTGTCAAGTCGGATGTGAAATCCCCGGGCTCAACCTGGGAACTGCATTCTAAACTGGG
AGGCTGGAGTCTCGTAGAGGGAGGTAGAATTCCAGGTGTAGCGCTGAAATGCGTAGAGATCTGGAGG
AATACCGGTGGCGAAAGCGGCCTCCTGGACAAAAACTGACACTCACGTGCGAAAGCGTGGGGAGCA
AACACGATTAGATACACTGGGAGTCCACGCCGTAAACGATGTCTATATGTAGGTTGTGCCCTTGAGGCG
TGTGTTCCCGAGCTAACACGTTAAATCTACCGCCTGGGGAGTACAGCCGCAAGGTTAAAACTCACATG
AATTGTCAGGGGGCCCGCACAAGCGCTGGAGCATGTGGTTTAATTCTATGCAACACGAAGAACCTCAC
CTGGTCTTGTCATCCACAGAACCTTCTCGCAGATGTGGCAGTGTCCTTCTCGAACTGTGAGACACGTG
CTGCTATTGGCTGTCGTCAGCTCGTCGTTGTGAAATGTTGTGGTTAAGATCCCGCAACGAGCAGCAAC
ACCCTTATTACCTTTTGTTTGTCCAGCGGTTAGGTCGGCGGAACTACCTCAAGGAGAACTGCGTCGCT
GGATTAATACCTTGTAGA

Kosakonia radicincitans strain DSM 16656 16S ribosomal RNA gene, partial sequence	1585	1585	94%	0.0	92.77%	NR_117704.1
Kosakonia oryzae strain Ola 51 16S ribosomal RNA gene, partial sequence	1570	1570	94%	0.0	92.72%	NR_116033.1
Enterobacter oryziphilus strain REICA_142 16S ribosomal RNA gene, partial sequence	1537	1537	93%	0.0	92.39%	NR_125587.1
Enterobacter cloacae subsp. dissolvens strain ATCC 23373 16S ribosomal RNA gene, partial sequence	1537	1537	94%	0.0	91.97%	NR_118011.1
Kosakonia arachidis strain Ah-143 16S ribosomal RNA gene, partial sequence	1537	1537	94%	0.0	92.06%	NR_116403.1
Enterobacter cloacae subsp. dissolvens strain LMG 2683 16S ribosomal RNA gene, partial sequence	1533	1533	94%	0.0	91.88%	NR_044978.1
Klebsiella pneumoniae strain ATCC 13883 16S ribosomal RNA gene, partial sequence	1530	1530	94%	0.0	91.88%	NR_114506.1
Enterobacter cloacae strain DSM 30054 16S ribosomal RNA gene, partial sequence	1528	1528	94%	0.0	92.01%	NR_117679.1
Enterobacter cloacae strain NBRC 13535 16S ribosomal RNA gene, partial sequence	1528	1528	94%	0.0	92.01%	NR_113615.1

Kosakonia GL2

GCACTGATCCAAGTGGTAGCGCCCTCCCGAAGGTTAAGCTACCTACTTCTTTTGCAACCCACTCCCATG
GTGTGACGGGCGGTGTGTACAAGGCCCGGGAACGTATTCACCGTGGCATTCTGATTCACGATTACTAG
CGATTCCGACTTCATGGAGTCGAGTTGCAGACTCCAATCCGGACTACGACGCACTTTATGAGGTCCGC
TTGCTCTCGCGAGGTCGCTTCTCTTTGTATGCGCCATTGTAGCACGTGTGTAGCCCTGGTCGTAAGGGC
CATGATGACTTGACGTCATCCCCACCTTCCTCCAGTTTATCACTGGCAGTCTCCTTTGAGTTCCCGGCC
TGACCGCTGGCAACAAAGGATAAGGGTTGCGCTCGTTGCGGGACTTAACCCAACATTTCACAACACG
AGCTGACGACAGCCATGCAGCACCTGTCTCACAGTTCCCGAAGGCACCCTGCCATCTCTGCAAAGTTC
TGTGGATGTCAAGACCAGGTAAGGTTCTTCGCGTTGCATCGAATTAAACCACATGCTCCACCGCTTGT
GCGGGCCCCCGTCAATTCATTTGAGTTTTAACCTTGCGGCCGTACTCCCCAGGCGGTCGATTTAACGCG
TTAGCTCCGGAAGCCACGCCTCAAGGGCACAACCTCCAAATCGACATCGTTTACAGCGTGGACTACCA
GGGTATCTAATCCTGTTTGCTCCCCACGCTTTCGCACCTGAGCGTCAGTCTTCGTCCAGGAGGCCGCCT
TCGCCACCGGTATTCCTCCAGATCTCTACGCATTTCACCGCTACACCTGGAATTCTACCTCCCTCTACGA
GACTCCAGCCTGCCAGTTTCGAATGCAGTTCCCAGGTTGAGCCCGGGGATTTCACATCCGACTTGACA
GACCGCCTGCGTGCGCTTTACGCCCAGTAATTCCGATTAACGCTTGCACCCTCCGTATTACCGCGGCTG
CTGGCACGGGAGTTAGCCGGTGCTTCTTCTGCGGTAACGTCAATCAGCTAGCGGTTATTAACCAGTACT
GCCTTCTCCCGCTGAAAGTGCTTTTACAACCGACGCTTCTCATACACGCGGCATGCTGCATCAAGCTGC
GCCATGTGCAATATCCCCACTGCTGCCTCCCGTAGGAGTCTTGGACGGTGGTCTCTCAG

	Score	Score	Cover	value	Ident	
Kosakonia radicincitans DSM 16656 16S ribosomal RNA, partial sequence	1867	1867	98%	0.0	96.88%	NR_117704.1
Kosakonia oryzae strain Ola 51 16S ribosomal RNA, partial sequence	1867	1867	98%	0.0	96.88%	NR_116033.1
Kosakonia oryzendophytica strain REICA_082 16S ribosomal RNA, partial sequence	1859	1859	98%	0.0	96.95%	NR_125586.1
Kosakonia pseudosacchari strain JM-387 16S ribosomal RNA, partial sequence	1852	1852	98%	0.0	96.45%	NR_135211.1
Kosakonia arachidis strain Ah-143 16S ribosomal RNA, partial sequence	1849	1849	98%	0.0	96.72%	NR_116403.1
Kosakonia oryziphila strain REICA_142 16S ribosomal RNA, partial sequence	1846	1846	98%	0.0	96.77%	NR_125587.1
Enterobacter cloacae strain DSM 30054 16S ribosomal RNA, partial sequence	1832	1832	98%	0.0	96.27%	NR_117679.1
Enterobacter cloacae strain NBRC 13535 16S ribosomal RNA, partial sequence	1832	1832	98%	0.0	96.27%	NR_113615.1
Enterobacter cloacae strain 279-56 16S ribosomal RNA, partial sequence	1832	1832	98%	0.0	96.27%	NR_028912.1
Kosakonia sacchari strain SP1 16S ribosomal RNA, partial sequence	1831	1831	98%	0.0	96.28%	NR_118333.1
Enterobacter cloacae subsp. dissolvens strain ATCC 23373 16S ribosomal RNA, partial sequence	1824	1824	98%	0.0	96.11%	NR_118011.1
Enterobacter cloacae subsp. dissolvens strain LMG 2683 16S ribosomal RNA, partial sequence	1824	1824	98%	0.0	96.10%	NR_044978.1
Salmonella enterica subsp. enterica strain LT2 16S ribosomal RNA, partial sequence	1819	1819	98%	0.0	96.01%	NR_074910.1

Kosakonia GL3

CCCCTTGTATACAAAGTTGCGTAGCGCCCTCCCGAAGGTTAAGCTACCTACTTCTTTTGCAACCCACTC
CCATGGTGTGACGGGCGGTGTGTACAAGGCCCGGGAACGTATTCACCGTGGCATTCTGATCCACGATT
ACTAGCGATTCCGACTTCATGGAGGCGAGTTGCAGACTCCAATCCGGACTACGACGGACTTTATGAGG
TCGGCTTGCTCTCGCGAGGTCGCTTCTCTTTGTATGCTCCATTGTAGCACGTGTGTAGCCCTGGTCGTA
AGGGGCATGATGACTTGACGTCATCCCCACCTTCCTCCAGTTTATCACTGGCAGTCTCCTTTGAGTTCC
CGGCCGAACCGCTGGCAACAAAGGATAAGGGTTGCGCTCGTTGCGGGACTTAACCCAACATTTCACA
ACACGAGTTGACAACAGCCATGCAGCACCTGTCTCACAGTTCCCGAAGGCACATTCCCATCTCTGGAA
ACTTCTGTGGATGTCAAGACCAGGTAAGGGTCTTCGCGTTGCATCCAATTAAACCACATGCTCCACCG
CTTGGGCGGGCCCCCGTCAATTCATTTGAGTTTTAACCTTGCGGCCGTACTCCCCAGGGGGTCGATTTA

ACGCGTTAGCTCCGGAAGCCACGCCTCAAGGGCACAACCTCCAAATCGACATCGTTTACAGCGTGGA
CTACCAGGGGATCTAATCCTGTGTGCTCCCCACGCTTTCGCACCTGAGCGTCAGTCTTCGTCCAGGAA
GACGCCTTCTCCACCGGGATTCCTCCACATCTCTACACATTTCACCGCTACACCTGGAATACTACCCTC
CCTCTACTAGACTCCAGCCTGCCAGTTTCGAATGCACTCCCCAGGTGAGACCCGGGGATTTCACATCC
GACTTGACAGAGCGCCTGCGTGCGCTTTACGCCCAGAAATTCCGATGAACGCTTGCGCCCCTCCGTAT
TACCGCGGCTGCTGGCACGGAGATAGACCGGTGCTTCTTCTGCGGAGGAACGTCTACTGCTGCCGCT
ATTATACCGACAACATCCTTGCTTCCTCCGCTGAAAAGTACATTTACTACCAGCAGGACTTTCTTTCATA
CCACTGCGGGCAGTGCTGCATCAGGCTTGCGTCCATCTGTGGCAGATACCACTGCTGCCTCCCGCTAG
AAGACATGGAACGGGTCTCACATTCTACCCGAATG

Kosakonia oryzendophytica strain REICA_082 16S ribosomal RNA, partial sequence	1646	1646	96%	0.0	92.78%	NR_125586.1
Enterobacter cloacae strain DSM 30054 16S ribosomal RNA, partial sequence	1636	1636	96%	0.0	92.60%	NR_117679.1
Enterobacter cloacae strain NBRC 13535 16S ribosomal RNA, partial sequence	1636	1636	96%	0.0	92.60%	NR_113615.1
Enterobacter cloacae strain 279-56 16S ribosomal RNA, partial sequence	1636	1636	96%	0.0	92.60%	NR_028912.1
Enterobacter cloacae subsp. dissolvens strain LMG 2683 16S ribosomal RNA, partial sequence	1627	1627	96%	0.0	92.43%	NR_044978.1
Salmonella enterica subsp. enterica strain LT2 16S ribosomal RNA, partial sequence	1622	1622	96%	0.0	92.34%	NR_074910.1
Enterobacter cloacae strain ATCC 13047 16S ribosomal RNA, complete sequence	1621	1621	96%	0.0	92.36%	NR_102794.2
Enterobacter cloacae subsp. dissolvens strain ATCC 23373 16S ribosomal RNA, partial sequence	1618	1618	96%	0.0	92.27%	NR_118011.1
Salmonella enterica subsp. enterica strain Ty2 16S ribosomal RNA, partial sequence	1618	1618	96%	0.0	92.25%	NR_074799.1
Salmonella enterica subsp. salamae strain DSM 9220 16S ribosomal RNA, partial sequence	1618	1618	96%	0.0	92.25%	NR_044372.1
Kosakonia oryziphila strain REICA_142 16S ribosomal RNA, partial sequence	1614	1614	96%	0.0	92.34%	NR_125587.1

Enterobacter oryendophyticus GL1

GGGCTTTGCGGCGGCTACACATGCAAGTCGAACGGTATCACAGAGAACTTGGTTTCTTGGGTGACGA
GTGGGACAGGGGAGTAACATGTGGGGGCCCTGCCTGAAGGAGGGGATTCTCCCGGAAAACGGGAGT
TAATACCGATTAATTTCCTCTGCCCAAGGAGGGAGATCTTCAGACCTCTTGCCCTCTCATGTGCCCATA
GGGGATTAGCTAGTTAGTGGGGTAACGGCTCACCTGCTCAACAATGCCTCGATGGTCTGACAAGATGA
CCGGCCACTCTGGAACTGAGACACTGTCCCAACTCCTACGGGAGGCCGGGAGGGGGAATATTGCACT
CTGGGCGCAAGCCTGATGCACCCATGCCGCGTGTATGAAGAAGGCCTTCGGGTTGTAAAGTACTTTCA
CTCTGGAGGAAGGTGTTGAGGATAATAACCGCAACAATTGACGTTCCCCGCAGAACAAGCACCGGAT
AACTCCGCGCCCTACGCCGCGGCAATACCGAGGGTGCAAGCGTTAATCGCAATTACTGGGCTTATAGC
GCACGCAGGGCGTCTGTGCAGTCTTATGTGAAATCCCCGGGCTCAACCTGGTCACTGCATTAGAAACT
TGGAAGATGGAATCTCTTAAATGGAGGTAAAATTCCTGGTGTTCCGCTGAAATGCGTAGAGATCTGGA
AGATTACCAGGAGCAAAAGCGGCCTCCTGGACTATGACTGACGCTCATGAGCGAGAGCGTGGGGAGC
AAACAGGATTAGATACCCTGGTTGCCCACGCTGTACACGATGTCGATTTTGAGTGTGTGCCCTTGAAG
CGTGTCTTCCCGGAGCTACGCGTTAAATCGACTGCCTGGGTCAGTACTGCCGAGAAGGAAAAAACTG
ATATAAACTCACGGGAGTCCACAGAAGCCGGTGCAACATGTGGGTTAATTTCGATGTAACGCGAAGAA
CCTTACCTAGCTTTGACATGCACAGACGTCTCATGAGATGCGAATGTACCCTCTTGTACTCTTGAGAAC
AGTAGCTTGCTAGT

Enterobacter oryzendophyticus strain REICA_082 16S ribosomal RNA gene, partial sequence	861	861	92%	0.0	83.26%	NR_125586.1
Enterobacter cloacae strain DSM 30054 16S ribosomal RNA gene, partial sequence	857	857	93%	0.0	82.89%	NR_117679.1
Enterobacter cloacae strain NBRC 13535 16S ribosomal RNA gene, partial sequence	857	857	93%	0.0	82.89%	NR_113615.1
Enterobacter cloacae strain 279-56 16S ribosomal RNA gene, partial sequence	857	857	93%	0.0	82.89%	NR_028912.1
Enterobacter cloacae strain ATCC 13047 16S ribosomal RNA, complete sequence	854	854	93%	0.0	82.84%	NR_102794.2
Enterobacter cloacae subsp. dissolvens strain ATCC 23373 16S ribosomal RNA gene, partial sequence	850	850	93%	0.0	82.79%	NR_118011.1
Enterobacter cloacae subsp. dissolvens strain LMG 2683 16S ribosomal RNA gene, partial sequence	848	848	93%	0.0	82.68%	NR_044978.1
Enterobacter ludwigii strain EN-119 16S ribosomal RNA, partial sequence	839	839	93%	0.0	82.60%	NR_042349.1
Pantoea agglomerans strain JCM1236 16S ribosomal RNA gene, partial sequence	839	839	93%	0.0	82.60%	NR_111998.1
Enterobacter kobei strain JCM 8580 16S ribosomal RNA gene, partial sequence	830	830	93%	0.0	82.47%	NR_113321.1
Enterobacter kobei strain CIP 105566 16S ribosomal RNA gene, partial sequence	830	830	93%	0.0	82.44%	NR_028993.1

Klebsiella GL1

GGGCATGCGGCAGCTACACATGCAGTCGAGCGGTAGCACAGAGAGCTTGCTCTCGGGTGACGAGCGG
CGGACGGGTGAGTAATGTCTGGGAAACTGCCTGATGGAGGGGGATAACTACTGGAAACGGTAGCTAA

TACCGCATAACGTCGCAAGACCAAAGTGGGGGACCTTCGGGCCTCATGCCATCAGATGTGCCCAGATG
GGATTAGCTAGTAGGTGGGGTAACGGCTCACCTAGGCGACGATCCCTAGCTGGTCTGAGAGGATGACC
AGCCACACTGGAACTGAGACACGGTCCAGACTCCTACGGGAGGCAGCAGTGGGGAATATTGCACAAT
GGGCGCAAGCCTGATGCAGCCATGCCGCGTGTATGAAGAAGGCCTTCGGGTTGTAAAGTACTTTCAGC
GGGGAGGAAGGCGATAAGGTTAATAACCTTGTCGATTGACGTTACCCGCAGAAGAAGCACCGGCTAA
CTCCGTGCCAGCAGCCGCGGTAATACGGAGGGTGCAAGCGTTAATCGGAATTACTGGGCGTAAAGCGC
ACGCAGGCGGTCTGTCAAGTCGGATGTGAAATCCCCGGGCTCAACCTGGGAACTGCATTCGAAACTG
GCAGGCTAGAGTCTTGTAGAGGGGGGTAGAATTCCAGGTGTAGCGGTGAAATGCGTAGAGATCTGGA
GGAATACCGGTGGCGAAGGCGGCCCCCTGGACAAAGACTGACGCTCAGGTGCGAAAGCGTGGGGAG
CAAACAGGATTAGATACCCTGGTAGTCCACGCCGTAAACGATGTCGATTTGGAGGTTGTGCCCTTGAG
GCGTGGCTTCCGGAGCTAACGCGTTAAATCGACCGCCTGGGGAGTACGGCCGCAAGGTTAAAACTCA
AATGAATTGACGGGGGCCCGCACAAGCGGTGGAGCATGTGGTTTAATTCGATGCAACGCGAAGAACC
TTACCTGGTCTTGACATCCACAGAACTTAGCAGAGATGCTTTGGTGCCTTCGGGAACTGTGAGACAGG
TGCTGCATGGCTGTCGTCAGCTCGTGTTGTGAAATGTTGGGTTAAGTCCCGCACGAGCGCACCCTTATC
CTTTGTTGCCAGCGGTTCAGGGCCGGGAACTCAAAGGAGAACTGCCCAGGTG

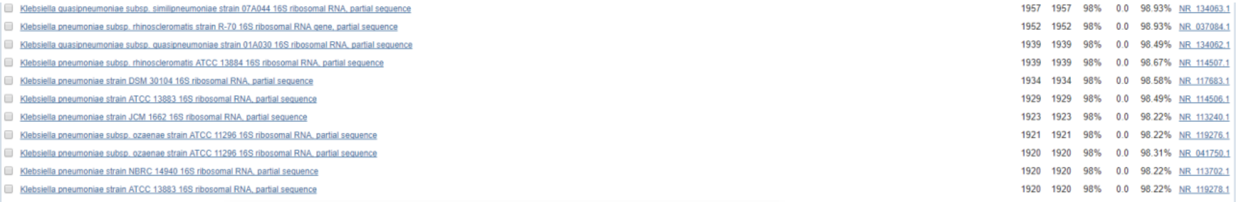

Klebsiella quasipneumoniae subsp. similipneumoniae strain 07A044 16S ribosomal RNA, partial sequence	1957	1957	98%	0.0	98.93%	NR_134063.1
Klebsiella pneumoniae subsp. rhinoscleromatis strain R-70 16S ribosomal RNA gene, partial sequence	1952	1952	98%	0.0	98.93%	NR_037084.1
Klebsiella quasipneumoniae subsp. quasipneumoniae strain 01A030 16S ribosomal RNA, partial sequence	1939	1939	98%	0.0	98.49%	NR_134062.1
Klebsiella pneumoniae subsp. rhinoscleromatis ATCC 13884 16S ribosomal RNA, partial sequence	1939	1939	98%	0.0	98.67%	NR_114507.1
Klebsiella pneumoniae strain DSM 30104 16S ribosomal RNA, partial sequence	1934	1934	98%	0.0	98.58%	NR_117683.1
Klebsiella pneumoniae strain ATCC 13883 16S ribosomal RNA, partial sequence	1929	1929	98%	0.0	98.49%	NR_114506.1
Klebsiella pneumoniae strain JCM 1662 16S ribosomal RNA, partial sequence	1923	1923	98%	0.0	98.22%	NR_113240.1
Klebsiella pneumoniae subsp. ozaenae strain ATCC 11296 16S ribosomal RNA, partial sequence	1921	1921	98%	0.0	98.22%	NR_119276.1
Klebsiella pneumoniae subsp. ozaenae strain ATCC 11296 16S ribosomal RNA, partial sequence	1920	1920	98%	0.0	98.31%	NR_041750.1
Klebsiella pneumoniae strain NBRC 14940 16S ribosomal RNA, partial sequence	1920	1920	98%	0.0	98.22%	NR_113702.1
Klebsiella pneumoniae strain ATCC 13883 16S ribosomal RNA, partial sequence	1920	1920	98%	0.0	98.22%	NR_119278.1

Klebsiella GL2

GCCCACTTGATCCAAGTGGTAGCGCCCTCCCGAAGGTTAAGCTACCTACTTCTTTTGCAACCCACTCCC
ATGGTGTGACGGGCGGTGTGTACAAGGCCCGGGAACGTATTCACCGTAGCATTCTGATCTACGATTACT
AGCGATTCCGACTTCATGGAGTCGAGTTGCAGACTCCAATCCGGACTACGACATACTTTATGAGGTCC
GCTTGCTCTCGCGAGGTCGCTTCTCTTTGTATGCGCCATTGTAGCACGTGTGTAGCCCTGGTCGTAAGG
GCCATGATGACTTGACGTCATCCCCACCTTCCTCCAGTTTATCACTGGCAGTCTCCTTTGAGTTCCCGG
CCTAACCGCTGGCAACAAAGGATAAGGGTTGCGCTCGTTGCGGGACTTAACCCAACATTTCACAACAC
GAGCTGACGACAGCCATGCAGCACCTGTCTCACAGTTCCCGAAGGCACCAAAGCATCTCTGCTAAGT
TCTGTGGATGTCAAGACCAGGTAAGGTTCTTCGCGTTGCATCGAATTAAACCACATGCTCCACCGCTTG
TGCGGGCCCCCGTCAATTCATTTGAGTTTTAACCTTGCGGCCGTACTCCCCAGGCGGTCGATTTAACGC
GTTAGCTCCGGAAGCCACGCCTCAAGGGCACAACCTCCAAATCGACATCGTTTACGGCGTGGACTACC
AGGGTATCTAATCCTGTTTGCTCCCCACGCTTTCGCACCTGAGCGTCAGTCTTTGTCCAGGGGGCCGCC
TTCGCCACCGGTATTCCTCCAGATCTCTACGCATTTCACCGCTACACCTGGAATTCTACCCCCCTCTACA
AGACTCTAGCCTGCCAGTTTCGAATGCAGTTCCCAGGTTGAGCCCGGGGATTTCACATCCGACTTGAC
AGACCGCCTGCGTGCGCTTTACGCCCAGTAATTCCCGATTAACGCTTGCACCCTCCGTATTACCGCGGC
TGCTGGCACGGAGTTAGCCGGTGCTTCTCTGCGGGTAACGTCAATCGACAAGGTTATTAACCTATCGCT
CTCCCCGCTGAAGTACTTACACCGAAGGCTTCTTCATAACACGCGGCATGGCTGCATCAGGCTT

Klebsiella quasipneumoniae subsp. similipneumoniae strain 07A044 16S ribosomal RNA, partial sequence	2008	2008	98%	0.0	98.93%	NR_134063.1
Klebsiella pneumoniae subsp. rhinoscleromatis strain R-70 16S ribosomal RNA gene, partial sequence	2008	2008	98%	0.0	99.02%	NR_037084.1
Klebsiella pneumoniae strain DSM 30104 16S ribosomal RNA gene, partial sequence	1986	1986	98%	0.0	98.67%	NR_117683.1
Klebsiella pneumoniae subsp. rhinoscleromatis strain ATCC 13884 16S ribosomal RNA gene, partial sequence	1986	1986	98%	0.0	98.67%	NR_114507.1
Klebsiella quasipneumoniae subsp. quasipneumoniae strain 01A030 16S ribosomal RNA, partial sequence	1984	1984	98%	0.0	98.49%	NR_134062.1
Klebsiella pneumoniae strain ATCC 13883 16S ribosomal RNA gene, partial sequence	1980	1980	98%	0.0	98.58%	NR_114506.1
Klebsiella pneumoniae strain NBRC 14940 16S ribosomal RNA gene, partial sequence	1971	1971	98%	0.0	98.31%	NR_113702.1
Klebsiella pneumoniae strain JCM 1662 16S ribosomal RNA gene, partial sequence	1971	1971	98%	0.0	98.31%	NR_113240.1
Klebsiella pneumoniae strain ATCC 13883 16S ribosomal RNA gene, partial sequence	1967	1967	98%	0.0	98.31%	NR_119278.1
Klebsiella pneumoniae strain JCM1662 16S ribosomal RNA gene, partial sequence	1965	1965	98%	0.0	98.31%	NR_112009.1

Klebsiella GL3

CCGCTGCATACAAGTGGTAGCGCCCTCCCGAAGGTTAAGCTACCTACTTCTTTTGCAACCCACTCCCAT
GGTGTGACGGGCGGTGTGTACAAGGCCCGGGAACGTATTCACCGTAGCATTCTGATCTACGATTACTA
GCGATTCCGACTTCATGGAGTCGAGTTGCAGACTCCAATCCGGACTACGACATACTTTATGAGGTCCGC
TTGCTCTCGCGAGGTCGCTTCTCTTTGTATGCGCCATTGTAGCACGTGTGTAGCCCTGGTCGTAAGGGC
CATGATGACTTGACGTCATCCCCACCTTCCTCCAGTTTATCACTGGCAGTCTCCTTTGAGTTCCCGGCC
TAACCGCTGGCAACAAAGGATAAGGGTTGCGCTCGTTGCGGGACTTAACCCAACATTTCACAACACG
AGCTGACGACAGCCATGCAGCACCTGTCTCACAGTTCCCGAAGGCACCAAAGCATCTCTGCTAAGTTC
TGTGGATGTCAAGACCAGGTAAGGTTCTTCGCGTTGCATCGAATTAAACCACATGCTCCACCGCTTGT
GCGGGCCCCCGTCAATTCATTTGAGTTTTAACCTTGCGGCCGTACTCCCCAGGCGGTCGATTTAACGCG
TTAGCTCCGGAAGCCACGCCTCAAGGGCACAACCTCCAAATCGACATCGTTTACGGCGTGGACTACCA
GGGTATCTAATCCTGTTTGCTCCCCACGCTTTCGCACCTGAGCGTCAGTCTTTGTCCAGGGGGCCGCCT
TCGCCACCGGTATTCCTCCAGATCTCTACGCATTTCACCGCTACACCTGGAATTCTACCCCCCTCTACAA
GACTCTAGCCTGCCAGTTTCGAATGCAGTTCCCAGGTTGAGCCCGGGGATTTCACATCCGACTTGACA
GACCGCCTGCGTGCGCTTTACGCCCAGTAATTCCGATTAACGCTTGCACCCTCCGTATTACCGCGGCTG
CTGGCACGGAGTTAGCCGGTGCTTCTTCTGCGGGTAACGTCAATCGACAAGGTTATTAACCTTATCGCT
TCTCCCGCTGAAAGTACTTTACCACCGAGCTCTCATACACGCGCATGGCTGCATCAGCTTGCGCCCATT
GGTGCAATATTCCCCCACTGCTGCTC

Klebsiella pneumoniae subsp. rhinoscleromatis strain R-70 16S ribosomal RNA gene, partial sequence	1899	1899	98%	0.0	98.22%	NR_037084.1
Klebsiella quasipneumoniae subsp. similipneumoniae strain 07A044 16S ribosomal RNA, partial sequence	1898	1898	98%	0.0	98.13%	NR_134063.1
Klebsiella quasipneumoniae subsp. quasipneumoniae strain 01A030 16S ribosomal RNA, partial sequence	1882	1882	98%	0.0	97.77%	NR_134062.1
Klebsiella pneumoniae strain DSM 30104 16S ribosomal RNA, partial sequence	1881	1881	98%	0.0	97.86%	NR_117683.1
Klebsiella pneumoniae strain JCM 1662 16S ribosomal RNA, partial sequence	1873	1873	98%	0.0	97.60%	NR_113240.1
Klebsiella pneumoniae strain NBRC 14940 16S ribosomal RNA, partial sequence	1870	1870	98%	0.0	97.60%	NR_113702.1
Klebsiella pneumoniae strain ATCC 13883 16S ribosomal RNA, partial sequence	1869	1869	98%	0.0	97.60%	NR_119278.1
Klebsiella pneumoniae strain DSM 30104 16S ribosomal RNA, partial sequence	1867	1867	98%	0.0	97.60%	NR_117686.1
Klebsiella pneumoniae strain JCM1662 16S ribosomal RNA, partial sequence	1867	1867	98%	0.0	97.60%	NR_112009.1
Klebsiella pneumoniae strain DSM 30104 16S ribosomal RNA, partial sequence	1865	1865	98%	0.0	97.51%	NR_114715.1
Enterobacter hormaechei subsp. xiangfangensis strain 10-17 16S ribosomal RNA, partial sequence	1863	1863	98%	0.0	97.51%	NR_126208.1
Klebsiella pneumoniae strain DSM 30104 16S ribosomal RNA, partial sequence	1863	1863	98%	0.0	97.51%	NR_117684.1
Klebsiella pneumoniae strain DSM 30104 16S ribosomal RNA, partial sequence	1861	1861	98%	0.0	97.51%	NR_036794.1

Klebsiella GL4

GGGAGTGGCGGCAGCTACACATGCAAGTCGAGCGGTAGCACAGAGAGCTTGCTCTCGGGTGACGAGC
GGCGGACGGGTGAGTAATGTCTGGGAAACTGCCTGATGGAGGGGGATAACTACTGGAAACGGTAGCT
AATACCGCATAACGTCGCAAGACCAAAGTGGGGGACCTTCGGGCCTCATGCCATCAGATGTGCCCAGA
TGGGATTAGCTAGTAGGTGGGGTAACGGCTCACCTAGGCGACGATCCCTAGCTGGTCTGAGAGGATGA
CCAGCCACACTGGAACTGAGACACGGTCCAGACTCCTACGGGAGGCAGCAGTGGGGAATATTGCACA
ATGGGCGCAAGCCTGATGCAGCCATGCCGCGTGTATGAAGAAGGCCTTCGGGTTGTAAAGTACTTTCA
GCGGGGAGGAAGGCGATAAGGTTAATAACCTTGTCGATTGACGTTACCCGCAGAAGAAGCACCGGCT
AACTCCGTGCCAGCAGCCGCGGTAATACGGAGGGTGCAAGCGTTAATCGGAATTACTGGGCGTAAAG
CGCACGCAGGCGGTCTGTCAAGTCGGATGTGAAATCCCCGGGCTCAACCTGGGAACTGCATTCGAAA
CTGGCAGGCTAGAGTCTTGTAGAGGGGGGTAGAATTCCAGGTGTAGCGGTGAAATGCGTAGAGATCTG

GAGGAATACCGGTGGCGAAGGCGGCCCCCTGGACAAAGACTGACGCTCAGGTGCGAAAGCGTGGGG
AGCAAACAGGATTAGATACCCTGGTAGTCCACGCCGTAAACGATGTCGATTTGGAGGTTGTGCCCTTG
AGGCGTGGCTTCCGGAGCTAACGCGTTAAATCGACCGCCTGGGGAGTACGGCCGCAAGGTTAAAACT
CAAATGAATTGACGGGGGCCCGCACAAGCGGTGGAGCATGTGGTTTAATTCGATGCAACGCGAAGAA
CCTTACCTGGTCTTGACATCCACAGAAACTTAGCAGAGATGCTTTGGTGCCTTCGGGAACTGTGAGAC
AGGTGCTGCATGGCTGTCGTCAGCTCGTGTGTGAAATGTTGGGGTAGTCCCGCACGAGCGCACCCTAT
CTTGTGCAGCGGTTCAGGCGGGAACTCAAAGGAGACTG

Klebsiella quasipneumoniae subsp. similipneumoniae strain 07A044 16S ribosomal RNA, partial sequence	2008	2008	98%	0.0	98.93%	NR_134063.1
Klebsiella pneumoniae subsp. rhinoscleromatis strain R-70 16S ribosomal RNA gene, partial sequence	2008	2008	98%	0.0	99.02%	NR_037084.1
Klebsiella pneumoniae strain DSM 30104 16S ribosomal RNA gene, partial sequence	1986	1986	98%	0.0	98.67%	NR_117683.1
Klebsiella pneumoniae subsp. rhinoscleromatis strain ATCC 13884 16S ribosomal RNA gene, partial sequence	1986	1986	98%	0.0	98.67%	NR_114507.1
Klebsiella quasipneumoniae subsp. quasipneumoniae strain 01A030 16S ribosomal RNA, partial sequence	1984	1984	98%	0.0	98.49%	NR_134062.1
Klebsiella pneumoniae strain ATCC 13883 16S ribosomal RNA gene, partial sequence	1980	1980	98%	0.0	98.58%	NR_114506.1
Klebsiella pneumoniae strain NBRC 14940 16S ribosomal RNA gene, partial sequence	1971	1971	98%	0.0	98.31%	NR_113702.1
Klebsiella pneumoniae strain JCM 1662 16S ribosomal RNA gene, partial sequence	1971	1971	98%	0.0	98.31%	NR_113240.1
Klebsiella pneumoniae strain ATCC 13883 16S ribosomal RNA gene, partial sequence	1967	1967	98%	0.0	98.31%	NR_119278.1

Klebsiella GL5

ACGCTGATCCAAGTGGTAGCGCCCTCCCGAAGGTTAAGCTACCTACTTCTTTTGCAACCCACTCCCATG
GTGTGACGGGCGGTGTGTACAAGGCCCGGGAACGTATTCACCGTAGCATTCTGATCTACGATTACTAGC
GATTCCGACTTCATGGAGTCGAGTTGCAGACTCCAATCCGGACTACGACATACTTTATGAGGTCCGCTT
GCTCTCGCGAGGTCGCTTCTCTTTGTATGCGCCATTGTAGCACGTGTGTAGCCCTGGTCGTAAGGGCCA
TGATGACTTGACGTCATCCCCACCTTCCTCCAGTTTATCACTGGCAGTCTCCTTTGAGTTCCCGGCCTA
ACCGCTGGCAACAAAGGATAAGGGTTGCGCTCGTTGCGGGACTTAACCCAACATTTCACAACACGAG
CTGACGACAGCCATGCAGCACCTGTCTCACAGTTCCCGAAGGCACCAAAGCATCTCTGCTAAGTTCTG
TGGATGTCAAGACCAGGTAAGGTTCTTCGCGTTGCATCGAATTAAACCACATGCTCCACCGCTTGTGC
GGGCCCCCGTCAATTCATTTGAGTTTTAACCTTGCGGCCGTACTCCCCAGGCGGTCGATTTAACGCGTT
AGCTCCGGAAGCCACGCCTCAAGGGCACAACCTCCAAATCGACATCGTTTACGGCGTGGACTACCAG
GGTATCTAATCCTGTTTGCTCCCCACGCTTTCGCACCTGAGCGTCAGTCTTTGTCCAGGGGGCCGCCTT
CGCCACCGGTATTCCTCCAGATCTCTACGCATTTCACCGCTACACCTGGAATTCTACCCCCCTCTACAA
GACTCTAGCCTGCCAGTTTCGAATGCAGTTCCCAGGTTGAGCCCGGGGATTTCACATCCGACTTGACA
GACCGCCTGCGTGCGCTTTACGCCCAGTAATTCCGATTAACGCTTGCACCCTCCGTATTACCGCGGCTG
CTGGCACGGAGTTAGCCGGTGCTTCTTCTGCGGGTAACGTCAATCGACAAGGTTATTACCTTATCGCTT
CTCCCCGCTGAAGTACTTACACCGAGCTCTCATACACGCGGCATGCTGCATCAGCTTGCGCCATTGTGC
AATATTCCCCCACCTG

Klebsiella pneumoniae subsp. rhinoscleromatis strain R-70 16S ribosomal RNA gene, partial sequence	1930	1930	98%	0.0	98.20%	NR_037084.1
Klebsiella quasipneumoniae subsp. similipneumoniae strain 07A044 16S ribosomal RNA, partial sequence	1929	1929	98%	0.0	98.11%	NR_134063.1
Klebsiella quasipneumoniae subsp. quasipneumoniae strain 01A030 16S ribosomal RNA, partial sequence	1908	1908	98%	0.0	97.75%	NR_134062.1
Klebsiella pneumoniae strain DSM 30104 16S ribosomal RNA gene, partial sequence	1908	1908	98%	0.0	97.84%	NR_117683.1
Klebsiella pneumoniae strain NBRC 14940 16S ribosomal RNA gene, partial sequence	1897	1897	98%	0.0	97.57%	NR_113702.1
Klebsiella pneumoniae strain JCM 1662 16S ribosomal RNA gene, partial sequence	1897	1897	98%	0.0	97.57%	NR_113240.1
Klebsiella pneumoniae strain ATCC 13883 16S ribosomal RNA gene, partial sequence	1893	1893	98%	0.0	97.57%	NR_119278.1
Klebsiella pneumoniae strain DSM 30104 16S ribosomal RNA gene, partial sequence	1892	1892	98%	0.0	97.57%	NR_117686.1
Klebsiella pneumoniae strain DSM 30104 16S ribosomal RNA gene, partial sequence	1892	1892	98%	0.0	97.48%	NR_114715.1
Klebsiella pneumoniae strain JCM1662 16S ribosomal RNA gene, partial sequence	1892	1892	98%	0.0	97.57%	NR_112009.1

Klebsiella GL6

GGGTCTTGGCGGCAGCTACACATGCAAGTCGAGCGGTAGCACAGAGAGCTTGCTCTCGGGTGACGAG
CGGCGGACGGGTGAGTAATGTCTGGGAAACTGCCTGATGGAGGGGGATAACTACTGGAAACGGTAGC
TAATACCGCATAACGTCGCAAGACCAAAGTGGGGGACCTTCGGGCCTCATGCCATCAGATGTGCCCAG
ATGGGATTAGCTAGTAGGTGGGGTAACGGCTCACCTAGGCGACGATCCCTAGCTGGTCTGAGAGGATG

ACCAGCCACACTGGAACTGAGACACGGTCCAGACTCCTACGGGAGGCAGCAGTGGGGAATATTGCAC
AATGGGCGCAAGCCTGATGCAGCCATGCCGCGTGTATGAAGAAGGCCTTCGGGTTGTAAAGTACTTTC
AGCGGGGAGGAAGGCGATAAGGTTAATAACCTTGTCGATTGACGTTACCCGCAGAAGAAGCACCGGC
TAACTCCGTGCCAGCAGCCGCGGTAATACGGAGGGTGCAAGCGTTAATCGGAATTACTGGGCGTAAAG
CGCACGCAGGCGGTCTGTCAAGTCGGATGTGAAATCCCCGGGCTCAACCTGGGAACTGCATTCGAAA
CTGGCAGGCTAGAGTCTTGTAGAGGGGGGTAGAATTCCAGGTGTAGCGGTGAAATGCGTAGAGATCTG
GAGGAATACCGGTGGCGAAGGCGGCCCCCTGGACAAAGACTGACGCTCAGGTGCGAAAGCGTGGGG
AGCAAACAGGATTAGATACCCTGGTAGTCCACGCCGTAAACGATGTCGATTTGGAGGTTGTGCCCTTG
AGGCGTGGCTTCCGGAGCTAACGCGTTAAATCGACCGCCTGGGGAGTACGGCCGCAAGGTTAAAACT
CAAATGAATTGACGGGGGCCCGCACAAGCGGTGGAGCATGTGGTTTAATTCGATGCAACGCGAAGAA
CCTTTACCTGGTCTTGACATCCACAGAACTTGGCAGAGATGCTTTGGTGCCTTCGGGAACTGTGAGAC
AGGTGCTGCATGGCTGTCGTCAGCTCGTGTGTGAAATGTTGGGTTAAGTCCGCACGAGCGCAACCCTT
ATCCTTTGTTGCCAGGCGGTTTCGGGCCGGGAACTTCAAGGAAGACTGGCCAGGTGA

Klebsiella quasipneumoniae subsp. similipneumoniae strain 07A044 16S ribosomal RNA, partial sequence	1991	1991	99%	0.0	98.42%	NR_134063.1
Klebsiella pneumoniae subsp. rhinoscleromatis strain R-70 16S ribosomal RNA gene, partial sequence	1980	1980	99%	0.0	98.33%	NR_037084.1
Klebsiella quasipneumoniae subsp. quasipneumoniae strain 01A030 16S ribosomal RNA, partial sequence	1967	1967	99%	0.0	97.98%	NR_134062.1
Klebsiella pneumoniae strain DSM 30104 16S ribosomal RNA gene, partial sequence	1964	1964	99%	0.0	98.06%	NR_117683.1
Klebsiella pneumoniae subsp. rhinoscleromatis strain ATCC 13884 16S ribosomal RNA gene, partial sequence	1964	1964	99%	0.0	98.06%	NR_114507.1
Klebsiella pneumoniae strain ATCC 13883 16S ribosomal RNA gene, partial sequence	1958	1958	99%	0.0	97.98%	NR_114506.1
Klebsiella pneumoniae strain NBRC 14940 16S ribosomal RNA gene, partial sequence	1949	1949	99%	0.0	97.71%	NR_113702.1
Klebsiella pneumoniae strain JCM 1662 16S ribosomal RNA gene, partial sequence	1949	1949	99%	0.0	97.71%	NR_113240.1
Klebsiella pneumoniae subsp. ozaenae strain ATCC 11296 16S ribosomal RNA gene, partial sequence	1947	1947	99%	0.0	97.71%	NR_119276.1
Klebsiella pneumoniae strain ATCC 13883 16S ribosomal RNA gene, partial sequence	1945	1945	99%	0.0	97.71%	NR_119278.1

Klebsiella GL7

TAAATTTGATCACAAGTGGTAAGCGCCCTCCCGAAGGTTAAGCTACCTACTTCTTTTGCAACCCACTCC
CATGGTGTGACGGGCGGTGTGTACAAGGCCCGGGAACGTATTCACCGTAGCATTCTGATCTACGATTAC
TAGCGATTCCGACTTCATGGAGTCGAGTTGCAGACTCCAATCCGGACTACGACGTACTTTATGAGGTCC
GCTTGCTCTCGCGAGGTCGCTTCTCTTTGTATGCGCCATTGTAGCACGTGTGTAGCCCTGGTCGTAAGG
GCCATGATGACTTGACGTCATCCCCACCTTCCTCCAGTTTATCACTGGCAGTCTCCTTTGAGTTCCCGG
CCTAACCGCTGGCAACAAAGGATAAGGGTTGCGCTCGTTGCGGGACTTAACCCAACATTTCACAACAC
GAGCTGACGACAGCCATGCAGCACCTGTCTCACAGTTCCCGAAGGCACCAAAGCATCTCTGCTAAGT
TCTGTGGATGTCAAGACCAGGTAAGGTTCTTCGCGTTGCATCGAATTAAACCACATGCTCCACCGCTTG
TGCGGGCCCCCGTCAATTCATTTGAGTTTTAACCTTGCGGCCGTACTCCCCAGGCGGTCGATTTAACGC
GTTAGCTCCGGAAGCCACGCCTCAAGGGCACAACCTCCAAATCGACATCGTTTACGGCGTGGACTACC
AGGGTATCTAATCCTGTTTGCTCCCCACGCTTTCGCACCTGAGCGTCAGTCTTTGTCCAGGGGGCCGCC
TTCGCCACCGGTATTCCTCCAGATCTCTACGCATTTCACCGCTACACCTGGAATTCTACCCCCCTCTACA
AGACTCTAGCCTGCCAGTTTCGAATGCAGTTCCCAGGTTGAGCCCGGGGATTTCACATCCGACTTGAC
AGACCGCCTGCGTGCGCTTTACGCCCAGTAATTCCCGATTAACGCTTGCACCCTCCGTATTACCGCGGC
TGCTGGCACGGAGTTAGCCGGTGCTTCTTCTGCGGGTAACGTCAATCGACAAGGTATAACCTATCGCTT
TCCTCCCCGCTGAAAGTACTTACAACCGAGCTTCTCATACACGCGGCATGCCTGCATCAGGCCTGGCG
CCAATGGTGCAATATTCCCCCAACTTTGCTGCCTCCCCGTT

Klebsiella pneumoniae subsp. rhinoscleromatis strain R-70 16S ribosomal RNA gene, partial sequence	1892	1892	98%	0.0	97.45%	NR_037084.1
Klebsiella quasipneumoniae subsp. similipneumoniae strain 07A044 16S ribosomal RNA, partial sequence	1891	1891	98%	0.0	97.36%	NR_134063.1
Klebsiella quasipneumoniae subsp. quasipneumoniae strain 01A030 16S ribosomal RNA, partial sequence	1874	1874	98%	0.0	97.01%	NR_134062.1
Klebsiella pneumoniae strain DSM 30104 16S ribosomal RNA, partial sequence	1874	1874	98%	0.0	97.10%	NR_117683.1
Klebsiella pneumoniae strain JCM 1662 16S ribosomal RNA, partial sequence	1868	1868	98%	0.0	96.92%	NR_113240.1
Klebsiella pneumoniae strain NBRC 14940 16S ribosomal RNA, partial sequence	1866	1866	98%	0.0	96.92%	NR_113702.1
Klebsiella pneumoniae strain ATCC 13883 16S ribosomal RNA, partial sequence	1866	1866	98%	0.0	96.92%	NR_119278.1
Enterobacter hormaechei subsp. xiangfangensis strain 10-17 16S ribosomal RNA, partial sequence	1865	1865	98%	0.0	96.92%	NR_126208.1
Klebsiella pneumoniae strain DSM 30104 16S ribosomal RNA, partial sequence	1865	1865	98%	0.0	96.92%	NR_117686.1
Klebsiella pneumoniae strain JCM1662 16S ribosomal RNA, partial sequence	1865	1865	98%	0.0	96.92%	NR_112009.1
Klebsiella pneumoniae strain DSM 30104 16S ribosomal RNA, partial sequence	1863	1863	98%	0.0	96.84%	NR_114715.1
[illegible]	[illegible]	[illegible]	[illegible]	[illegible]	[illegible]	[illegible]

Lactococcus GL1

AAGCCCTGGGGACAGCCTACCAATGCAGTCGAGCGCCGAAGGTTGGTACTTGTACCGACTGGATGAG
CTGCGAACGGGTGATTAACGCGTGGGGAATCTGCCTTTGAGCGGGAGACAACATTTGGAAACCAATG
CTAATACCGCATATAAACTTTATGCACAAGTTTTAAGTTTGAAAGATGCTCTTGCATCACTCCAAGATGA
TCCCGCGTTGTATTATCTAGTTGGTGAGGTAAAGGCTCACCAAGGCGATGATACATAGCCGACCTGACA
GGGTGATCGGCCACATTGGGACTGACACACGGCCCATACTCCTACCTCAGGCAGCAATAAGGAATCTT
TGTGAATGGACTAAAGTCTGACCGACCAACGCCCCGTGAATGAAGAAGGTTTTCGGATCATAAAACTC
TGTTGGTAAAATCGATCATTGGTGAGAGTGGAAAGCTCATCACGTGACCTTCCTACAACTCAAAGCCC
GGTTAATTACTTGCCATCAGCCGCTGTAATACGGGTGTCCCGAACGCTGTCCGGATTTATTGGGCGCTA
GCGAGCGCAGGTGCTTTATTACTCTGGAATACCAAGCACTGACTCACACAATTGAATGCATTGGAGAC
TGGTAGACTTGATGCAGGAAAGGAGAGTGCAATTCCATGTGTAGAGGTGAAATGCATAGATATATGGA
AGAACACCGGTGGGAAAGCTGTCTCTGGCCTGTGAACTGACACTGAGGCTCGAAGGCGTGGAGAGC
AAACTAGATTAGATACCCTGCCACTCCACGCCCTATACGATGAGTGCTCTATGTAGGGAGCTATAAGTC
TCTCTGTATCACAGCTAACTCAATAATCACTCCTGCGCTGGGGAGTACGACCGAATTGATTGACACTCA
AGTGAATTGGCGGCGGGCCCTCGCAGCGGGGGGAGCGTGTTGATTTATTCCTAACTACGCAACCATCC
TAACAGTGCATGCCCTACTGCCCGCCTATCCAAACGATGCTAACTTCCGTACCGGATACGACATACAGG
TGCT

Lactococcus lactis strain NBRC 100933 16S ribosomal RNA gene, partial sequence	1024	1024	88%	0.0	87.01%	NR_113960.1
Lactococcus lactis strain NCDO 604 16S ribosomal RNA gene, partial sequence	1024	1024	88%	0.0	87.01%	NR_040955.1
Lactococcus lactis subsp. hordniae strain NBRC 100931 16S ribosomal RNA gene, partial sequence	1013	1013	88%	0.0	86.80%	NR_113958.1
Lactococcus lactis subsp. hordniae strain NCDO 2181 16S ribosomal RNA gene, partial sequence	1013	1013	88%	0.0	86.80%	NR_040956.1
Lactococcus lactis subsp. tructae strain L105 16S ribosomal RNA gene, partial sequence	974	974	88%	0.0	86.04%	NR_116443.1
Lactococcus lactis subsp. cremoris strain NBRC 100676 16S ribosomal RNA gene, partial sequence	968	968	88%	0.0	85.93%	NR_113925.1
Lactococcus lactis subsp. cremoris strain NCDO 607 16S ribosomal RNA gene, partial sequence	968	968	88%	0.0	85.93%	NR_040954.1
Lactococcus taiwanensis strain 0905C15 16S ribosomal RNA gene, partial sequence	952	952	88%	0.0	85.61%	NR_114327.1
Lactococcus hircilactis strain DSM 28960 16S ribosomal RNA, partial sequence	785	785	88%	0.0	82.51%	NR_136465.1
Lactococcus nasutitermitis strain M19 16S ribosomal RNA, partial sequence	763	763	87%	0.0	82.38%	NR_147780.1

Burkholderia GL1

GGGCCGTGGCGGCTGCTTACACATGCAGTCGAACGGCAGCACGGGTGCTTGCACCTGGTGGCGAGTG
GCGAACGGGTGAGTAATACATCGGAACGTGTCCTGTAGTGGGGGATAGCCCGGCGAAAGCCGGATTA
ATACCGCATACGATCTACGGATGAAAGCGGGGGATCTTCGGACCTCGCGCTATAGGGGCGGCCGATGG
CGGATTAGCTAGTTGGTGAGGTAAAGGCTCACCAAGGCGACGATCCGTAGCTGGTCTGAGAGGACGA
CCAGCCACACTGGGACTGAGACACGGCCCAGACTCCTACGGGAGGCAGCAGTGGGGAATTTTGGACA
ATGGGCGAAAGCCTGATCCAGCAATGCCGCGTGTGTGAAGAAGGCCTTCGGGTTGTAAAGCACTTTTG
TCCGGAAAGAAATCCCTGGTCCTAATATGGCCGGGGGATGACGGTACCGGAAGAATAAGCACCGGCTA
ACTACGTGCCAGCAGCCGCGGTAATACGTAGGGTGCAAGCGTTAATCGGAATTACTGGGCGTAAAGCG
TGCGCAGGCGGTGATGTAAGACCGATGTGAAATCCCCGGGCTCAACCTGGGAACTGCATTGGTGACTG
CATCGCTTGAGTATGGCAGAGGGGGGTAGAATTCCACGTGTAGCAGTGAAATGCGTAGAGATGTGGAG
GAATACCGATGGCGAAGGCAGCCCCCTGGGTCAATACTGACGCTCATGCACGAAAGCGTGGGGAGCA
AACAGGATTAGATACCCTGGTAGTCCACGCCCTAAACGATGTCAACTGGTTGTCGGGTCTTCATTGACT

TGGTAACGTAGCTACGCGTGAAGTTGACCGCCTGGGGAGTACGGTCGCAAGATTAAAACTCAAAGGA
ATTTGACGGGGACCCGCACAAGCGGTGGATGATGTGGATTAATTTCGATGCAACGCGAAAAACCTTAC
CTACCCTTGACATGTACGGAAATTCTGCTGAGGAGGTGAAGTGCCCGAAAAGGAGCCGTAACACAGT
GCTGCATGGCTGTCGTCAGCTCGTGTCGTGAGATGTTGGGTAGTCCCGCACGAGCGCACCCTTGTCCC
TAGTGCTACGCAGAGCACTCAGCAGACTGGCGTGACAAACGGAAGAAGTGGGATGACGTTCAGGTCC
TCATGCTATGGATAAGCTCAACGTCTATACAATGGTTCCGGAC

Burkholderia tropica strain Ppe8 16S ribosomal RNA gene, partial sequence	1978	1978	98%	0.0	96.76%	NR_028965.1
Burkholderia bannensis strain E25 16S ribosomal RNA gene, partial sequence	1884	1884	98%	0.0	95.36%	NR_113178.1
Burkholderia tropica strain LMG 22274 16S ribosomal RNA gene, partial sequence	1881	1881	91%	0.0	97.65%	NR_118080.1
Burkholderia metalliresistens strain D414 16S ribosomal RNA, partial sequence	1871	1871	98%	0.0	95.18%	NR_136833.1
Burkholderia unamae strain MTI-641 16S ribosomal RNA gene, partial sequence	1862	1862	98%	0.0	95.02%	NR_027569.1
'Burkholderia humi' Srinivasan et al. 2013 strain Rs7 16S ribosomal RNA, partial sequence	1853	1853	98%	0.0	94.93%	NR_132708.1
Burkholderia eburnea strain RR11 16S ribosomal RNA, partial sequence	1842	1842	98%	0.0	94.76%	NR_133712.1
Paraburkholderia oxyphila clone pCR2.1::OX-01_rDNA#2 16S ribosomal RNA, partial sequence	1823	1823	98%	0.0	94.44%	NR_112884.1
Burkholderia oxyphila strain OX-01 16S ribosomal RNA gene, partial sequence	1821	1821	98%	0.0	94.44%	NR_112886.1
Burkholderia oxyphila strain OX-01 16S ribosomal RNA gene, partial sequence	1818	1818	98%	0.0	94.36%	NR_112885.1

***Burkholderia* GL2**

GGCCGTGGGGGCATGCTTACCATGCAGTCGAACGGCAGCACGGGTGCTTGCACCTGGTGGCGAGTGG
CGAACGGGTGAGTAATACATCGGAACGTGTCCTGTAGTGGGGGATAGCCCGGCGAAAGCCGGATTAAT
ACCGCATACGATCTACGGATGAAAGCGGGGGATCTTCGGACCTCGCGCTATAGGGGCGGCCGATGGCG
GATTAGCTAGTTGGTGAGGTAAAGGCTCACCAAGGCGACGATCCGTAGCTGGTCTGAGAGGACGACC
AGCCACACTGGGACTGAGACACGGCCCAGACTCCTACGGGAGGCAGCAGTGGGGAATTTTGGACAAT
GGGCGAAAGCCTGATCCAGCAATGCCGCGTGTGTGAAGAAGGCCTTCGGGTTGTAAAGCACTTTTGT
CCGGAAAGAAATCCCTGGTCCTAATATGGCCGGGGGATGACGGTACCGGAAGAATAAGCACCGGCTA
ACTACGTGCCAGCAGCCGCGGTAATACGTAGGGTGCAAGCGTTAATCGGAATTACTGGGCGTAAAGCG
TGCGCAGGCGGTGATGTAAGACCGATGTGAAATCCCCGGGCTCAACCTGGGAACTGCATTGGTGACTG
CATCGCTTGAGTATGGCAGAGGGGGGTAGAATTCCACGTGTAGCAGTGAAATGCGTAGAGATGTGGAG
GAATACCGATGGCGAAGGCAGCCCCCTGGGTCAATACTGACGCTCATGCACGAAAGCGTGGGGAGCA
AACAGGATTAGATACCCTGGTAGTCCACGCCCTAAACGATGTCAACTGGTTGTCGGGTCTTCATTGACT
TGGTAACGTAGCTAACGCGTGAAGTTGACCGCCTGGGGAGTACGGTCGCAAGATTAAAACTCAAAGG
AATTGACGGGGACCCGCACAAGCGGTGGATGATGTGGATTAATTCGATGCAACGCGAAAAACCTTACC
TACCCTTGACATGTACGGAATTTCTGCTGAGAGGTGGAAGTGCCCGAAAGGGAGCCGTAACACAGGT
GCTGCATGGCTGTCGTCAGCTCGTGTCGTGAGATGTTGGGTTAGTCCGCACGAGCGCACCCTGTCCTA
GTGCTACGCAGAGCACTCCAGGAGACTGCCGTGACCACGAGAGGTGGGGATGACGTCAGTCTCATGC
CTTATGGGCTAGGCTTCAACGGTCATACATGGTCGGAACAGAGGATTGGCCAAGCCCGCAGT

Burkholderia tropica strain Ppe8 16S ribosomal RNA gene, partial sequence	2050	2050	99%	0.0	97.30%	NR_028965.1
Burkholderia bannensis strain E25 16S ribosomal RNA gene, partial sequence	1956	1956	99%	0.0	95.93%	NR_113178.1
Burkholderia metalliresistens strain D414 16S ribosomal RNA, partial sequence	1943	1943	99%	0.0	95.75%	NR_136833.1
'Burkholderia humi' Srinivasan et al. 2013 strain Rs7 16S ribosomal RNA, partial sequence	1941	1941	99%	0.0	95.74%	NR_132708.1
Burkholderia unamae strain MTI-641 16S ribosomal RNA gene, partial sequence	1934	1934	99%	0.0	95.59%	NR_027569.1
Burkholderia tropica strain LMG 22274 16S ribosomal RNA gene, partial sequence	1923	1923	91%	0.0	97.86%	NR_118080.1
Burkholderia eburnea strain RR11 16S ribosomal RNA, partial sequence	1914	1914	99%	0.0	95.34%	NR_133712.1
Paraburkholderia oxyphila clone pCR2.1::OX-01_rDNA#2 16S ribosomal RNA, partial sequence	1890	1890	99%	0.0	94.94%	NR_112884.1
Burkholderia oxyphila strain OX-01 16S ribosomal RNA gene, partial sequence	1884	1884	99%	0.0	94.86%	NR_112886.1
Burkholderia oxyphila strain OX-01 16S ribosomal RNA gene, partial sequence	1884	1884	99%	0.0	94.86%	NR_112885.1
Burkholderia oxyphila strain NBRC 105797 16S ribosomal RNA gene, partial sequence	1873	1873	99%	0.0	94.62%	NR_114289.1

***Burkholderia* GL3**

TCAGTGGCCGCATGCTTACACATGCAGTCGAACGGCAGCACGGGTGCTTGCACCTGGTGGCGAGTGG

CGAACGGGTGAGTAATACATCGGAACGTGTCCTGTAGTGGGGGATAGCCCGGCGAAAGCCGGATTAAT
ACCGCATACGATCTACGGATGAAAGCGGGGGATCTTCGGACCTCGCGCTATAGGGGCGGCCGATGGCG
GATTAGCTAGTTGGTGAGGTAAAGGCTCACCAAGGCGACGATCCGTAGCTGGTCTGAGAGGACGACC
AGCCACACTGGGACTGAGACACGGCCCAGACTCCTACGGGAGGCAGCAGTGGGGAATTTTGGACAAT
GGGCGAAAGCCTGATCCAGCAATGCCGCGTGTGTGAAGAAGGCCTTCGGGTTGTAAAGCACTTTTGT
CCGGAAAGAAATCCCTGGTCCTAATATGGCCGGGGGATGACGGTACCGGAAGAATAAGCACCGGCTA
ACTACGTGCCAGCAGCCGCGGTAATACGTAGGGTGCAAGCGTTAATCGGAATTACTGGGCGTAAAGCG
TGCGCAGGCGGTGATGTAAGACCGATGTGAAATCCCCGGGCTCAACCTGGGAACTGCATTGGTGACTG
CATCGCTTGAGTATGGCAGAGGGGGGTAGAATTCCACGTGTAGCAGTGAAATGCGTAGAGATGTGGAG
GAATACCGATGGCGAAGGCAGCCCCCTGGGTCAATACTGACGCTCATGCACGAAAGCGTGGGGAGCA
AACAGGATTAGATACCCTGGTAGTCCACGCCCTAAACGATGTCAACTGGTTGTCGGGTCTTCATTGACT
TGGTAACGTAGCTAACGCGTGAAGTTGACCGCCTGGGGAGTACGGTCGCAAGATTAAAACTCAAAGG
AATTGACGGGGACCCGCACAAGCGGTGGATGATGTGGATTAATTCGATGCAACGCGAAAAACCTTACC
TACCCTTGACATGTACGGAATTCCTGCTGAGAGGTGGAAGTGCCCGAAAGGGAGCCGTAACACAGGT
GCTGCATGGCTGTCGTCAGCTCGTGTCGTGAGATGTGGGTTAGTCCCGCACGAGCGCACCTTGTCCTT
AGTGCTACGCAGAGCACTCCAGGAGACTGGCGTTGACAACGTAGAGGTGGGATGACGTCAGTCTCAT
GGCTATGTAGCTTCAACGTCATAACAATTGTTCGGAAACCAGAGGTC

Burkholderia tropica strain Ppe8 16S ribosomal RNA gene, partial sequence	2050	2050	99%	0.0	97.30%	NR_028965.1
Burkholderia bannensis strain E25 16S ribosomal RNA gene, partial sequence	1956	1956	99%	0.0	95.93%	NR_113178.1
Burkholderia metalliresistens strain D414 16S ribosomal RNA, partial sequence	1943	1943	99%	0.0	95.75%	NR_136833.1
'Burkholderia humi' Srinivasan et al. 2013 strain Rs7 16S ribosomal RNA, partial sequence	1941	1941	99%	0.0	95.74%	NR_132708.1
Burkholderia unamae strain MTI-641 16S ribosomal RNA gene, partial sequence	1934	1934	99%	0.0	95.59%	NR_027569.1
Burkholderia tropica strain LMG 22274 16S ribosomal RNA gene, partial sequence	1923	1923	91%	0.0	97.86%	NR_118080.1
Burkholderia eburnea strain RR11 16S ribosomal RNA, partial sequence	1914	1914	99%	0.0	95.34%	NR_133712.1
Paraburkholderia oxyphila clone pCR2.1::OX-01_rDNA#2 16S ribosomal RNA, partial sequence	1890	1890	99%	0.0	94.94%	NR_112884.1
Burkholderia oxyphila strain OX-01 16S ribosomal RNA gene, partial sequence	1884	1884	99%	0.0	94.86%	NR_112886.1
Burkholderia oxyphila strain OX-01 16S ribosomal RNA gene, partial sequence	1884	1884	99%	0.0	94.86%	NR_112885.1
Burkholderia oxyphila strain NBRC 105797 16S ribosomal RNA gene, partial sequence	1873	1873	99%	0.0	94.62%	NR_114289.1

Burkholderia GL4

CAAGGGCGGCATGCTTAACATGCAAGTCGAACGGCAGCACGGGTGCTTGCACCTGGTGGCGAGTGGC
GAACGGGTGAGTAATACATCGGAACGTGTCCTGTAGTGGGGGATAGCCCGGCGAAAGCCGGATTAATA
CCGCATACGATCTACGGATGAAAGCGGGGGATCTTCGGACCTCGCGCTATAGGGGCGGCCGATGGCGG
ATTAGCTAGTTGGTGAGGTAAAGGCTCACCAAGGCGACGATCCGTAGCTGGTCTGAGAGGACGACCA
GCCACACTGGGACTGAGACACGGCCCAGACTCCTACGGGAGGCAGCAGTGGGGAATTTTGGACAATG
GGCGAAAGCCTGATCCAGCAATGCCGCGTGTGTGAAGAAGGCCTTCGGGTTGTAAAGCACTTTTGTCC
GGAAAGAAATCCCTGGTCCTAATATGGCCGGGGGATGACGGTACCGGAAGAATAAGCACCGGCTAACT
ACGTGCCAGCAGCCGCGGTAATACGTAGGGTGCAAGCGTTAATCGGAATTACTGGGCGTAAAGCGTGC
GCAGGCGGTGATGTAAGACCGATGTGAAATCCCCGGGCTCAACCTGGGAACTGCATTGGTGACTGCAT
CGCTTGAGTATGGCAGAGGGGGGTAGAATTCCACGTGTAGCAGTGAAATGCGTAGAGATGTGGAGGA
ATACCGATGGCGAAGGCAGCCCCCTGGGTCAATACTGACGCTCATGCACGAAAGCGTGGGGAGCAAA
CAGGATTAGATACCCTGGTAGTCCACGCCCTAAACGATGTCAACTGGTTGTCGGGTCTTCATTGACTTG
GTAACGTAGCTAACGCGTGAAGTTGACCGCCTGGGGAGTACGGTCGCAAGATTAAAACTCAAAGGAA
TTGACGGGGACCCGCACAAGCGGTGGATGATGTGGATTAATTCGATGCAACGCGAAAAACCTTACCTA
CCCTTGACATGTACGGAATCCCGCTGAGAGGTGGAAGTGCTCGAAAGGGAGCCGTAACACAGGTGCT
GCATGGCTGTCGTCAGCTCGTGTCGTGAGATGTTGGGGTTAAGTCCCGCAACGAGCGCAACCCTTGTC
CCTAGTGCTACGCAGAGCACCTCCAGGAGACTGCGTTGACAACGAGAGGTGGGATGACGTCAGTCTC

ATGCCCTATGATAAGCTCAACGTCATACATGTTCGACAGGAGCCAAGCGCAAGTTGAGCCATCCCAAA
ACCGATCCTAATTTCCG

Burkholderia tropica strain Ppe8 16S ribosomal RNA gene, partial sequence	2043	2043	98%	0.0	96.57%	NR_028965.1
Burkholderia bannensis strain E25 16S ribosomal RNA gene, partial sequence	1949	1949	98%	0.0	95.22%	NR_113178.1
Burkholderia tropica strain LMG 22274 16S ribosomal RNA gene, partial sequence	1934	1934	88%	0.0	98.29%	NR_118080.1
'Burkholderia humi' Srinivasan et al. 2013 strain Rs7 16S ribosomal RNA, partial sequence	1930	1930	98%	0.0	94.97%	NR_132708.1
Burkholderia metalliresistens strain D414 16S ribosomal RNA, partial sequence	1927	1927	98%	0.0	94.89%	NR_136833.1
Burkholderia unamae strain MTI-641 16S ribosomal RNA gene, partial sequence	1927	1927	98%	0.0	94.89%	NR_027569.1
Burkholderia eburnea strain RR11 16S ribosomal RNA, partial sequence	1884	1884	98%	0.0	94.32%	NR_133712.1
Paraburkholderia oxyphila clone pCR2.1::OX-01_rDNA#2 16S ribosomal RNA, partial sequence	1882	1882	98%	0.0	94.25%	NR_112884.1
Burkholderia oxyphila strain OX-01 16S ribosomal RNA gene, partial sequence	1881	1881	95%	0.0	95.40%	NR_112886.1
Burkholderia oxyphila strain OX-01 16S ribosomal RNA gene, partial sequence	1877	1877	98%	0.0	94.18%	NR_112885.1
Burkholderia oxyphila strain NBRC 105797 16S ribosomal RNA gene, partial sequence	1866	1866	98%	0.0	93.94%	NR_114289.1

Rhizobium GL1

TAACCTATTCGCAGCTACACTGCAGTCGAGCGCACTTTTGGGTCCTTGTAACGACTGCAGGACCCGGG
AACGCATGAGCTTTCGAGGGAAATCTGCCTGTAAACTGGATTTAATATTTGATAACCCCTGCTAAATAC
AAGAAATGACCTTAAACGATTGTCCCACGTTGGATTAATGCTCTGGGTGTTGTAGATGGTGATGCCGCG
TTGTATCCACTTGGTGGTGAAAGGATCATCAAGGAGGCTGGGCCTGATAGACCTGATGACACCTAGAT
CCACGCTGTTGGGAGAATAACACCCCCCTGCCTCCTACCTGATCCAGCCATGCCGAATGAGTGAGGAT
GGCCTTAAGGTTGTAAAACTCTTTCCCCGTGAATGATAAAGGCTTTCGGATCAGATCAATCCCCGGGTA
ACTTCGAGTCCTCAAGCGAGTGAATACAAAGGGGCCTAACCTGGTACGAAATTCCTGGCTGTAAACGG
GACATCAGCCGATATTATAGTCAGGGGGCAAATCCCGCCCCTAAACTGTGGTTGTGCCTTTGATACTGG
GTATCTTGAGTATGTGTCAGGTAAACTCAGATCTCAACTGTACAGTTGAAATTGATACATATTGGTATGA
TCGCCGAAGAGGAAGGTGGAATTCCGTGTCCATTGCTGACAATGCATTGAGATGAGCTAGGAACAGC
ACACCGGGATGGCATACTCCTGTGCACTGTCCTTG
ACACTGATGCGATGATAGCCGGCGGAGCAGAATAAGATTAGATGGCCTGGGCTGTCCACTCTATACACT
ATGCCTTGGTGAGTTAGGTGTGGAGATTATATACTCAGTGGAAATCTCTAGCTCATCAAACAATCCGTG
CGGCCGGGTGAGTTTCGACCGAATGGACGCTGAATAGGAATTGACTGGAGCATCCGCCTCGACGGGT
GGGGGGCATTGGTGTTAATTTGATACTATCAACTCAGGCAAACCCTCACCTAGCCGTTGCCTGGACCGT
CCTGACGGCCCCTCCAATACGGGAGTATGCTTCCGTTCAAGACCGCTACAAGCAGGGCGACTGCTTTG
CCTTTACATTCCCCTTCTTTAAGATGGATGAATCTTTAGTCCCCCCCCCCCGACCGACACCCCCCTCGA
GAGCTATGATGTGGGCTGACT

Rhizobium wenxiniae strain 166 16S ribosomal RNA, partial sequence	137	137	21%	1e-31	76.86%	NR_157780.1
Rhizobium zeae strain CRZM18R 16S ribosomal RNA, partial sequence	137	137	21%	1e-31	76.86%	NR_157662.1
Agrobacterium salinitolerans strain YIC 5082 16S ribosomal RNA, partial sequence	137	137	21%	1e-31	76.86%	NR_157010.1
Agrobacterium fabrum strain C58 16S ribosomal RNA, partial sequence	137	137	21%	1e-31	76.86%	NR_074266.1
Rhizobium pusense strain NRCPB10 16S ribosomal RNA gene, partial sequence	137	137	21%	1e-31	76.86%	NR_116874.1
Rhizobium kunmingense strain LXD30 16S ribosomal RNA, partial sequence	137	137	21%	1e-31	76.86%	NR_132597.1
Rhizobium cellulosilyticum strain ALA10B2 16S ribosomal RNA gene, partial sequence	137	137	21%	1e-31	76.86%	NR_043985.1
Rhizobium smilacinae strain PTYR-5 16S ribosomal RNA, partial sequence	126	126	21%	2e-28	76.08%	NR_148270.1
Rhizobium daejeonense strain NBRC 102495 16S ribosomal RNA gene, partial sequence	126	126	21%	2e-28	76.08%	NR_114121.1
Rhizobium naphthalenivorans strain TSY03b 16S ribosomal RNA, partial sequence	126	126	21%	2e-28	76.08%	NR_132669.1
Rhizobium helanshanense strain Qtx-14 16S ribosomal RNA, partial sequence	126	126	21%	2e-28	75.98%	NR_133019.1
